# Supplementary material for: Manipulating Zika virus RNA tertiary structure for developing tissue-specific attenuated vaccines
Source: EMBO Mol Med. 2025 Sep 8;17(10):2787–808. doi: 10.1038/s44321-025-00304-5 (PMC12514043; doi:10.1038/s44321-025-00304-5)
Supplement: Supplementary file 1 — Appendix [file 44321_2025_304_MOESM1_ESM.pdf]

Appendix figures for

Manipulating Zika virus RNA tertiary structure for developing tissue-specific attenuated vaccines

Xiang Chen *et al.*

Corresponding author: Cheng-Feng Qin, qincf@bmi.ac.cn

Table of contents

Appendix Fig. S1. Viral RNA loads in different organs of infected A129 mice...........2

Appendix Fig. S2. The transcriptome analyses of mouse brains after WT or MBD2 ZIKV infection...3

Appendix Fig. S3. Stability analysis of the MBD1 and MBD2 in vitro and in vivo.....5

Appendix Fig. S4. Monkeys used in this study were negative for flavivirus before vaccination...6

Appendix Fig. S1

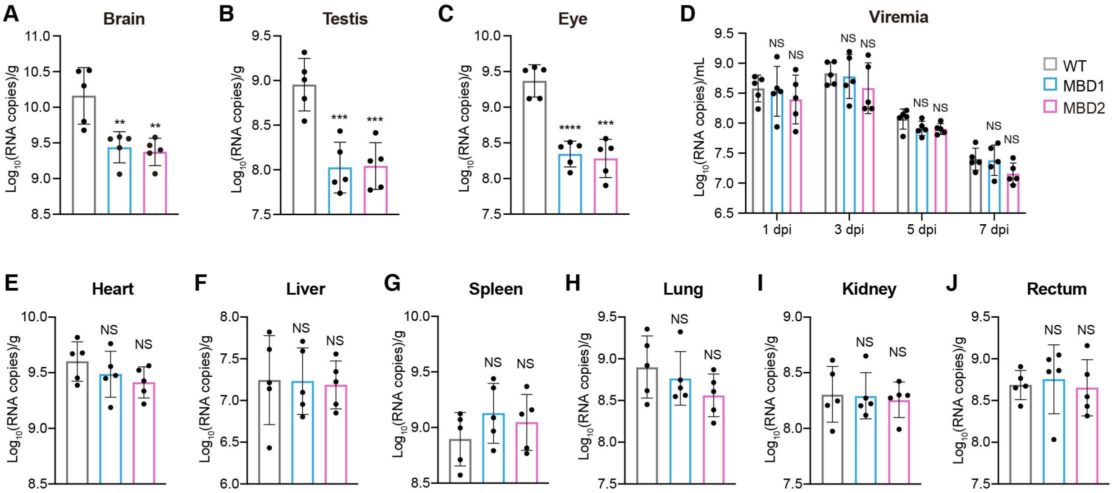

Appendix Fig. S1. Viral RNA loads in different organs of infected A129 mice.

Four-week-old A129 mice were infected with 1 × 10^4 PFU of WT ZIKV, MBD1 or MBD2. Organs from infected mice were collected and homogenized on 5dpi. The amounts of viral RNA were quantified by RT-qPCR. Data are the mean ± SD. n=5. n represents biological replicates. Two-sided Student's *t* test. **P < 0.01, ***P < 0.001 (A: MBD1 P=0.0073, MBD2 P=0.0039; B: MBD1 P=0.0010, MBD2 P=0.0009; C: MBD1 P=0.000049, MBD2 P=0.0001), NS, not significant.

Appendix Fig. S2

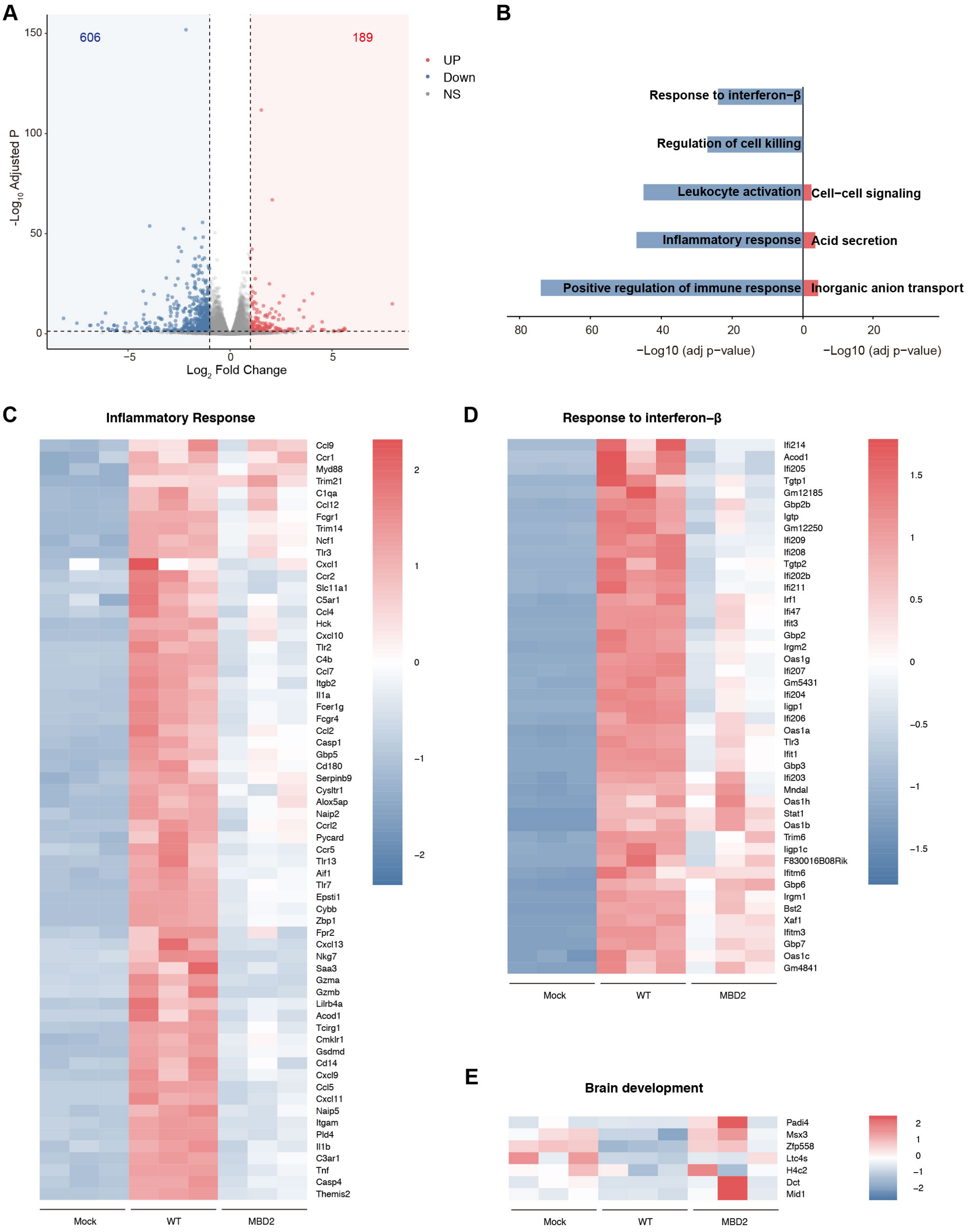

Appendix Fig. S2. The transcriptome analyses of mouse brains after WT or MBD2 ZIKV infection.

One-day-old suckling CD-1 mice (n = 3/group) were i.c. inoculated with 1000 PFU of indicated viruses. On days 3 post inoculation, total RNA from brains were extracted for RNA-seq analysis.

(**A**) Volcano plots indicating differentially regulated genes in MBD2 versus WT infected mouse brains. Adjusted p-values (padj) were calculated using the Benjamini-Hochberg procedure to control the false discovery rate (FDR) during differential expression analysis with DESeq2.
(**B**) Main enriched Gene Ontology (GO) terms of upregulated and downregulated genes in MBD2 versus WT infected mouse brains. P-values were calculated based on the cumulative hypergeometric distribution, and adjusted p-values were calculated using the Benjamini-Hochberg procedure to account for multiple tests.
(**C** and **D**) Heatmap analyses of the inflammatory response (C) and response to IFN-β genes (D) that were upregulated commonly upregulated in the WT and MBD2 infection groups compared to the Mock control.
(**E**) Heatmap analysis of the brain development-related genes that were downregulated in WT infected mice compared to Mock but upregulated in MBD2 infected mice compared to WT.

Appendix Fig. S3

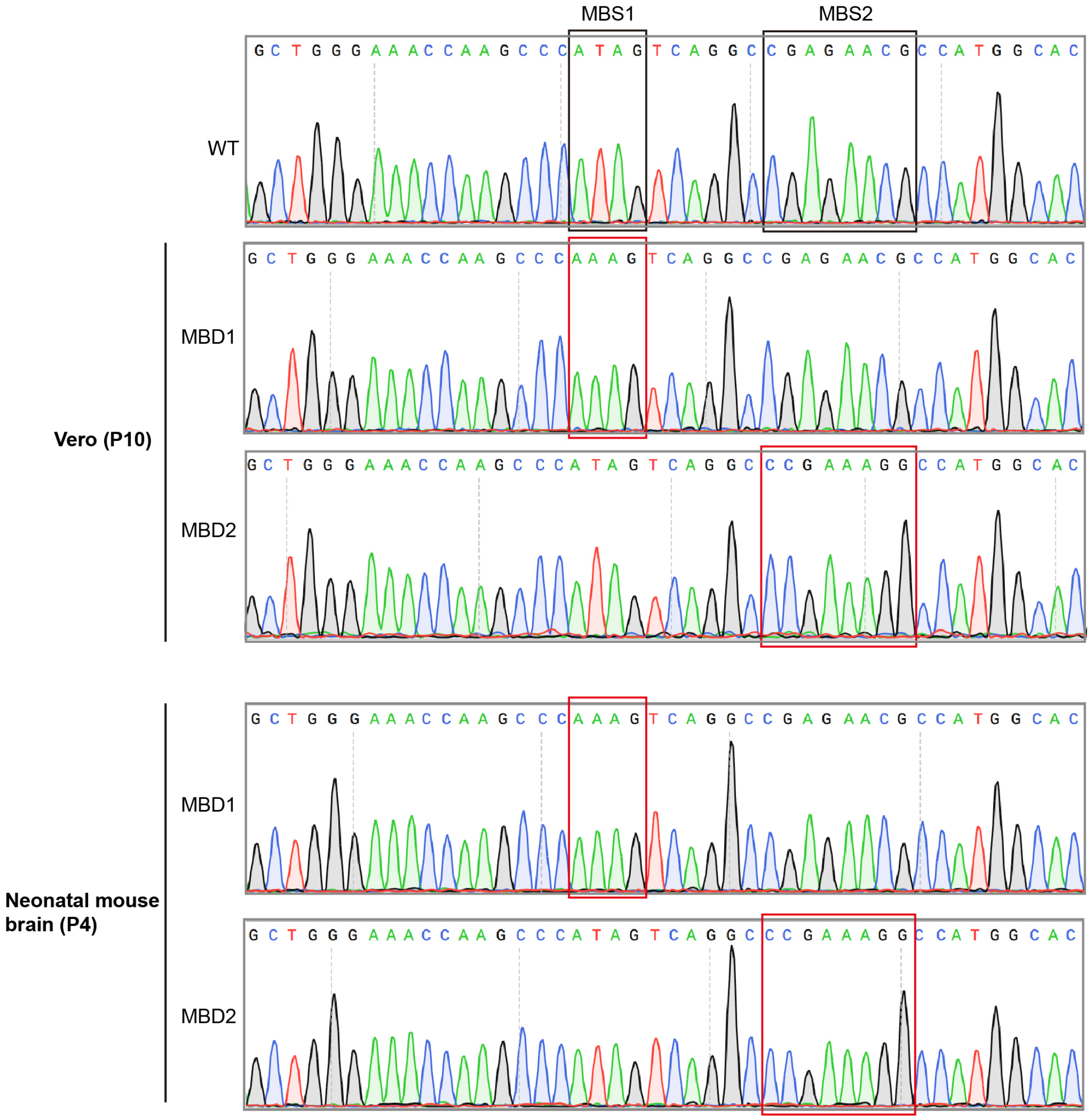

Appendix Fig. S3. Stability analysis of the MBD1 and MBD2 in vitro and in vivo. Sequence alignments of MBS1 and MBS2 in the 10th-passage (P10) viruses in Vero cells and the 4th-passage (P4) viruses in the brains of one-day-old neonatal mice.

Appendix Fig. S4

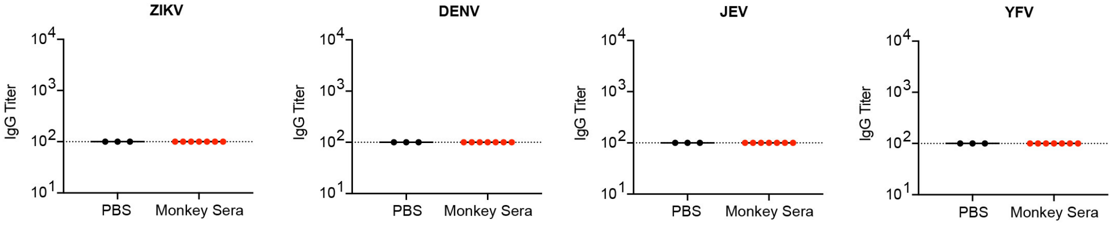

Appendix Fig. S4. Monkeys used in this study were negative for flavivirus before vaccination. The sera of 7 monkeys used in this study were tested for flaviviruses (ZIKV, DENV, JEV, and YFV) IgG by ELISA assay before vaccination. PBS was used as negative control. The dashed line indicates the LOD of the assay.
