# Supplementary figures and images for: Manipulating Zika virus RNA tertiary structure for developing tissue-specific attenuated vaccines
Source: EMBO Mol Med. 2025 Sep 8;17(10):2787–808. doi: 10.1038/s44321-025-00304-5 (PMC12514043; doi:10.1038/s44321-025-00304-5)

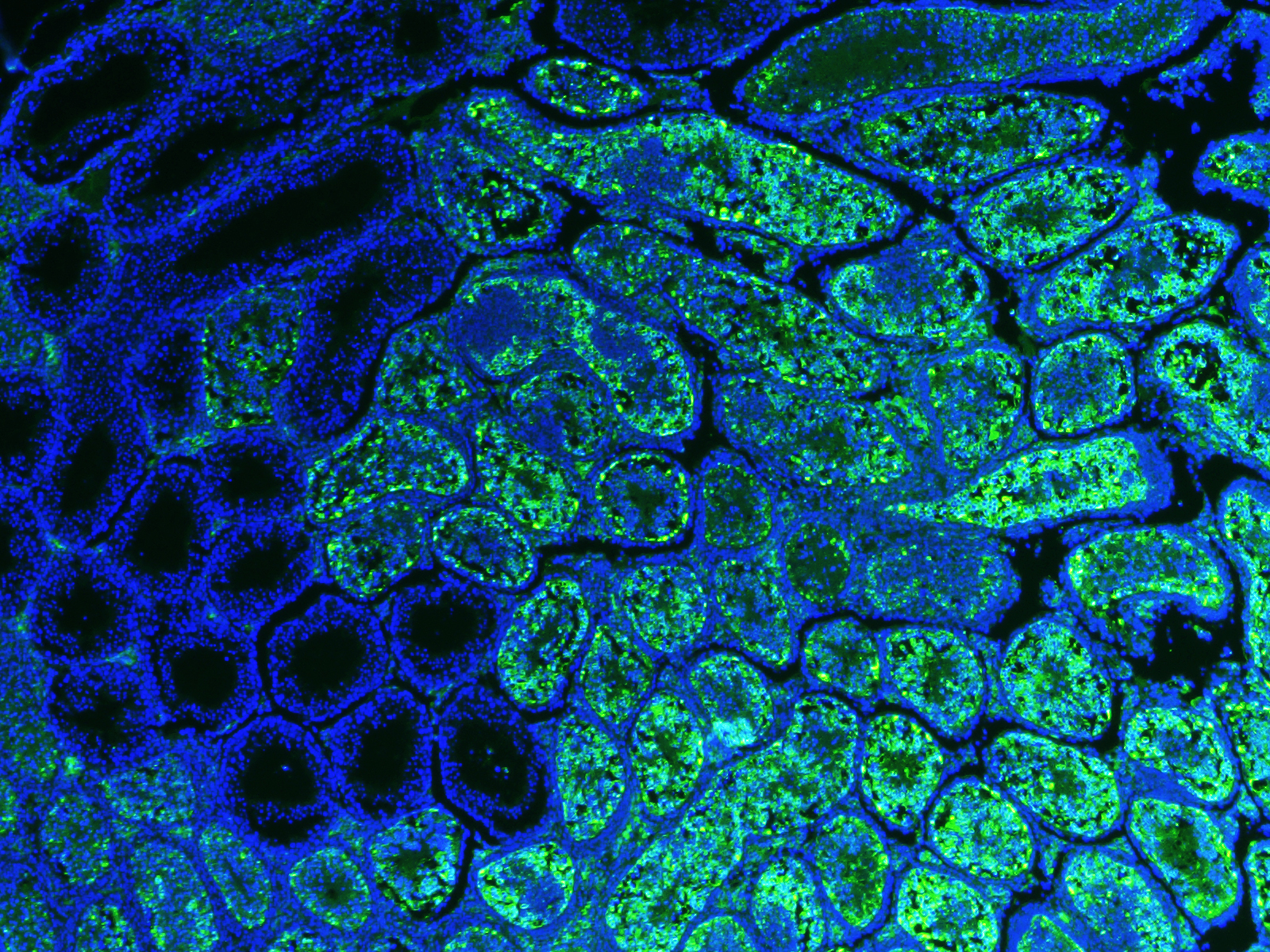

Supplement: Supplementary file 3 — Source data Fig. 1 [file 44321_2025_304_MOESM3_ESM.zip › Fig 1/Fig 1I/WT.tif]

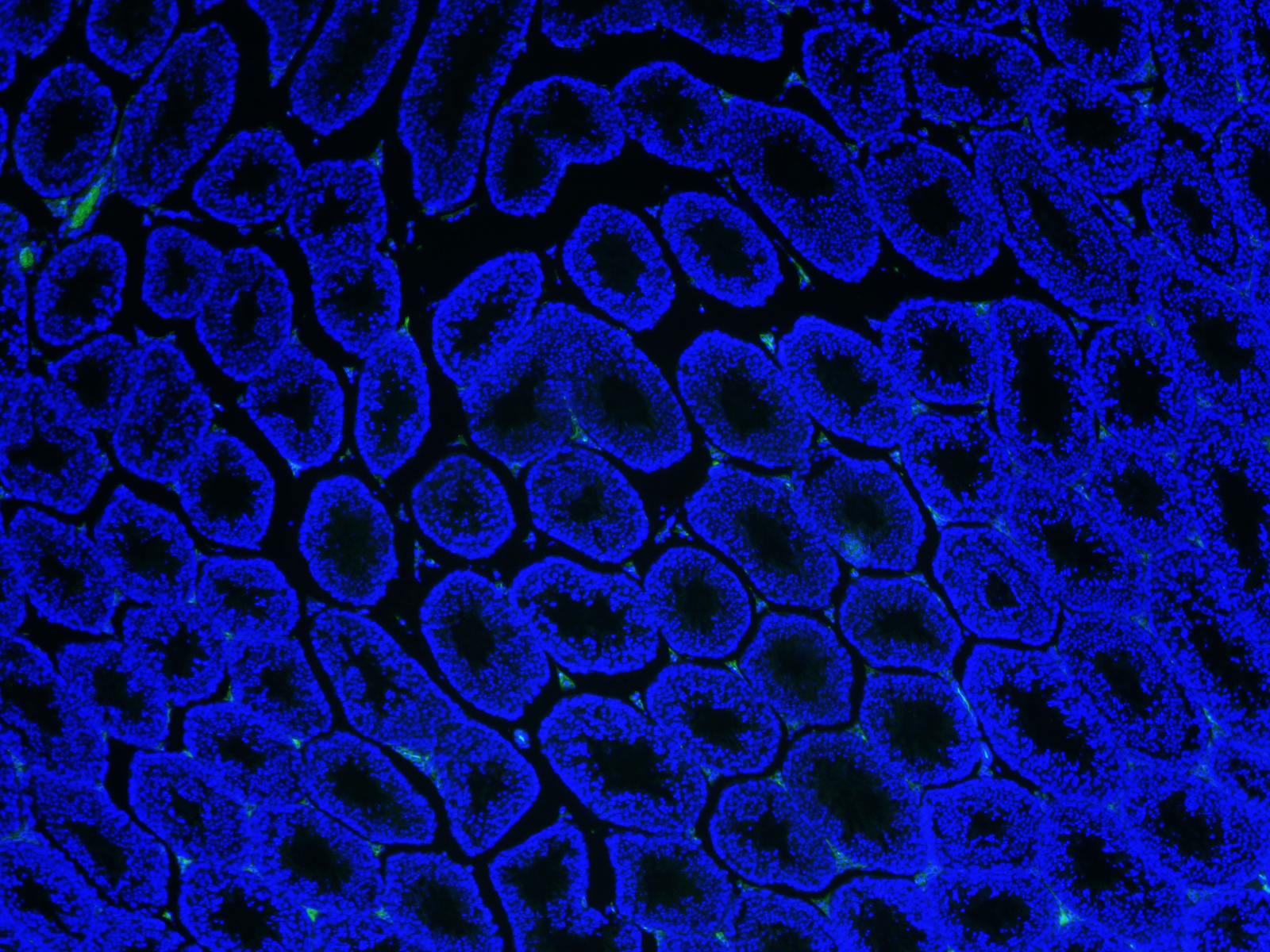

Supplement: Supplementary file 3 — Source data Fig. 1 [file 44321_2025_304_MOESM3_ESM.zip › Fig 1/Fig 1I/MOCK.tif]

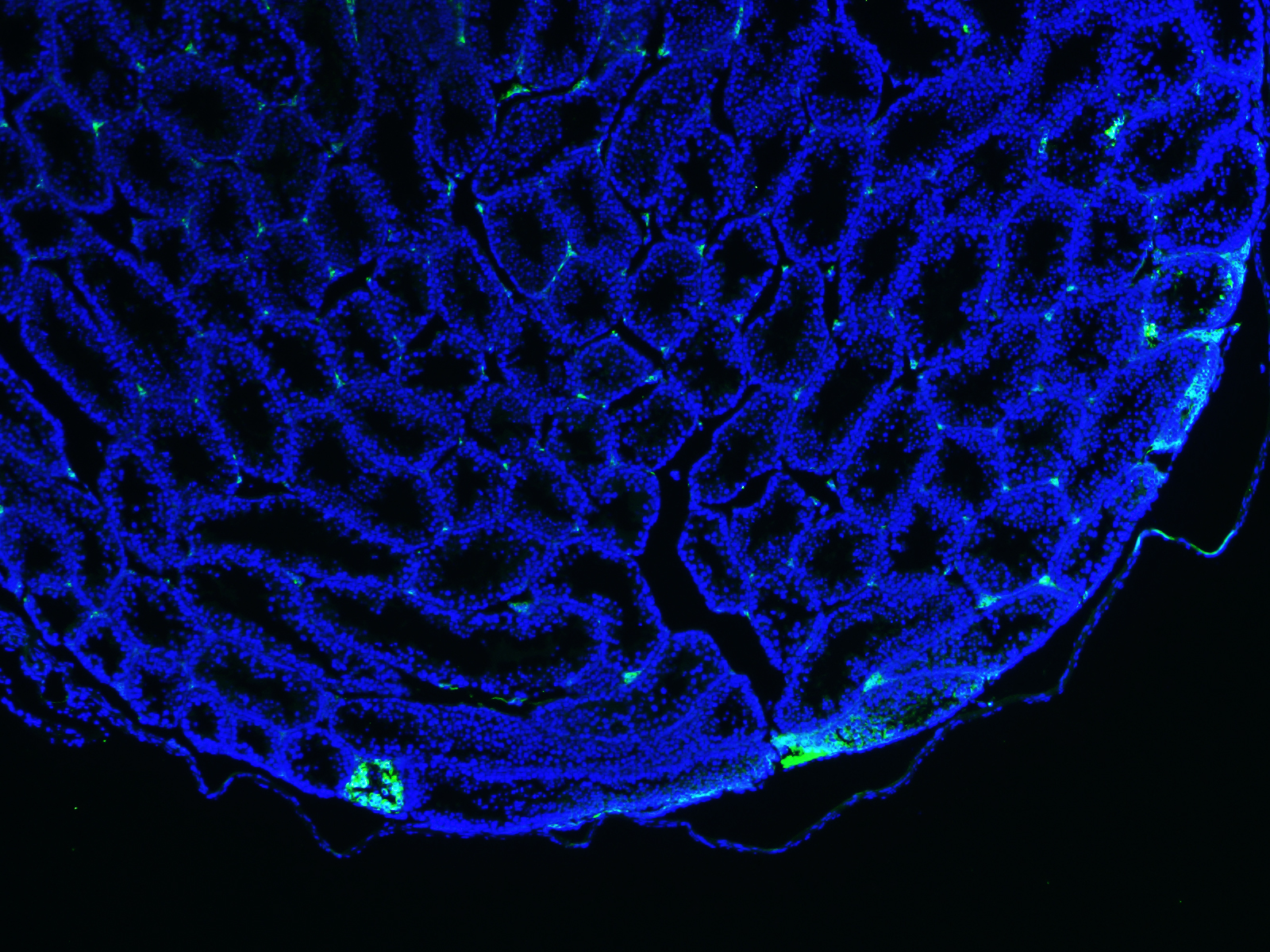

Supplement: Supplementary file 3 — Source data Fig. 1 [file 44321_2025_304_MOESM3_ESM.zip › Fig 1/Fig 1I/MBD2.tif]

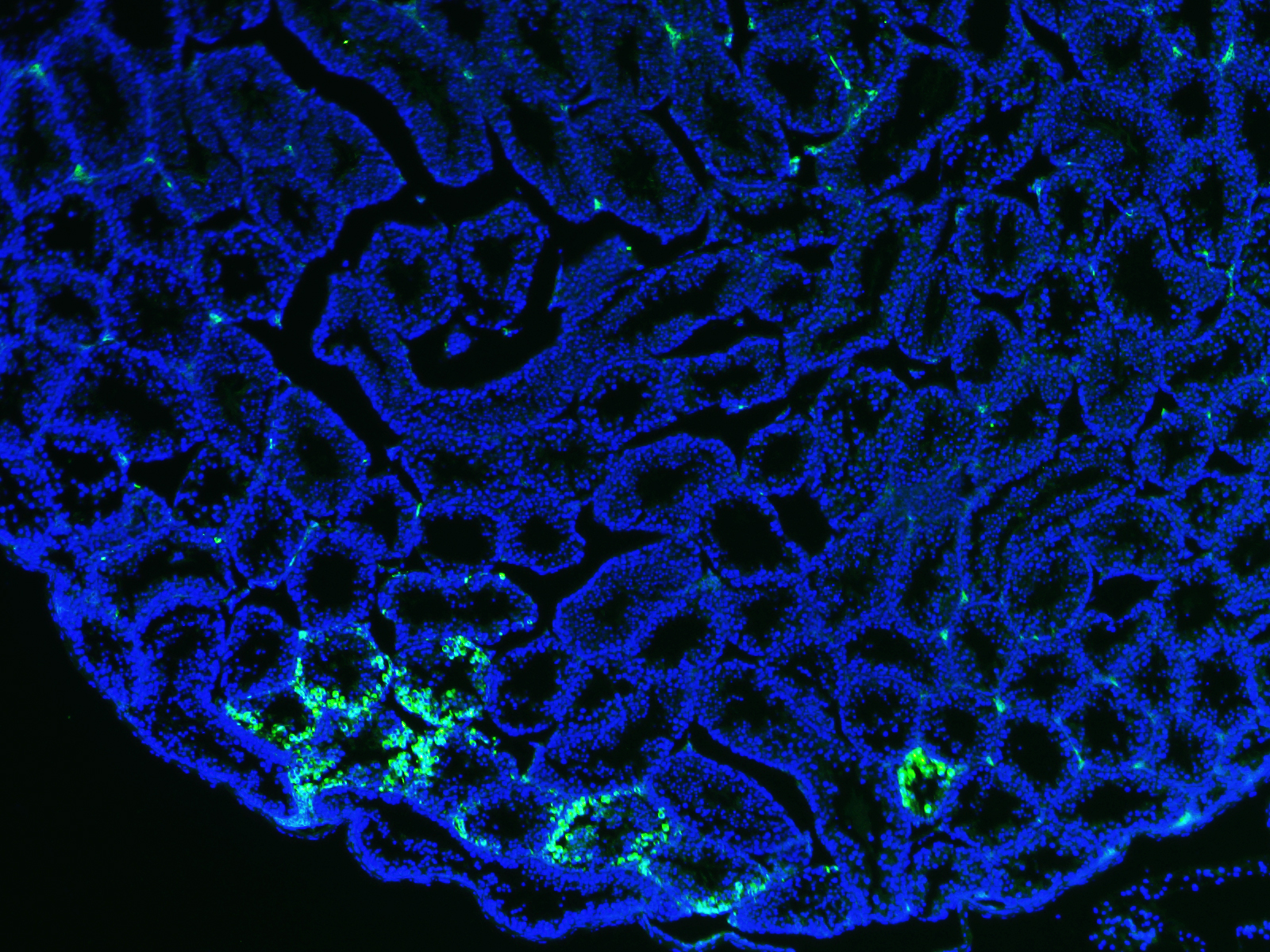

Supplement: Supplementary file 3 — Source data Fig. 1 [file 44321_2025_304_MOESM3_ESM.zip › Fig 1/Fig 1I/MBD1.tif]

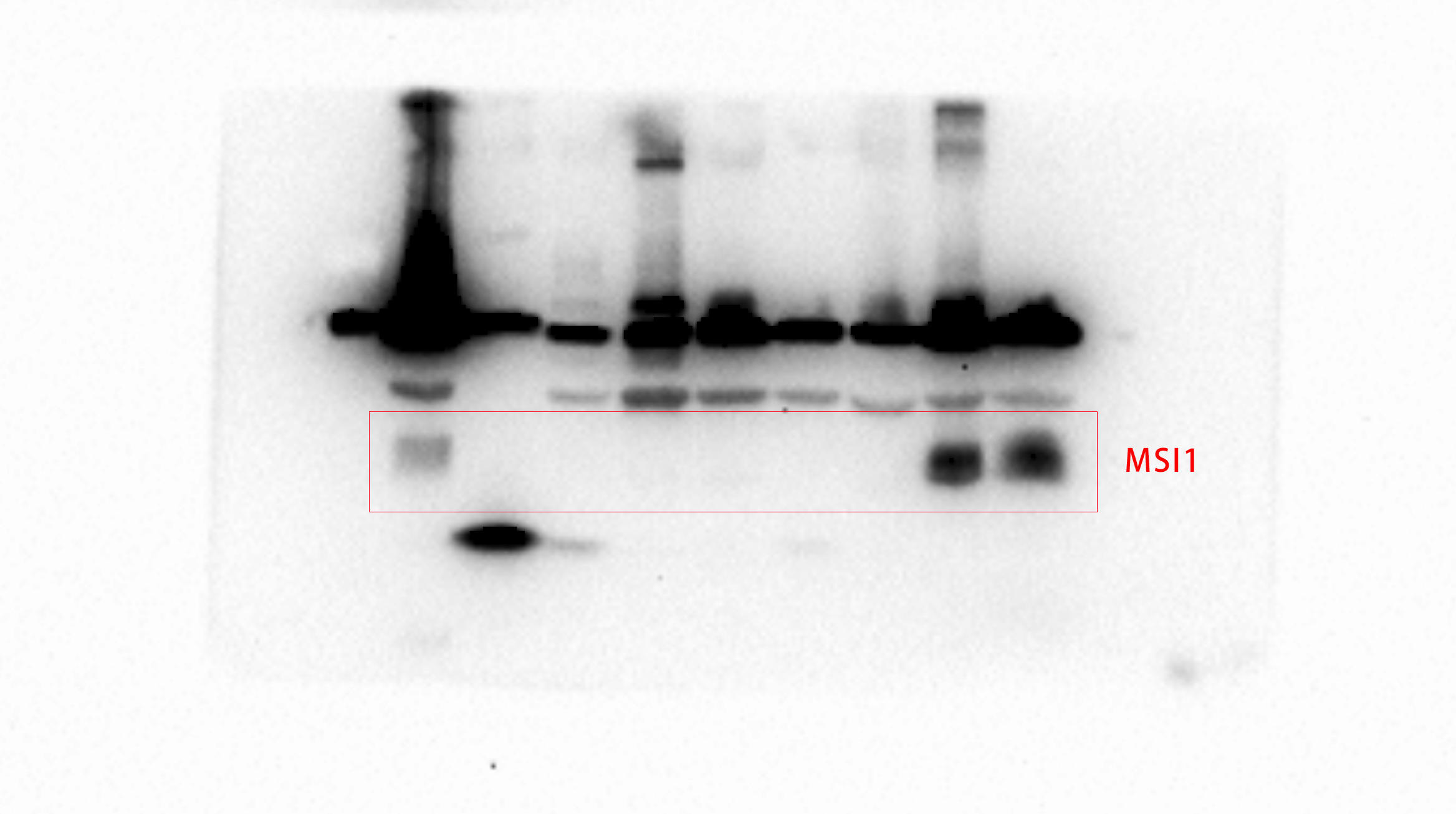

Supplement: Supplementary file 3 — Source data Fig. 1 [file 44321_2025_304_MOESM3_ESM.zip › Fig 1/Fig 1C/Western MSI1.tif]

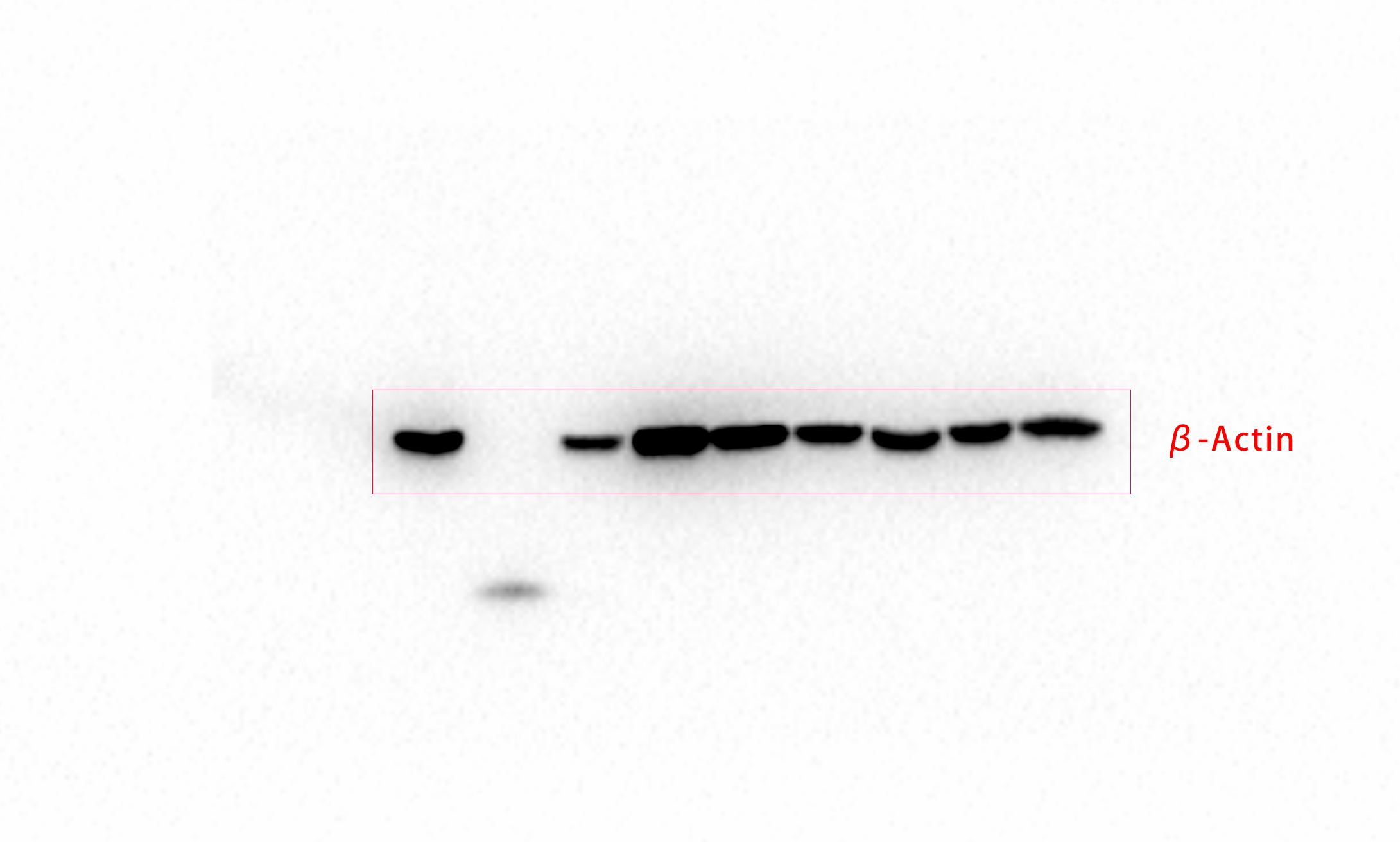

Supplement: Supplementary file 3 — Source data Fig. 1 [file 44321_2025_304_MOESM3_ESM.zip › Fig 1/Fig 1C/Western ╬▓-Actin.tif]

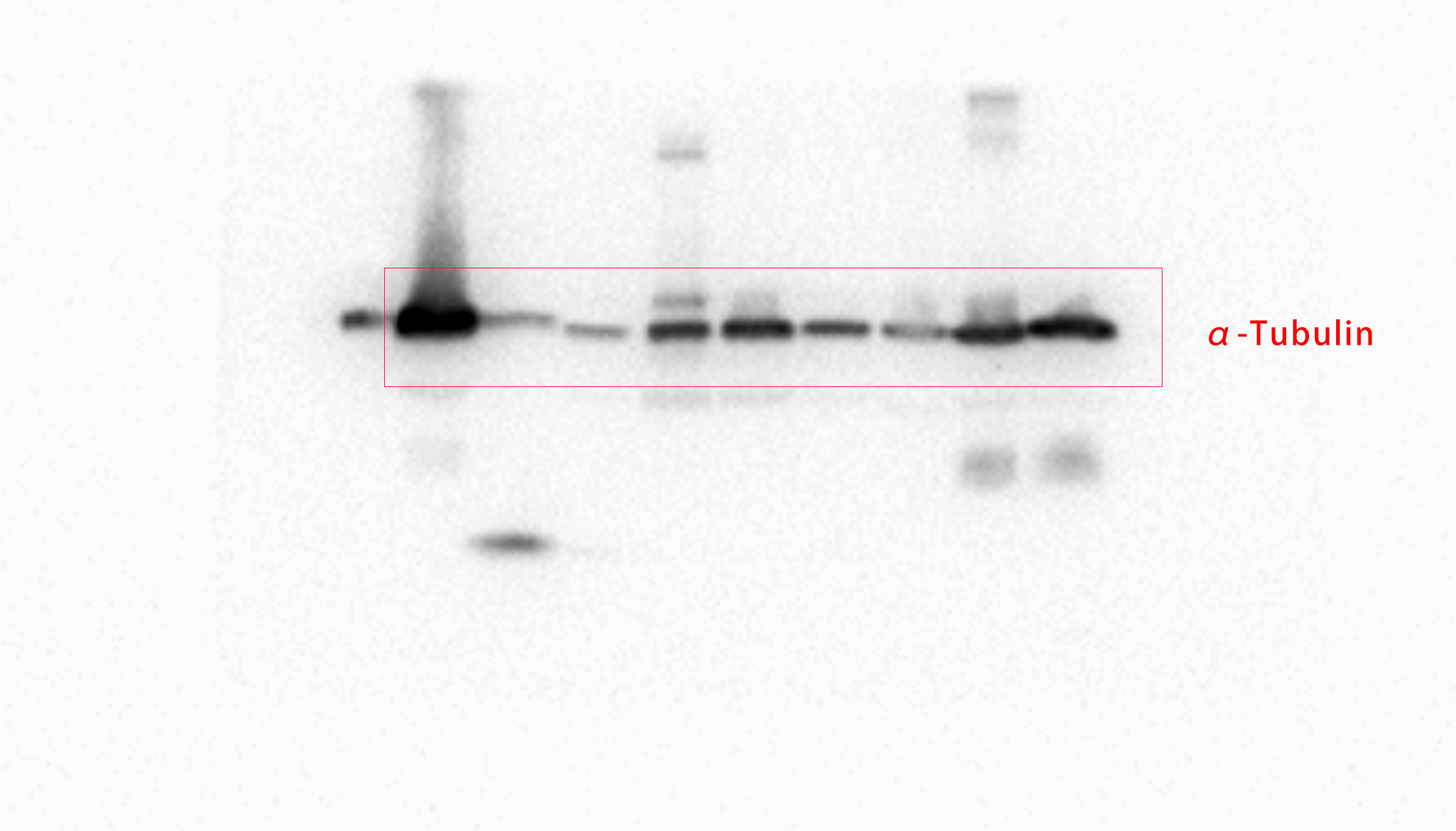

Supplement: Supplementary file 3 — Source data Fig. 1 [file 44321_2025_304_MOESM3_ESM.zip › Fig 1/Fig 1C/Western ╬▒-Tubulin.tif]

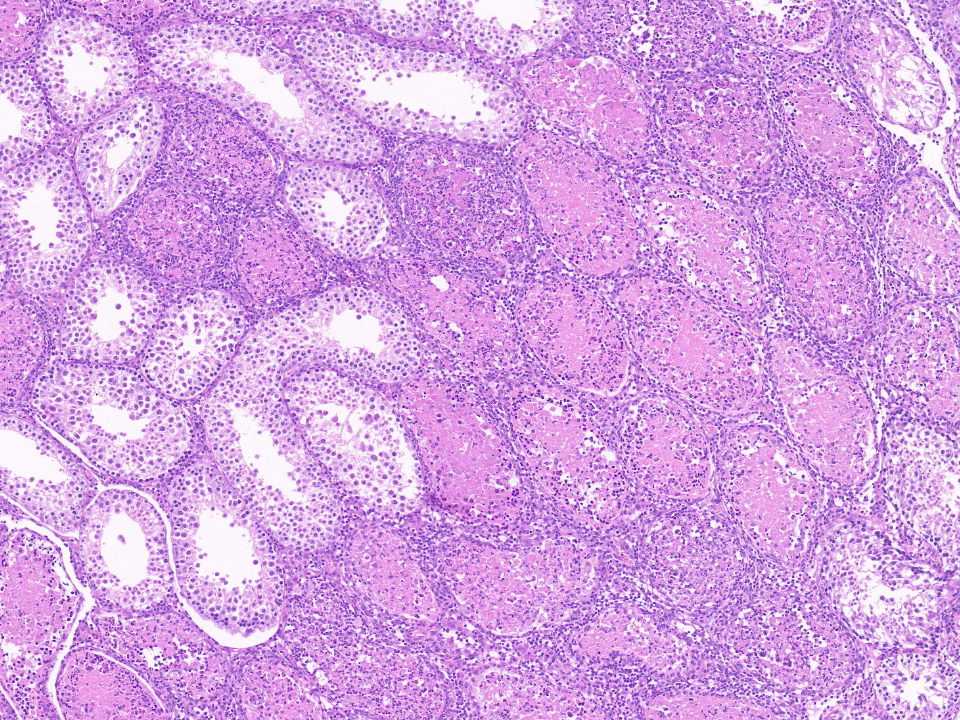

Supplement: Supplementary file 3 — Source data Fig. 1 [file 44321_2025_304_MOESM3_ESM.zip › Fig 1/Fig 1J/WT.tif]

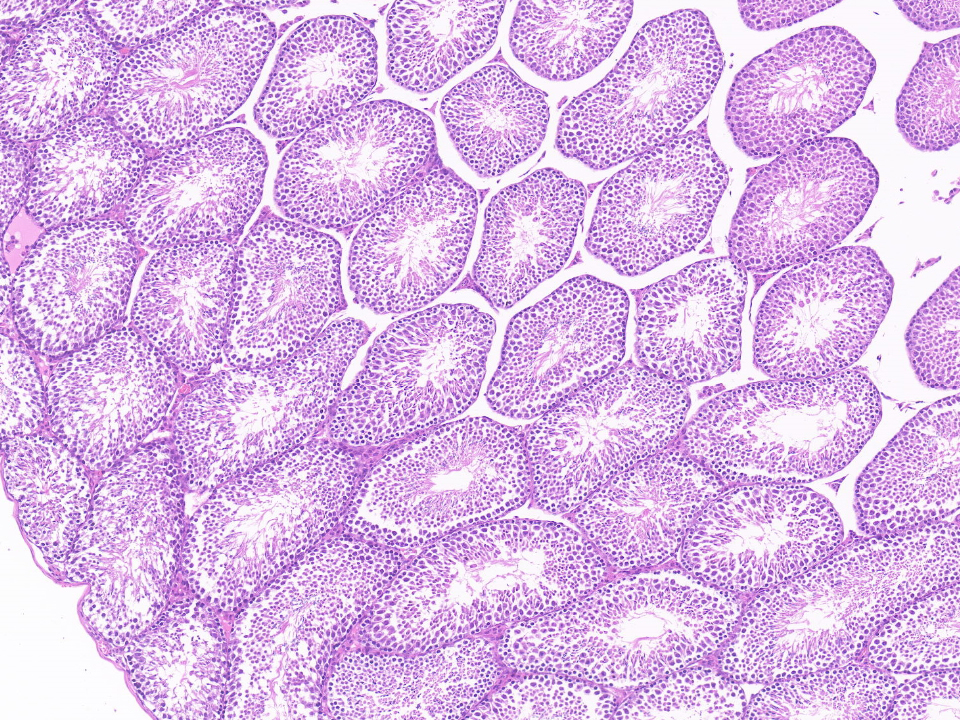

Supplement: Supplementary file 3 — Source data Fig. 1 [file 44321_2025_304_MOESM3_ESM.zip › Fig 1/Fig 1J/mock.tif]

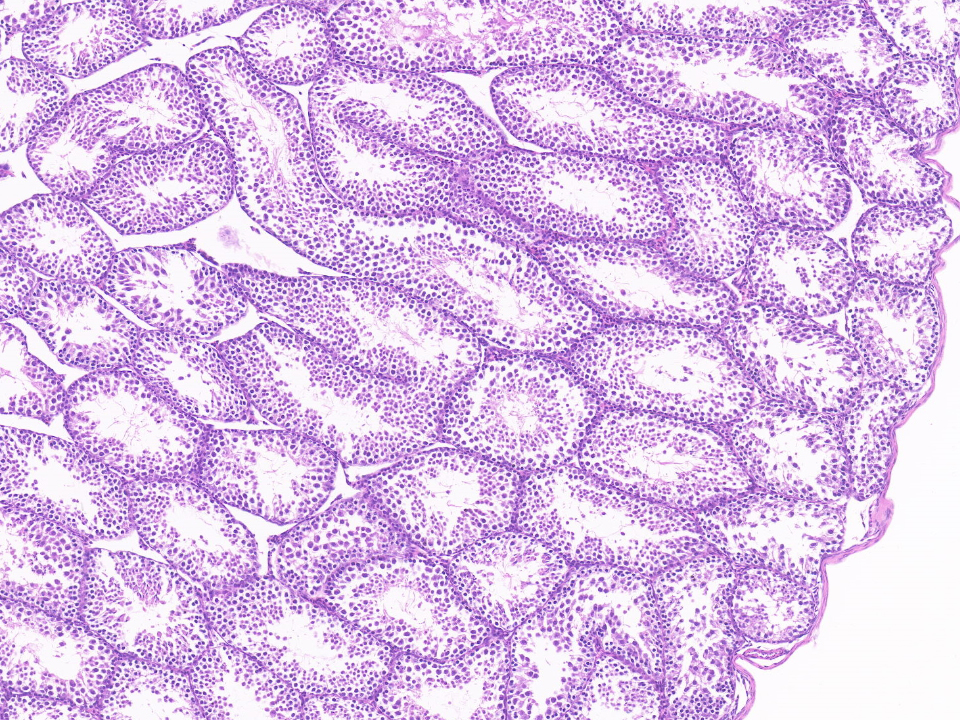

Supplement: Supplementary file 3 — Source data Fig. 1 [file 44321_2025_304_MOESM3_ESM.zip › Fig 1/Fig 1J/MBD2.tif]

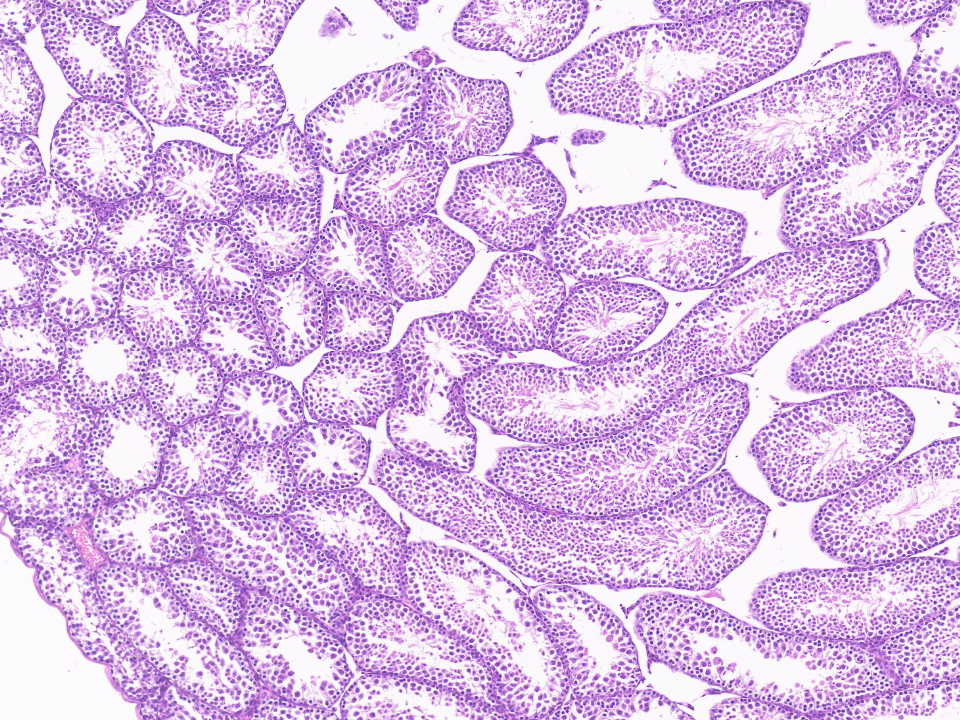

Supplement: Supplementary file 3 — Source data Fig. 1 [file 44321_2025_304_MOESM3_ESM.zip › Fig 1/Fig 1J/MBD1.tif]

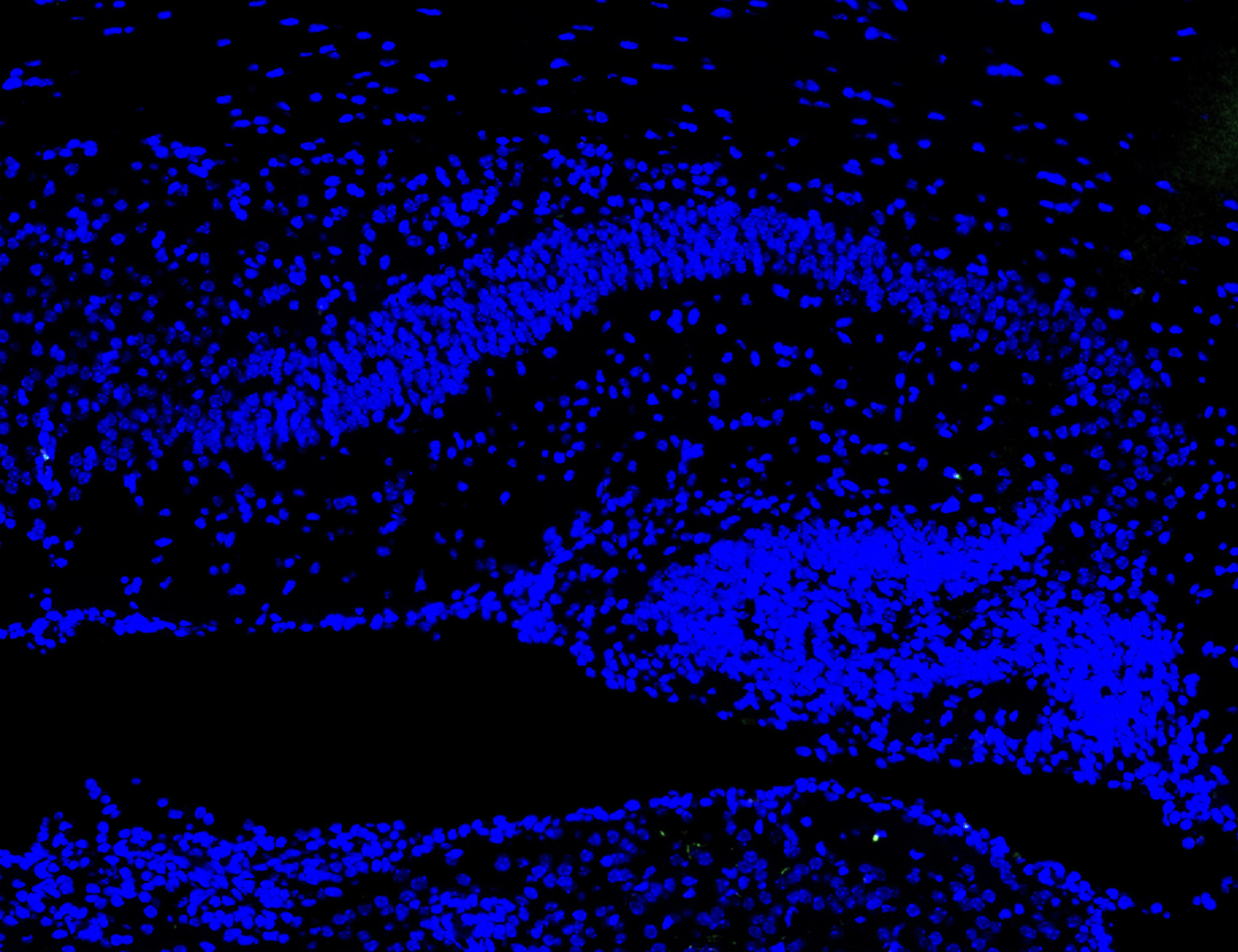

Supplement: Supplementary file 4 — Source data Fig. 2 [file 44321_2025_304_MOESM4_ESM.zip › Fig 2/Fig 2E/Mock d3jpg.tif]

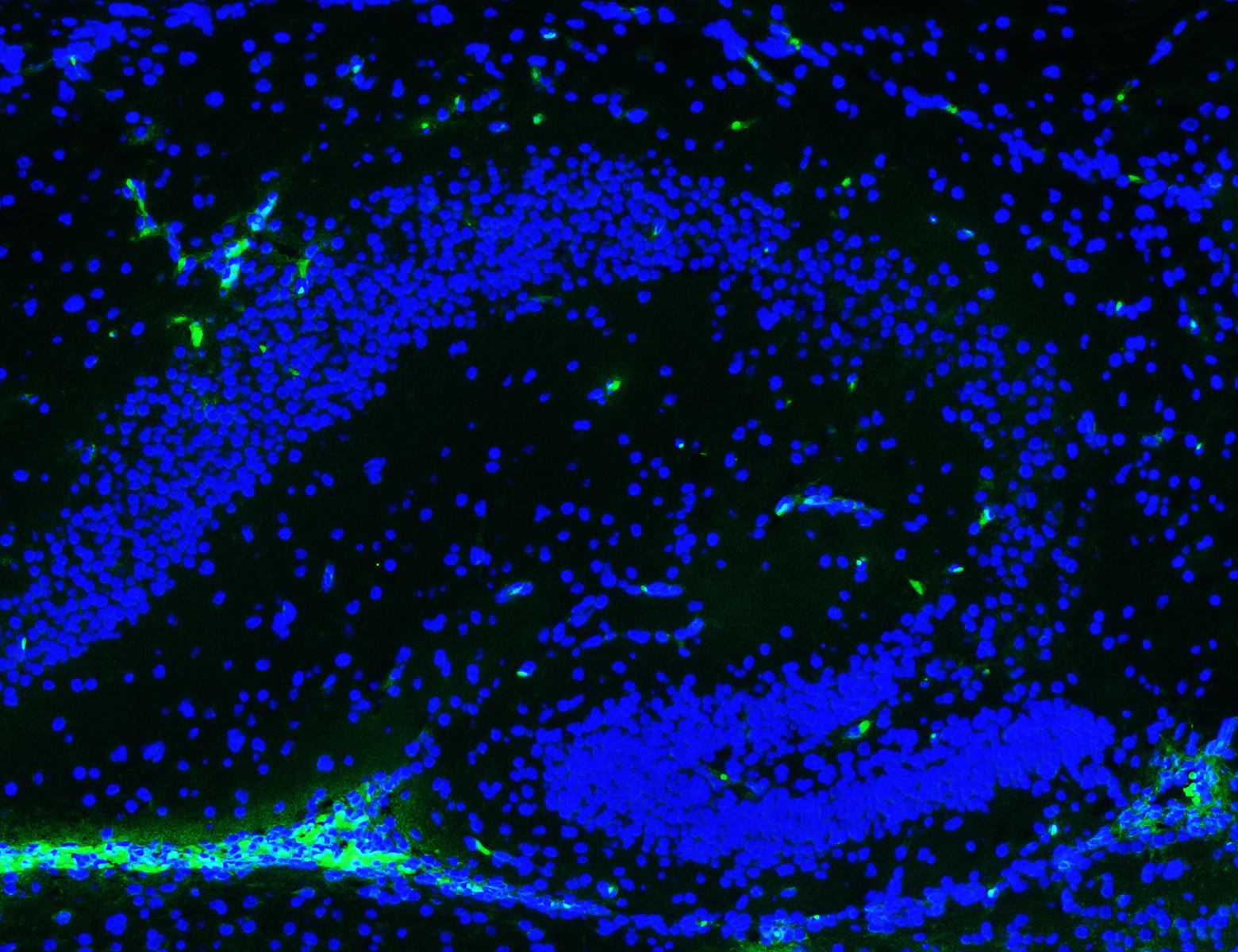

Supplement: Supplementary file 4 — Source data Fig. 2 [file 44321_2025_304_MOESM4_ESM.zip › Fig 2/Fig 2E/MBD2 d9.tif]

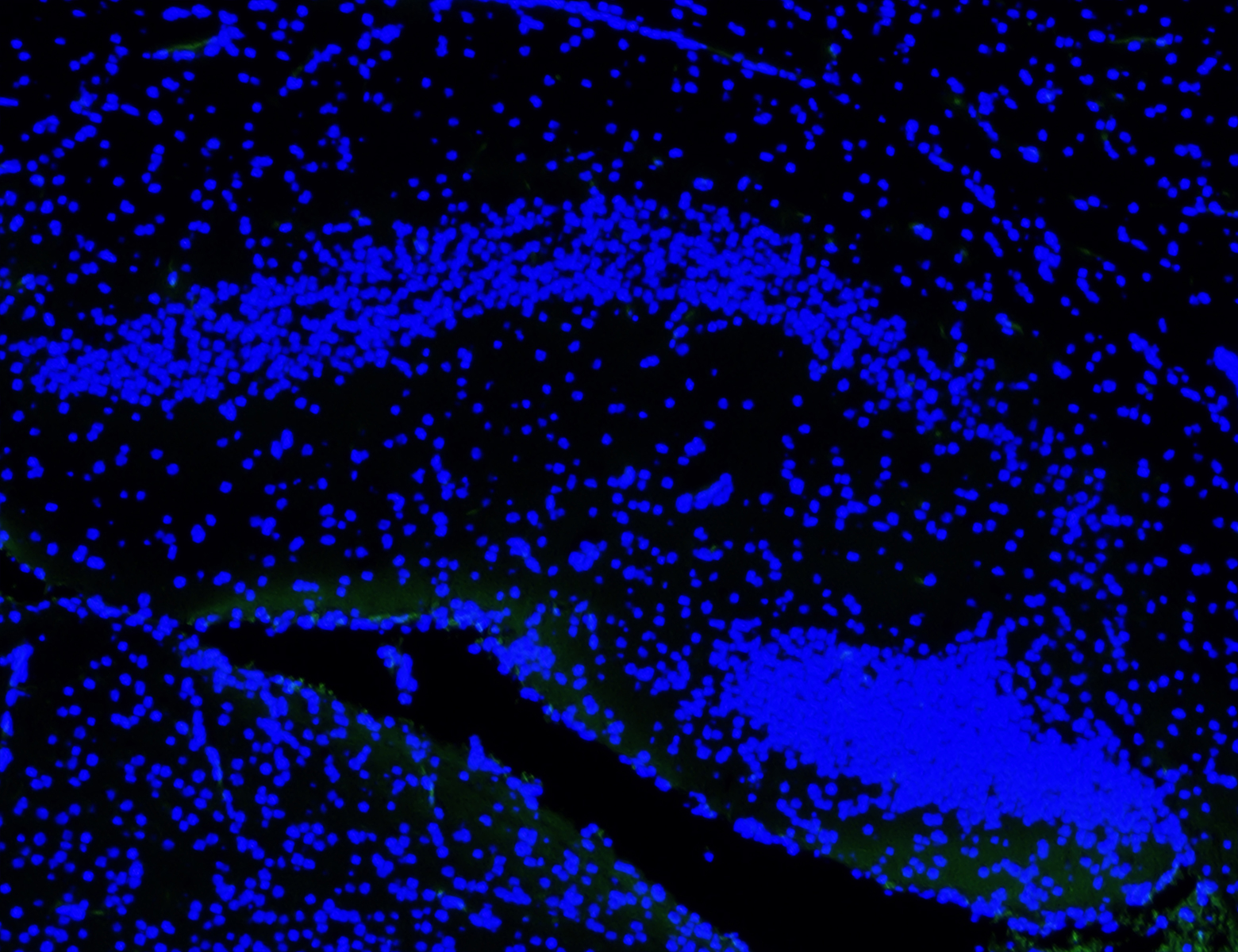

Supplement: Supplementary file 4 — Source data Fig. 2 [file 44321_2025_304_MOESM4_ESM.zip › Fig 2/Fig 2E/Mock d9.tif]

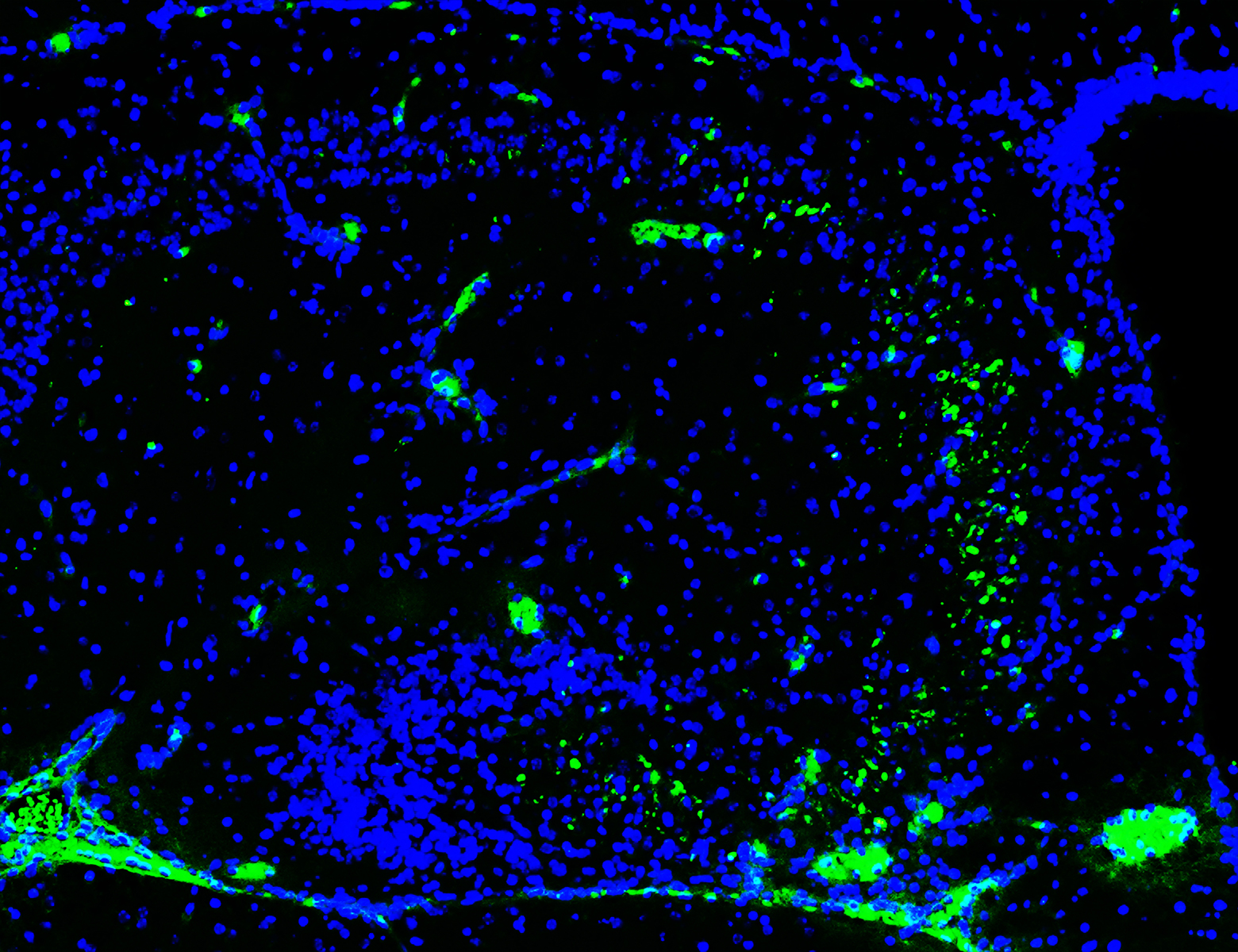

Supplement: Supplementary file 4 — Source data Fig. 2 [file 44321_2025_304_MOESM4_ESM.zip › Fig 2/Fig 2E/WT d9.tif]

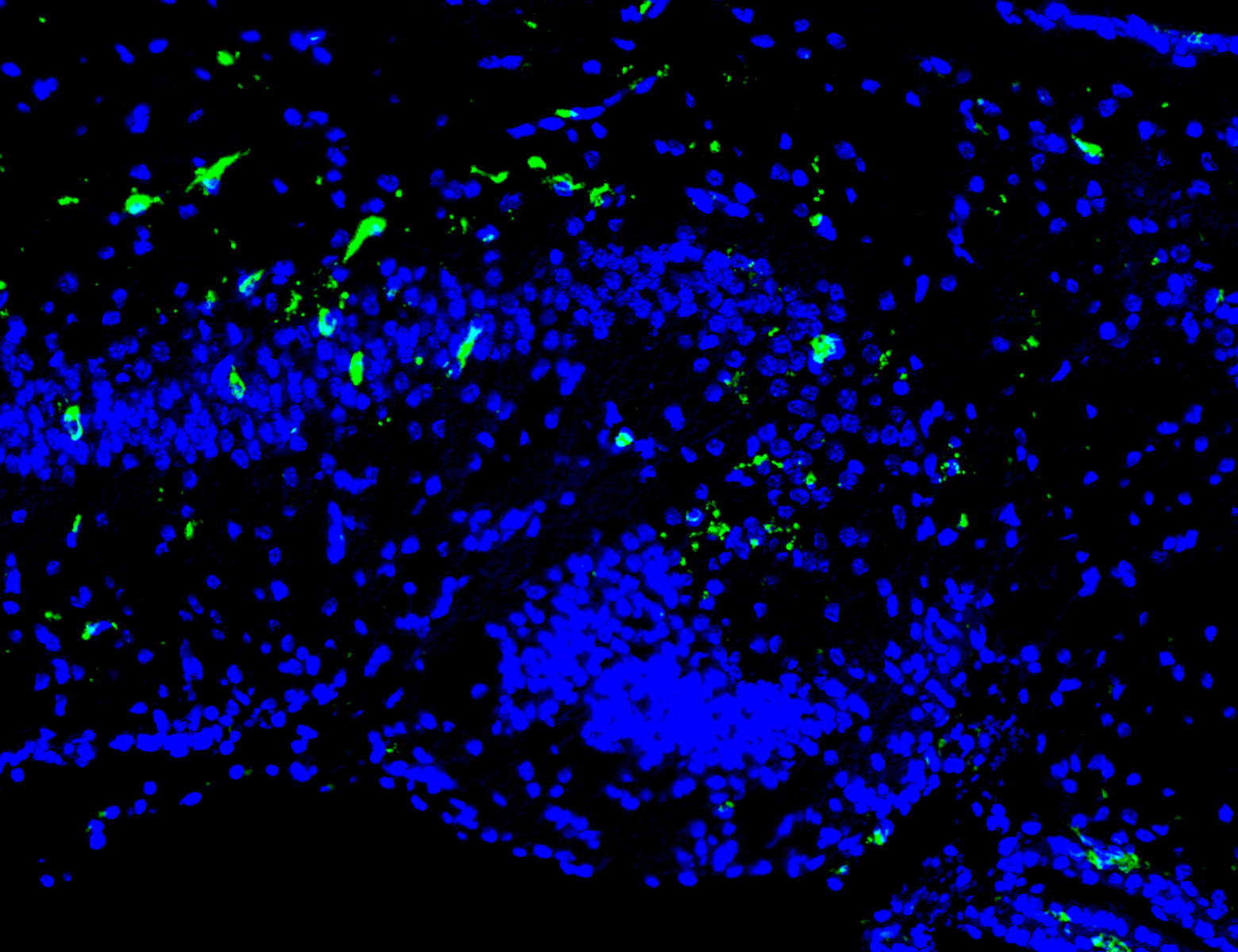

Supplement: Supplementary file 4 — Source data Fig. 2 [file 44321_2025_304_MOESM4_ESM.zip › Fig 2/Fig 2E/MBD1 d6.tif]

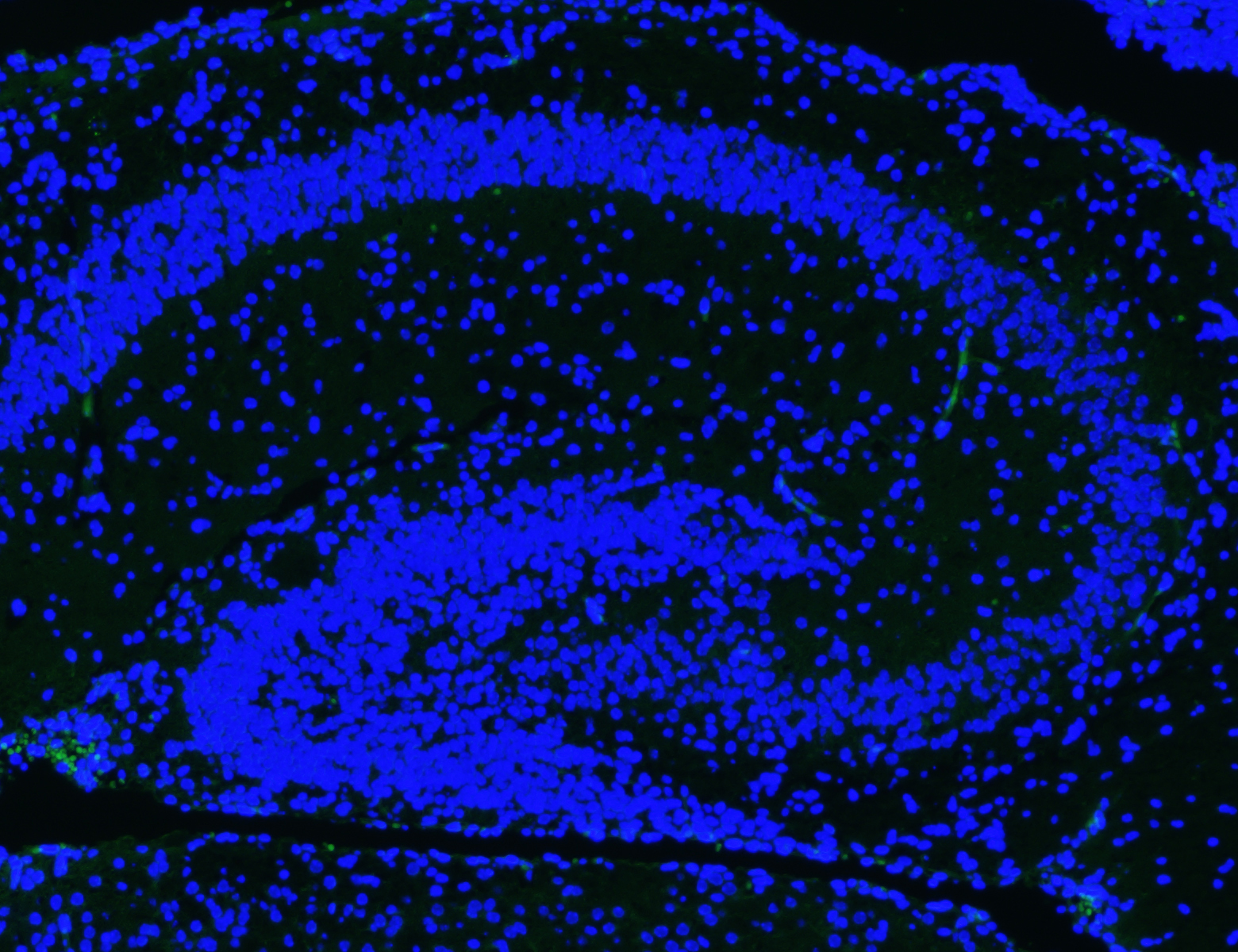

Supplement: Supplementary file 4 — Source data Fig. 2 [file 44321_2025_304_MOESM4_ESM.zip › Fig 2/Fig 2E/MBD1 d3.tif]

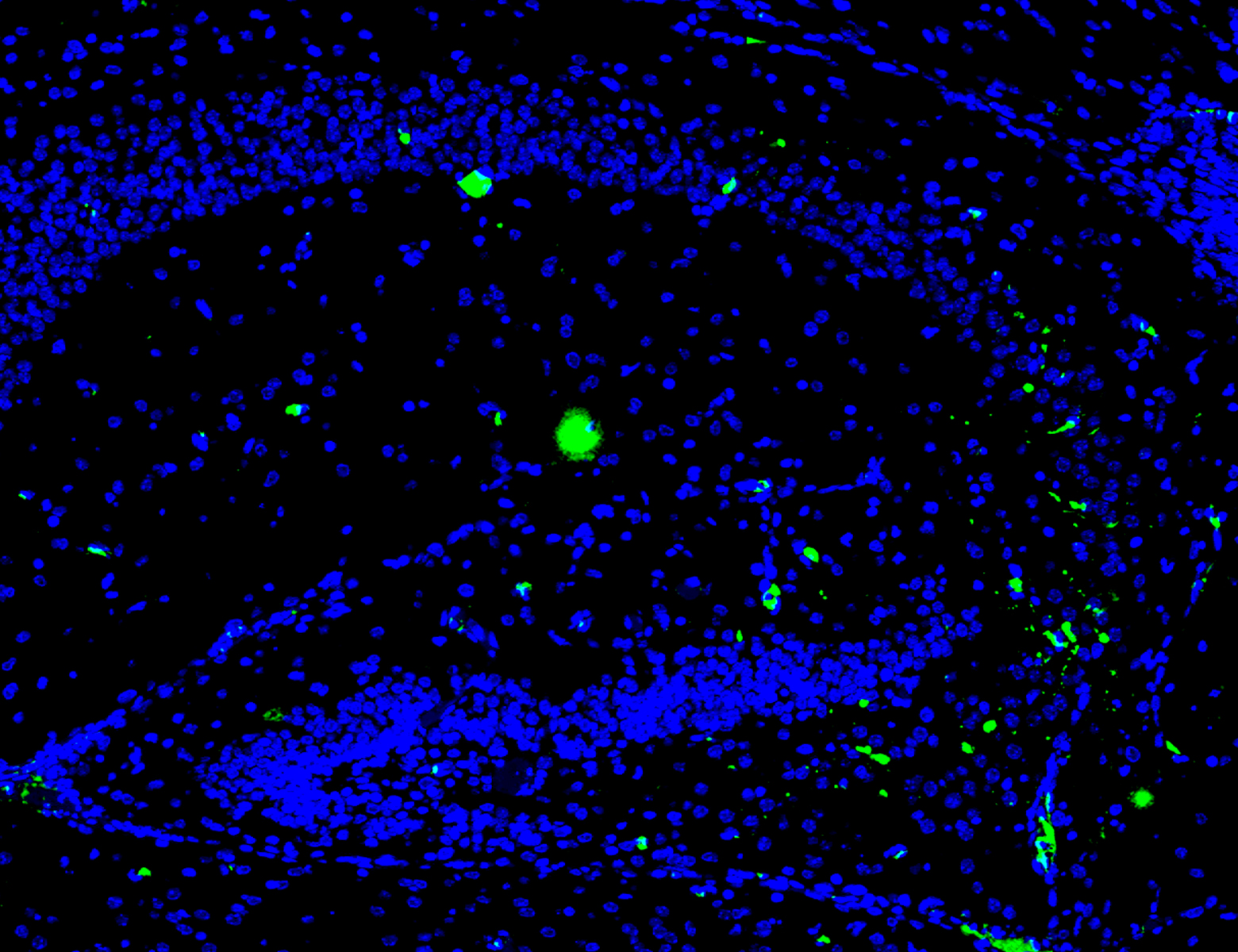

Supplement: Supplementary file 4 — Source data Fig. 2 [file 44321_2025_304_MOESM4_ESM.zip › Fig 2/Fig 2E/MBD1 d9.tif]

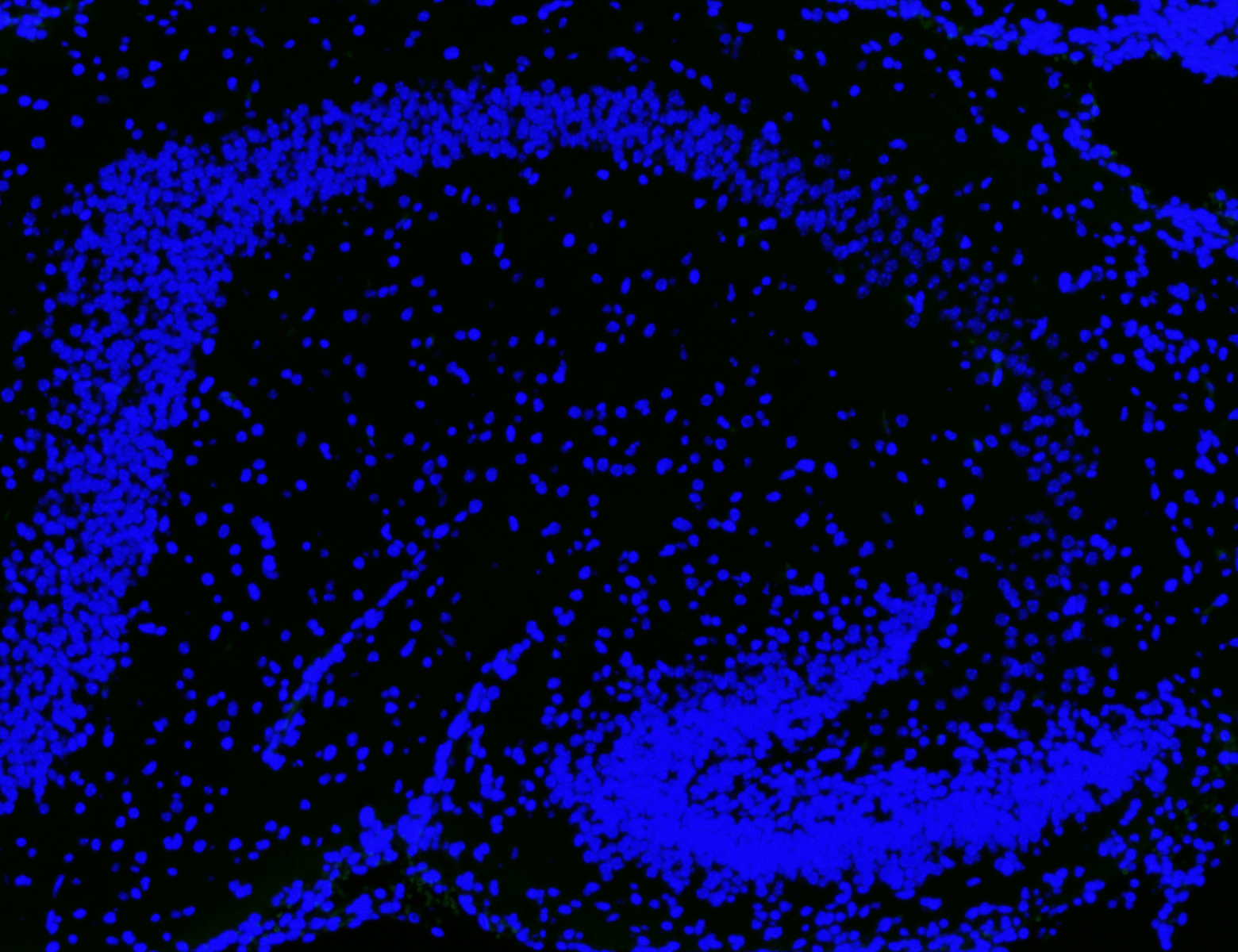

Supplement: Supplementary file 4 — Source data Fig. 2 [file 44321_2025_304_MOESM4_ESM.zip › Fig 2/Fig 2E/Mock d6.tif]

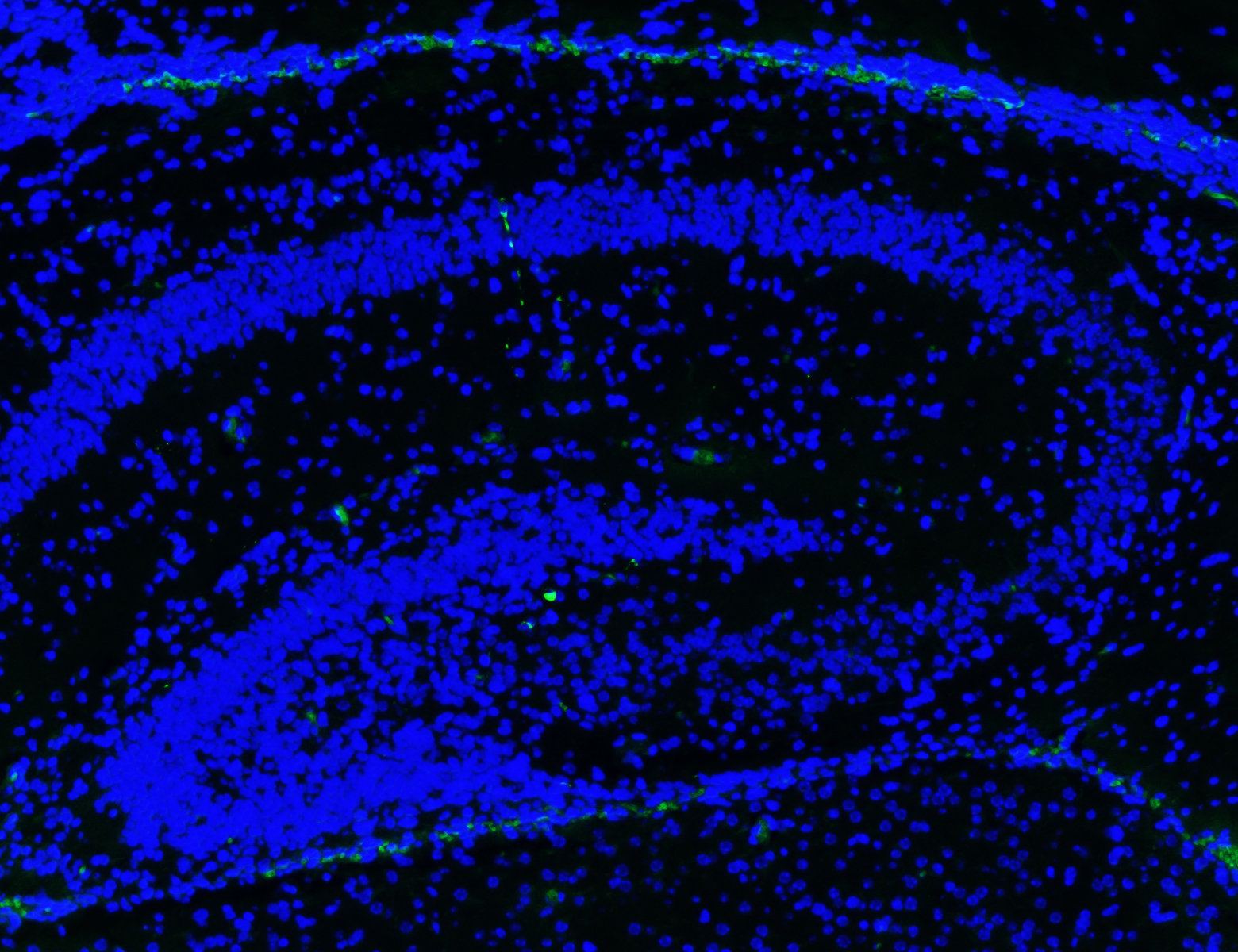

Supplement: Supplementary file 4 — Source data Fig. 2 [file 44321_2025_304_MOESM4_ESM.zip › Fig 2/Fig 2E/MBD2 d3.tif]

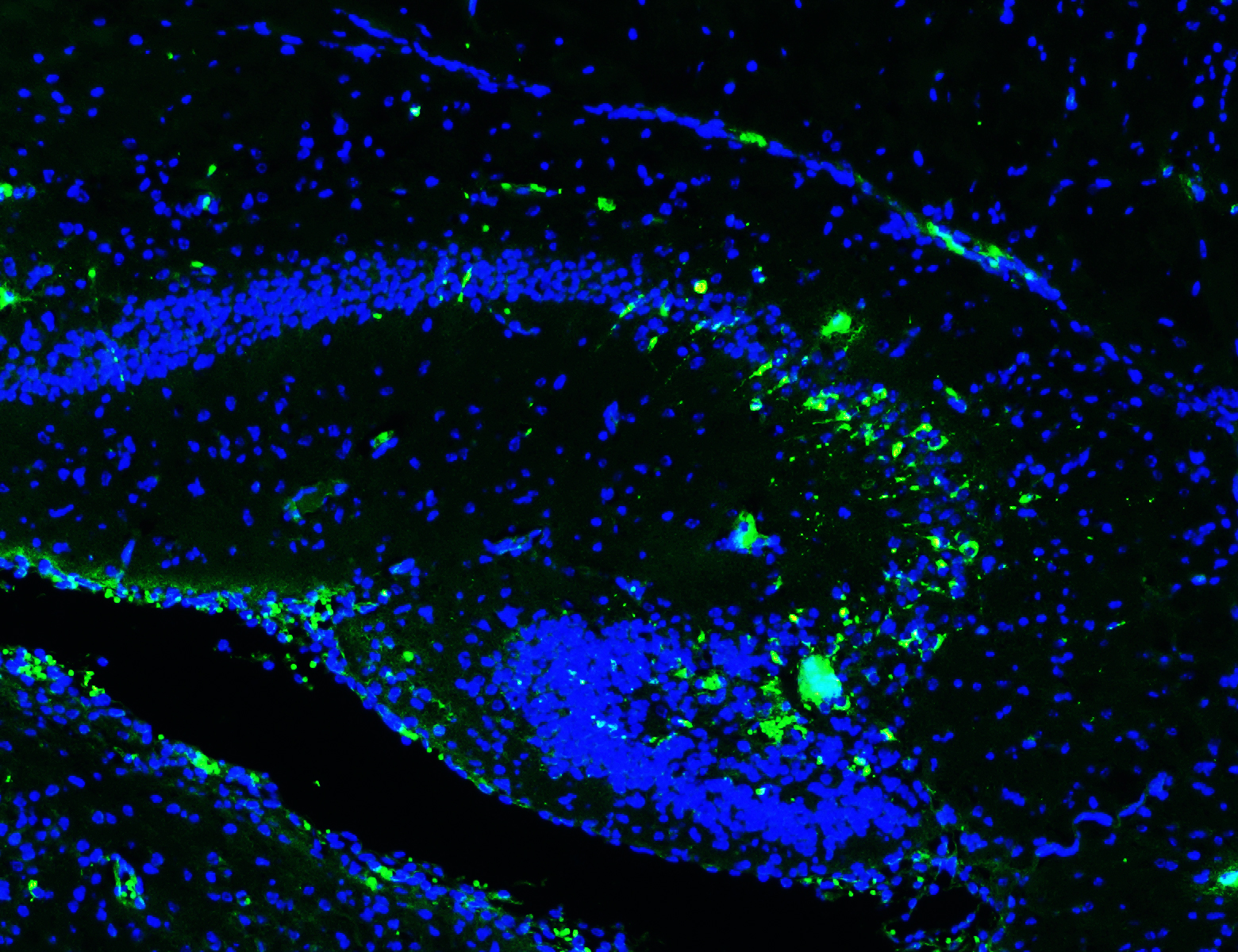

Supplement: Supplementary file 4 — Source data Fig. 2 [file 44321_2025_304_MOESM4_ESM.zip › Fig 2/Fig 2E/WT d6.tif]

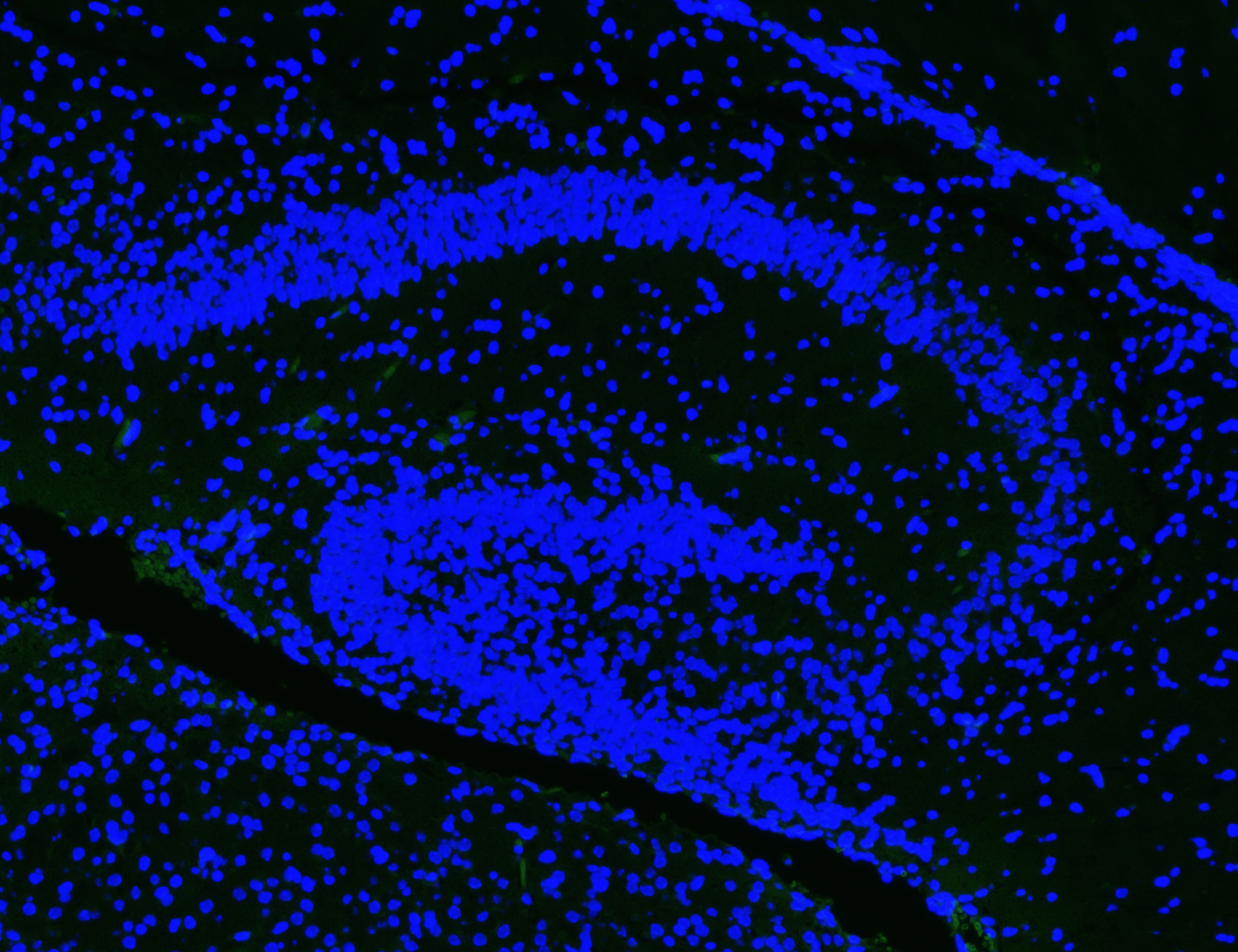

Supplement: Supplementary file 4 — Source data Fig. 2 [file 44321_2025_304_MOESM4_ESM.zip › Fig 2/Fig 2E/WT d3.tif]

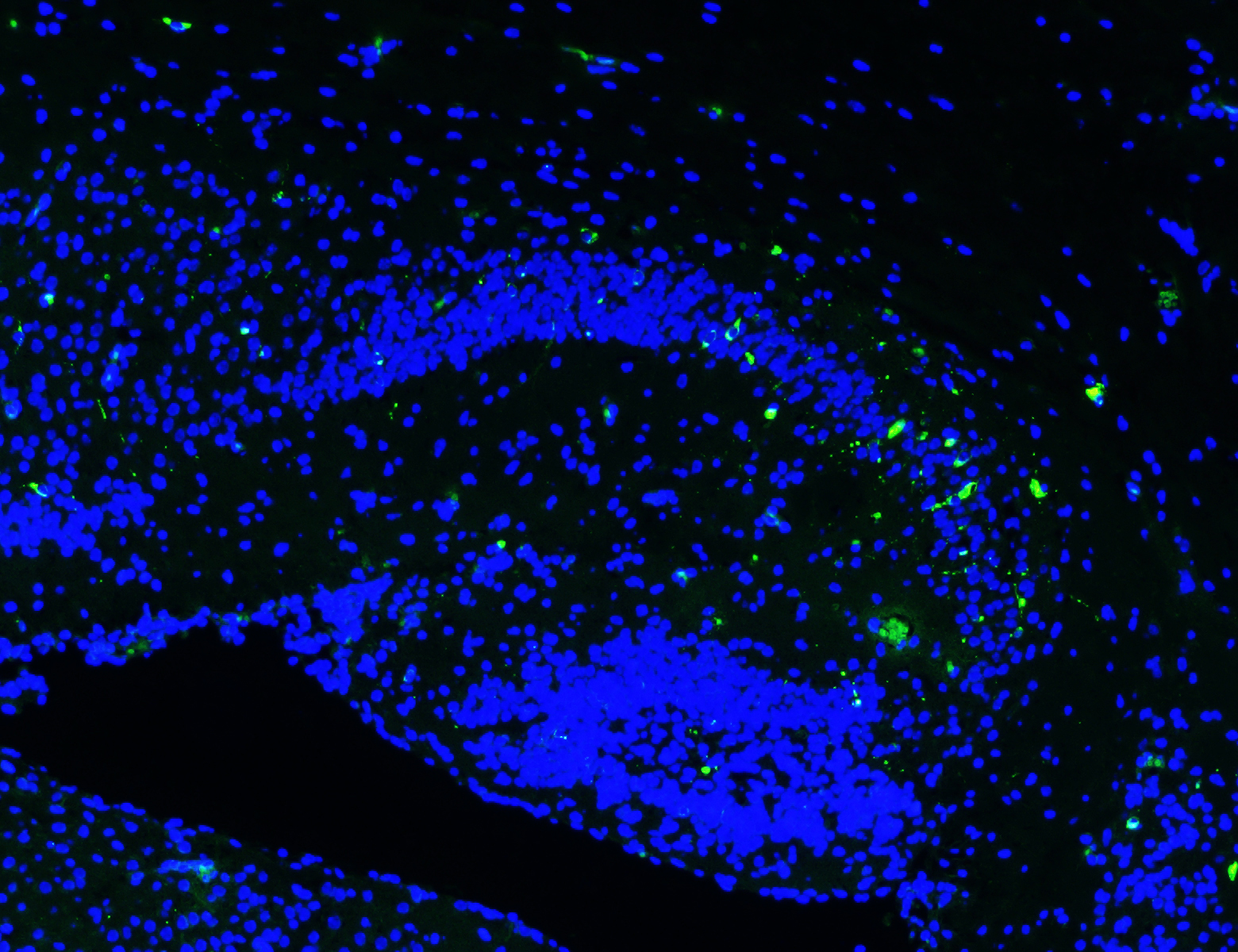

Supplement: Supplementary file 4 — Source data Fig. 2 [file 44321_2025_304_MOESM4_ESM.zip › Fig 2/Fig 2E/MBD2 d6.tif]

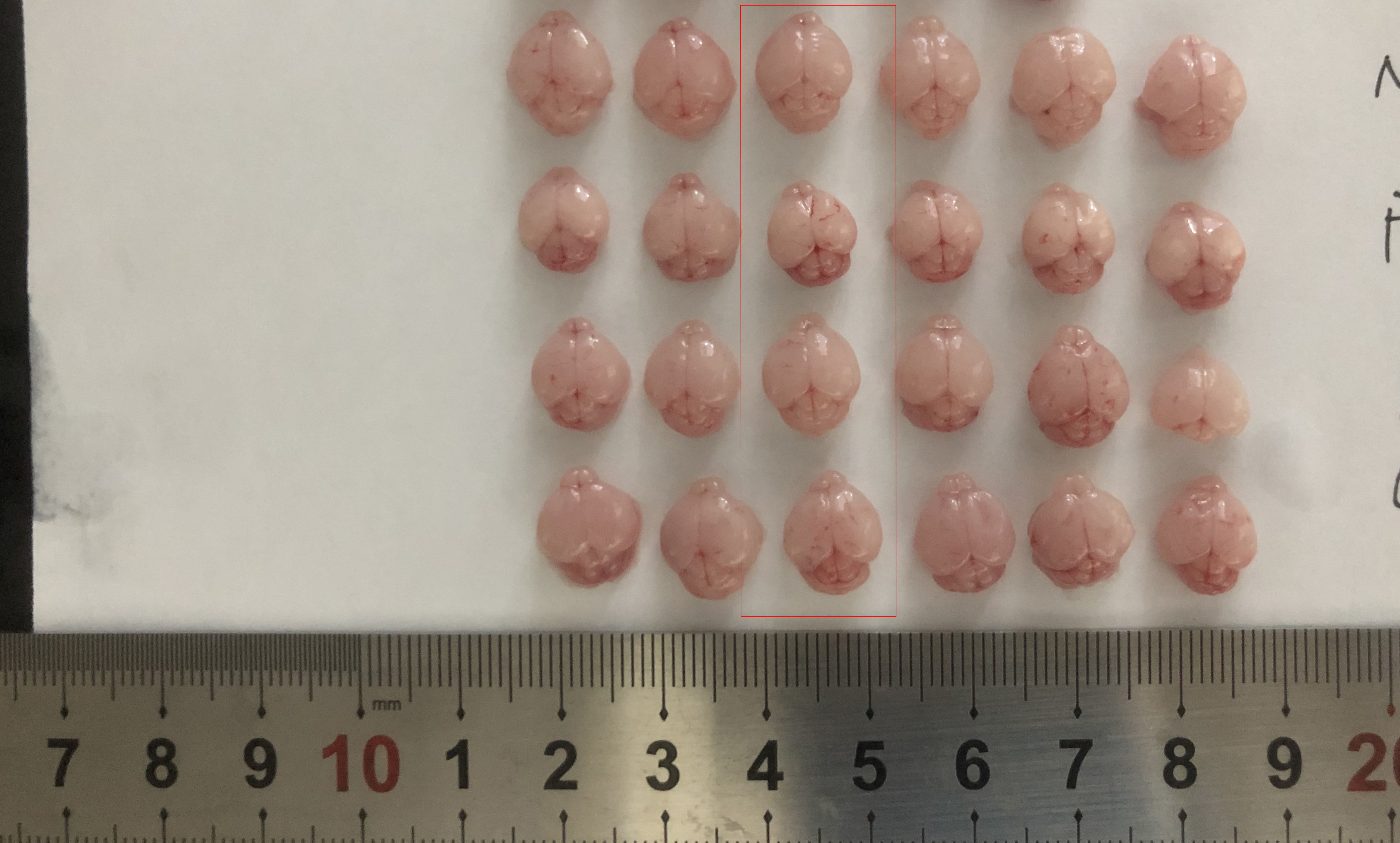

Supplement: Supplementary file 4 — Source data Fig. 2 [file 44321_2025_304_MOESM4_ESM.zip › Fig 2/Fig 2B/mouse brain picture.jpg]

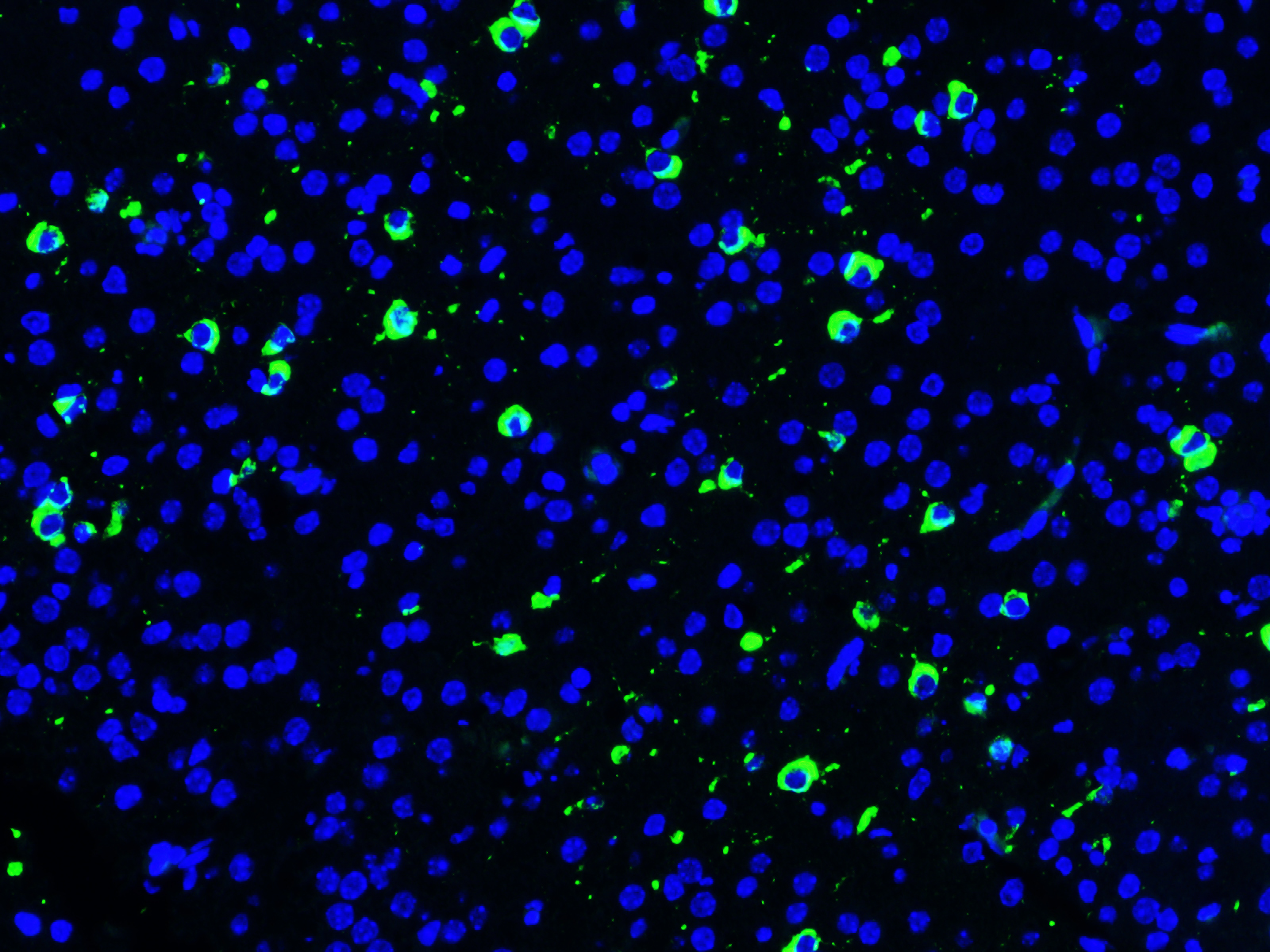

Supplement: Supplementary file 4 — Source data Fig. 2 [file 44321_2025_304_MOESM4_ESM.zip › Fig 2/Fig 2D/MBD2 d9.tif]

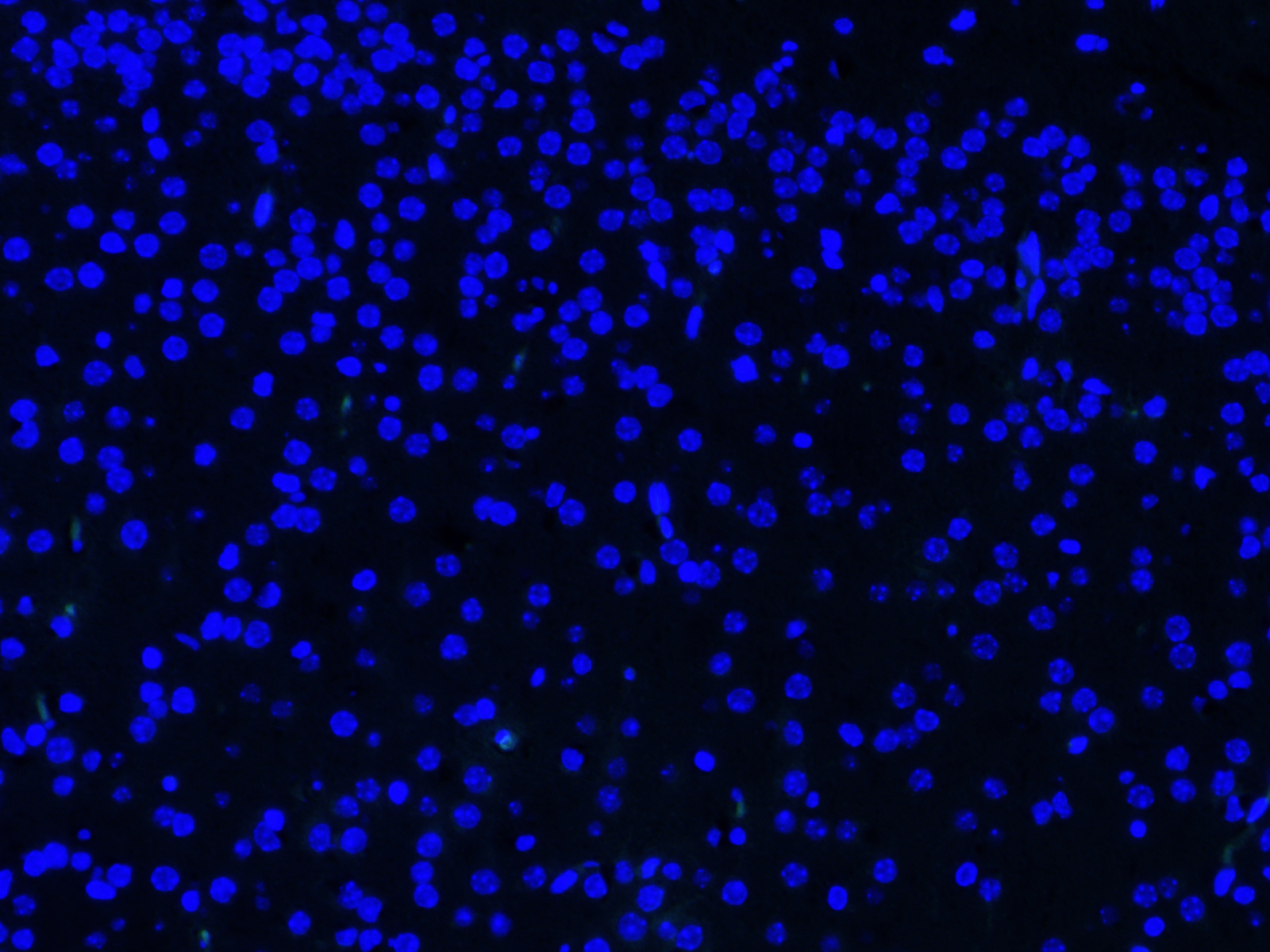

Supplement: Supplementary file 4 — Source data Fig. 2 [file 44321_2025_304_MOESM4_ESM.zip › Fig 2/Fig 2D/Mock d9.tif]

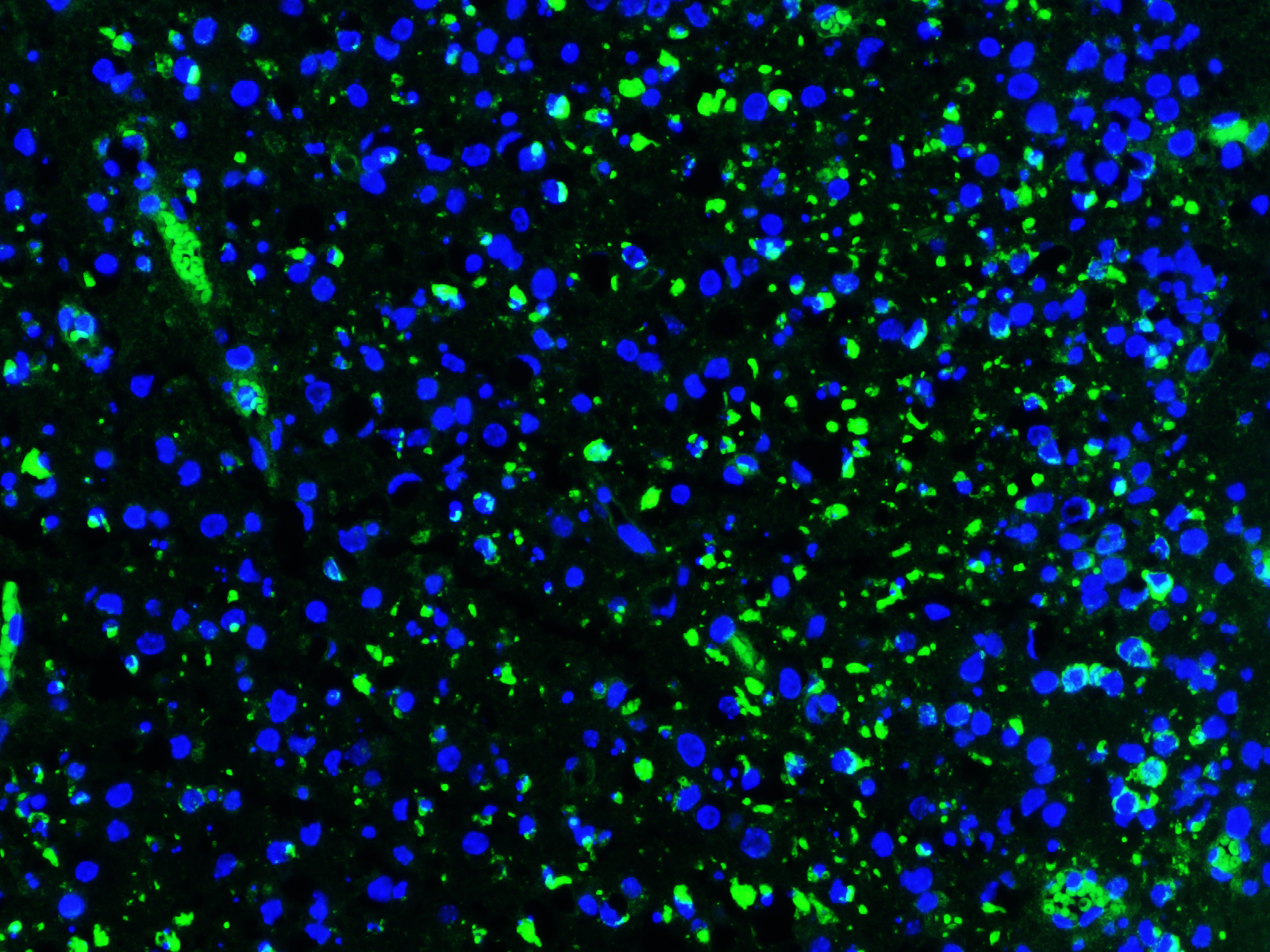

Supplement: Supplementary file 4 — Source data Fig. 2 [file 44321_2025_304_MOESM4_ESM.zip › Fig 2/Fig 2D/WT d9.tif]

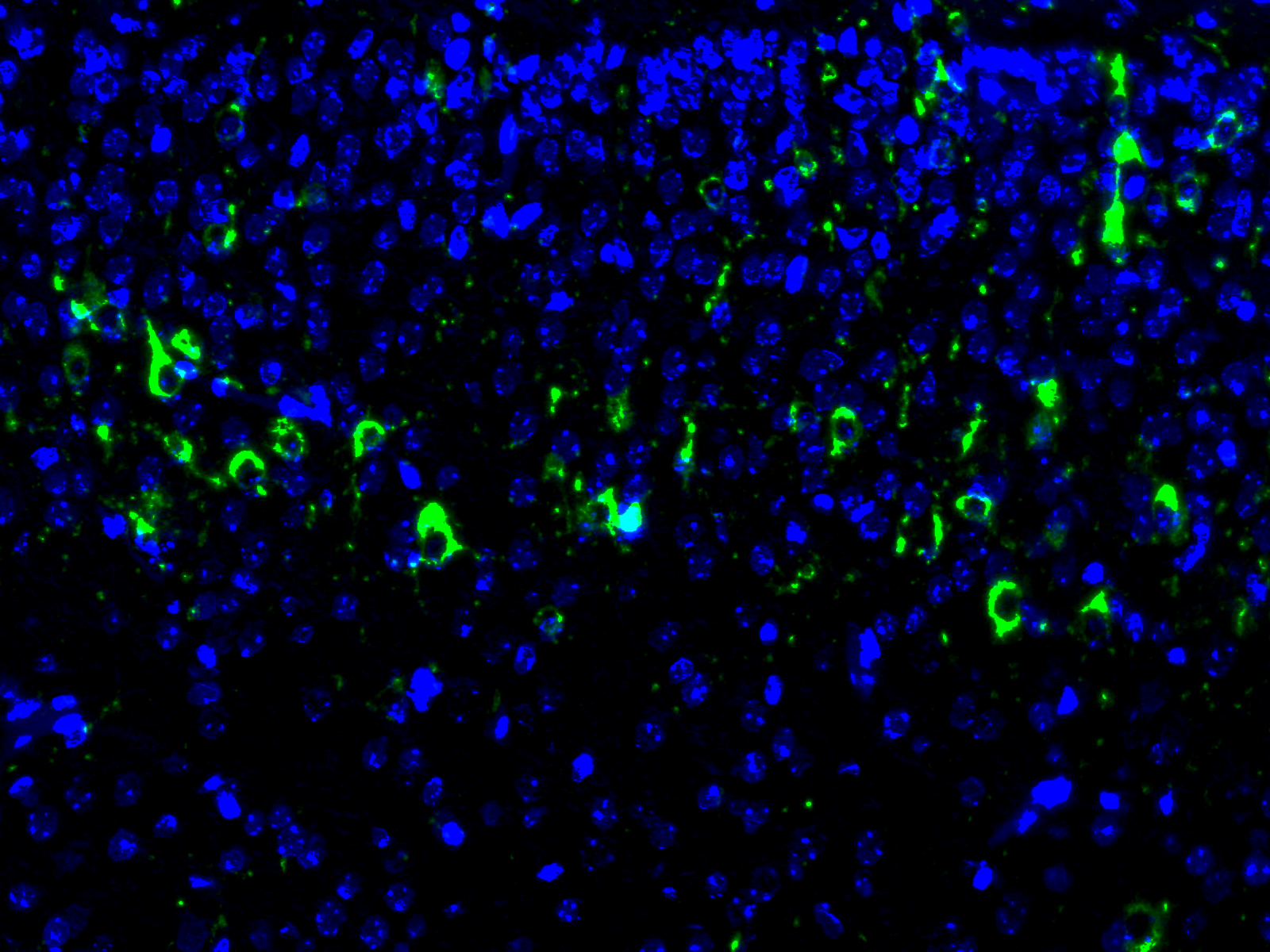

Supplement: Supplementary file 4 — Source data Fig. 2 [file 44321_2025_304_MOESM4_ESM.zip › Fig 2/Fig 2D/MBD1 d6.tif]

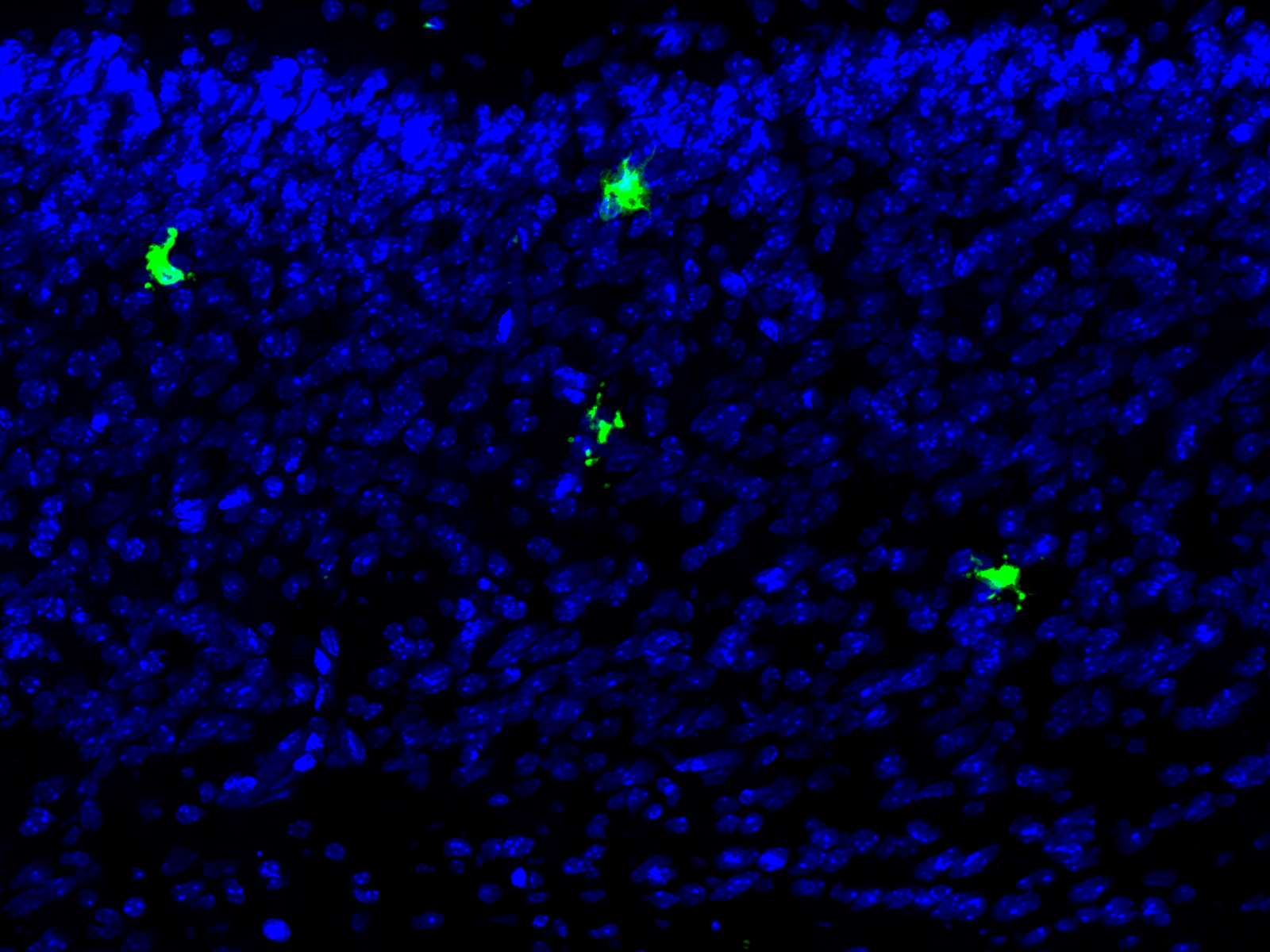

Supplement: Supplementary file 4 — Source data Fig. 2 [file 44321_2025_304_MOESM4_ESM.zip › Fig 2/Fig 2D/MBD1 d3.tif]

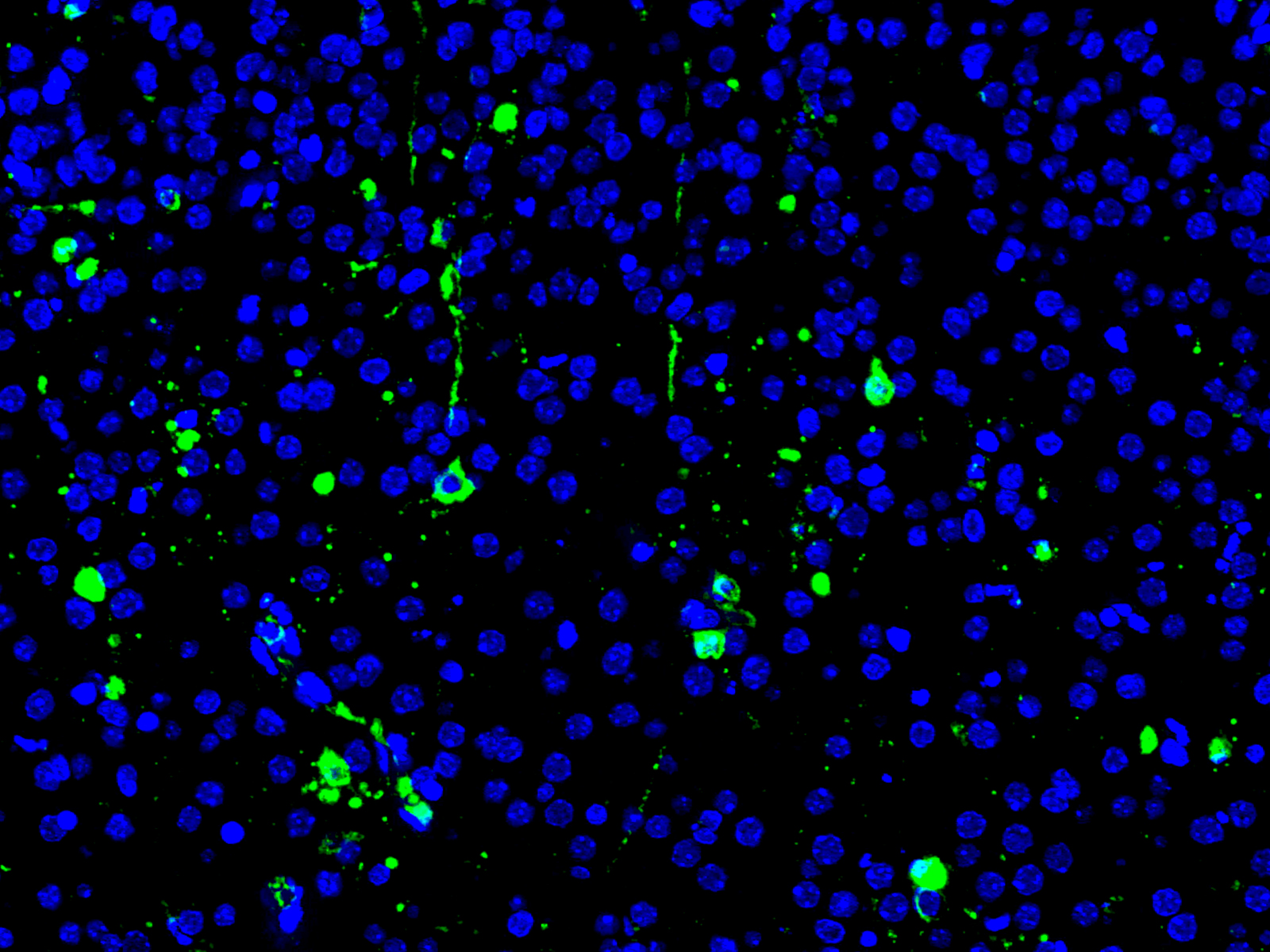

Supplement: Supplementary file 4 — Source data Fig. 2 [file 44321_2025_304_MOESM4_ESM.zip › Fig 2/Fig 2D/MBD1 d9.tif]

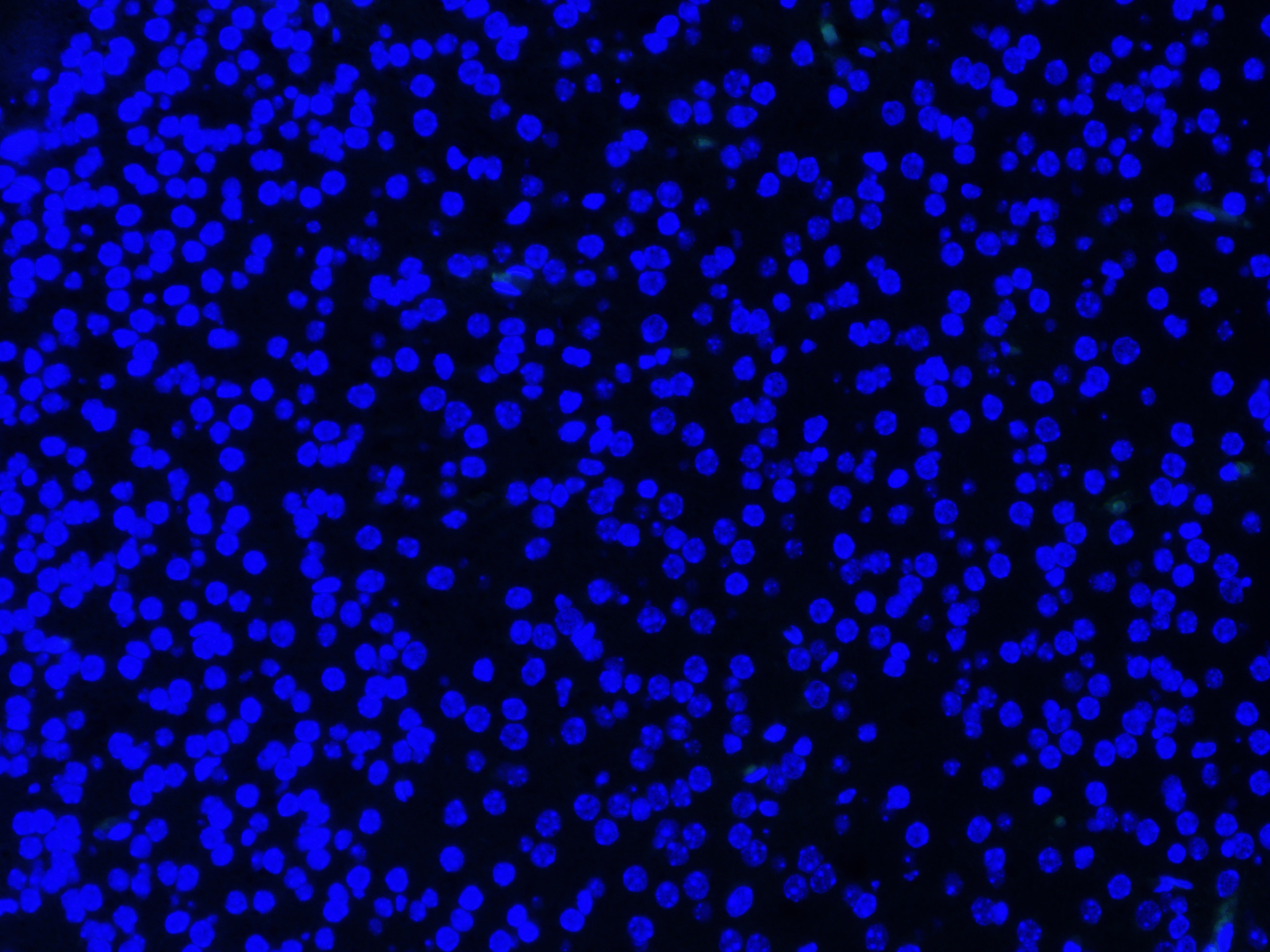

Supplement: Supplementary file 4 — Source data Fig. 2 [file 44321_2025_304_MOESM4_ESM.zip › Fig 2/Fig 2D/Mock d6.tif]

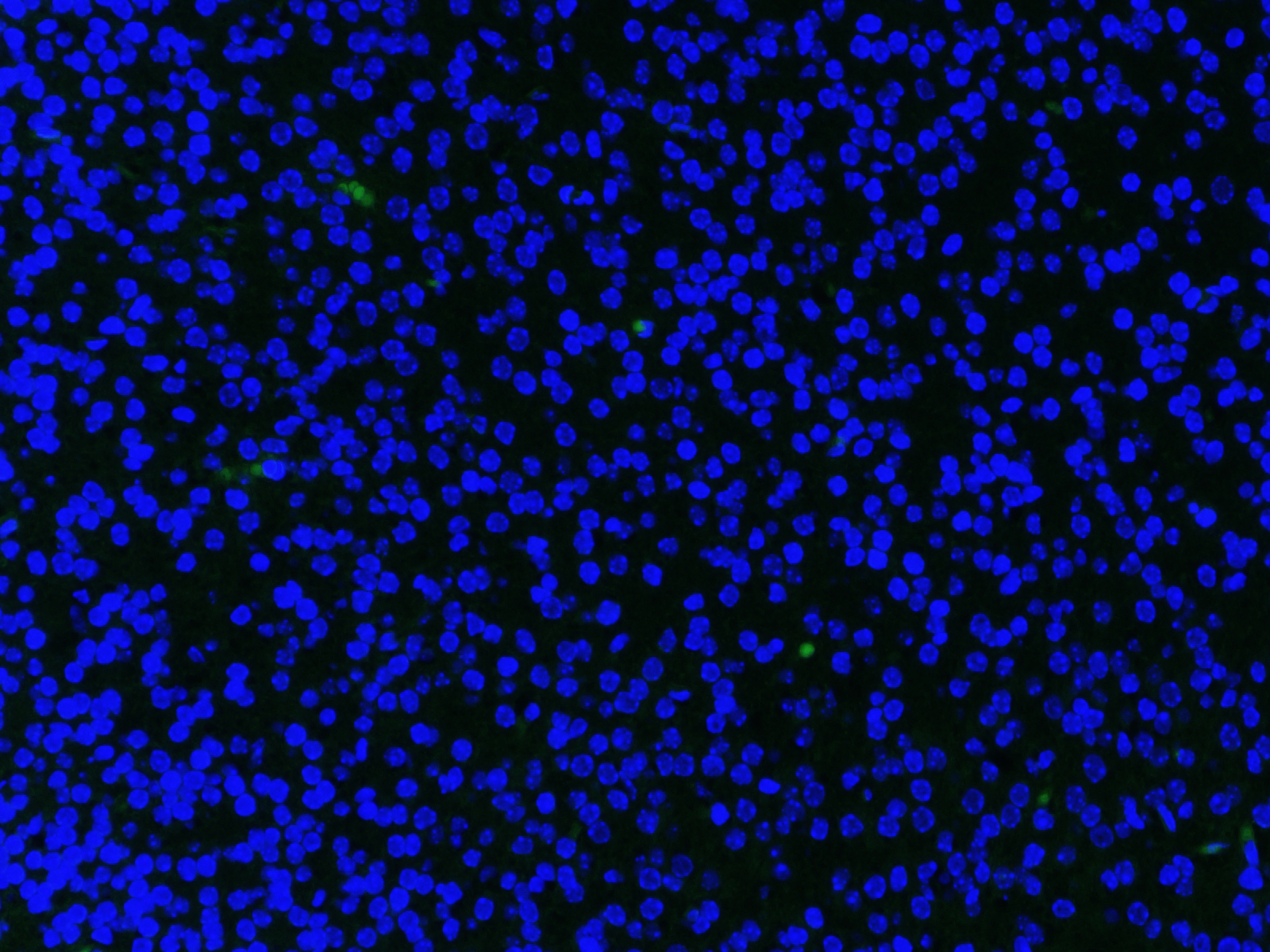

Supplement: Supplementary file 4 — Source data Fig. 2 [file 44321_2025_304_MOESM4_ESM.zip › Fig 2/Fig 2D/MBD2 d3.tif]

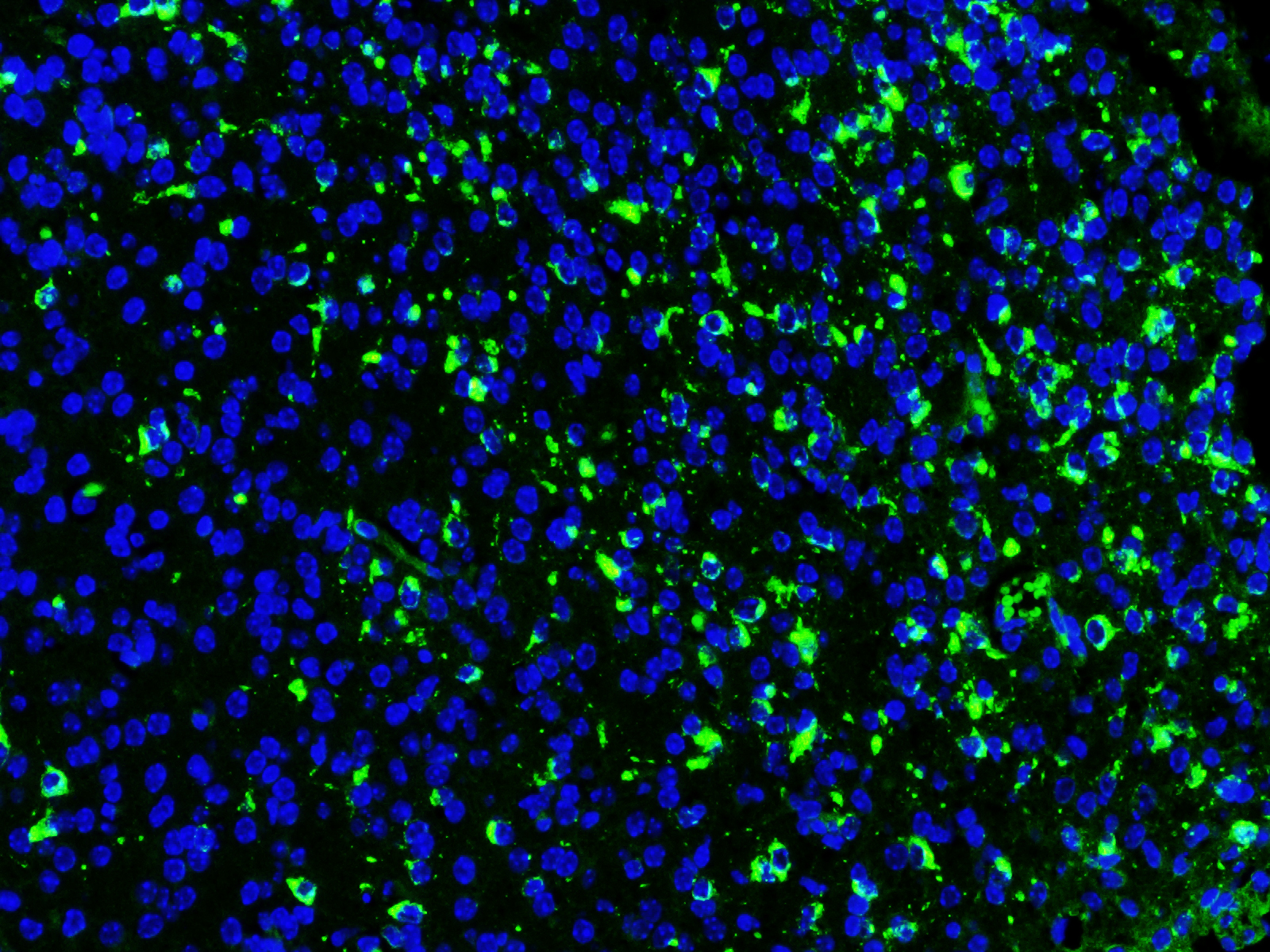

Supplement: Supplementary file 4 — Source data Fig. 2 [file 44321_2025_304_MOESM4_ESM.zip › Fig 2/Fig 2D/WT d6.tif]

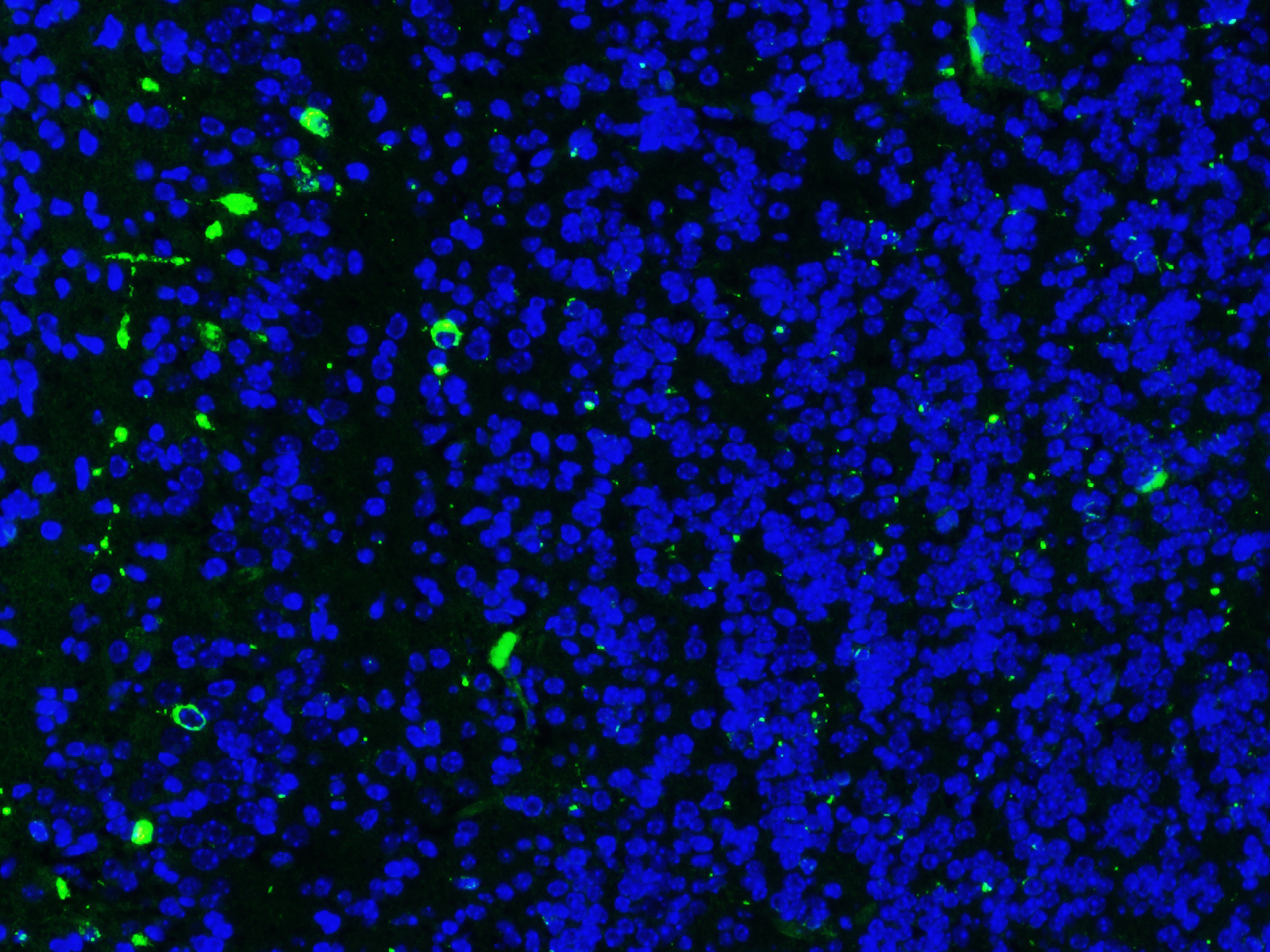

Supplement: Supplementary file 4 — Source data Fig. 2 [file 44321_2025_304_MOESM4_ESM.zip › Fig 2/Fig 2D/WT d3.tif]

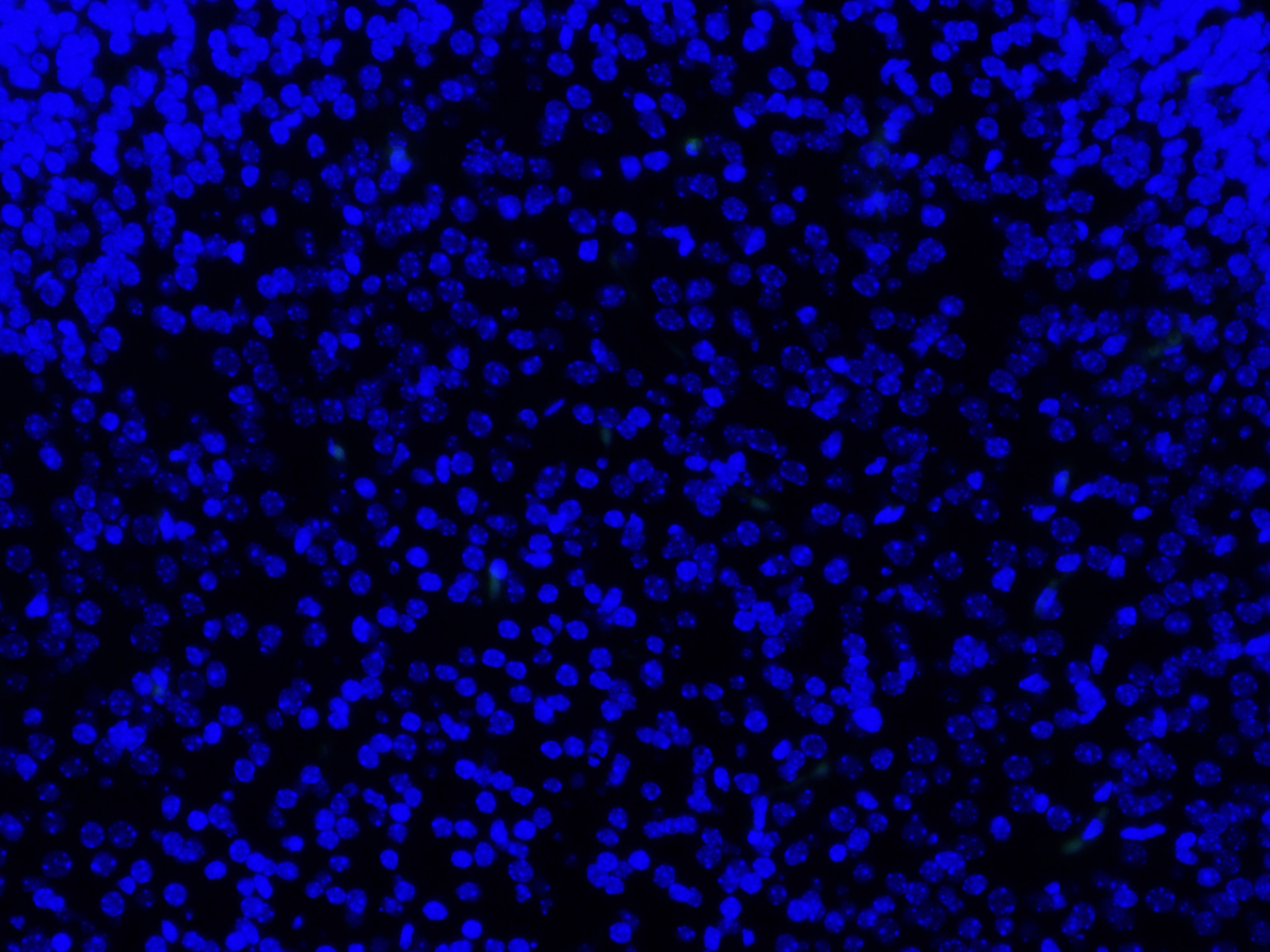

Supplement: Supplementary file 4 — Source data Fig. 2 [file 44321_2025_304_MOESM4_ESM.zip › Fig 2/Fig 2D/Mock d3.tif]

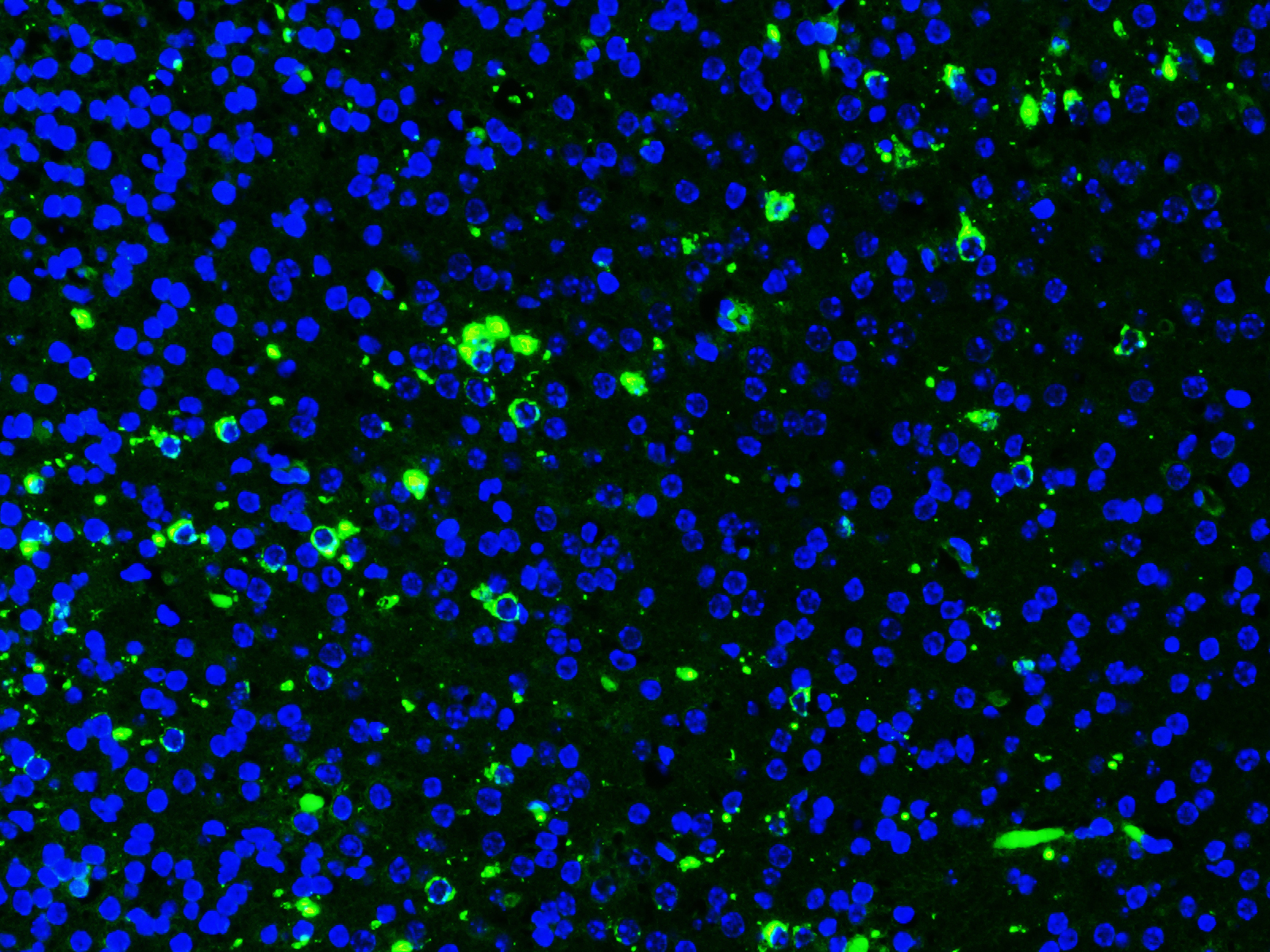

Supplement: Supplementary file 4 — Source data Fig. 2 [file 44321_2025_304_MOESM4_ESM.zip › Fig 2/Fig 2D/MBD2 d6.tif]

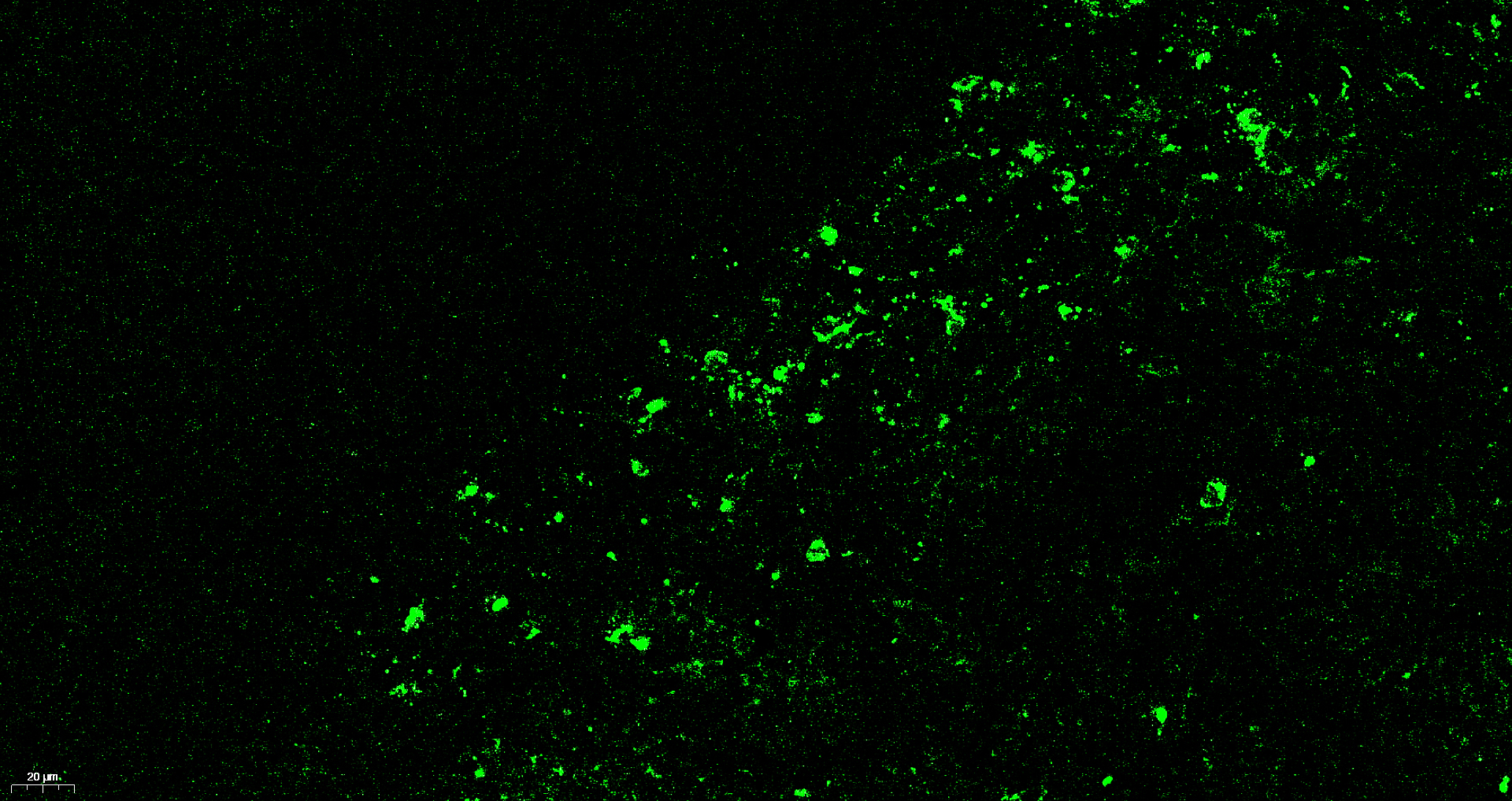

Supplement: Supplementary file 5 — Source data Fig. 3 [file 44321_2025_304_MOESM5_ESM.zip › Fig 3/Fig 3D/WT ZIKVE.tif]

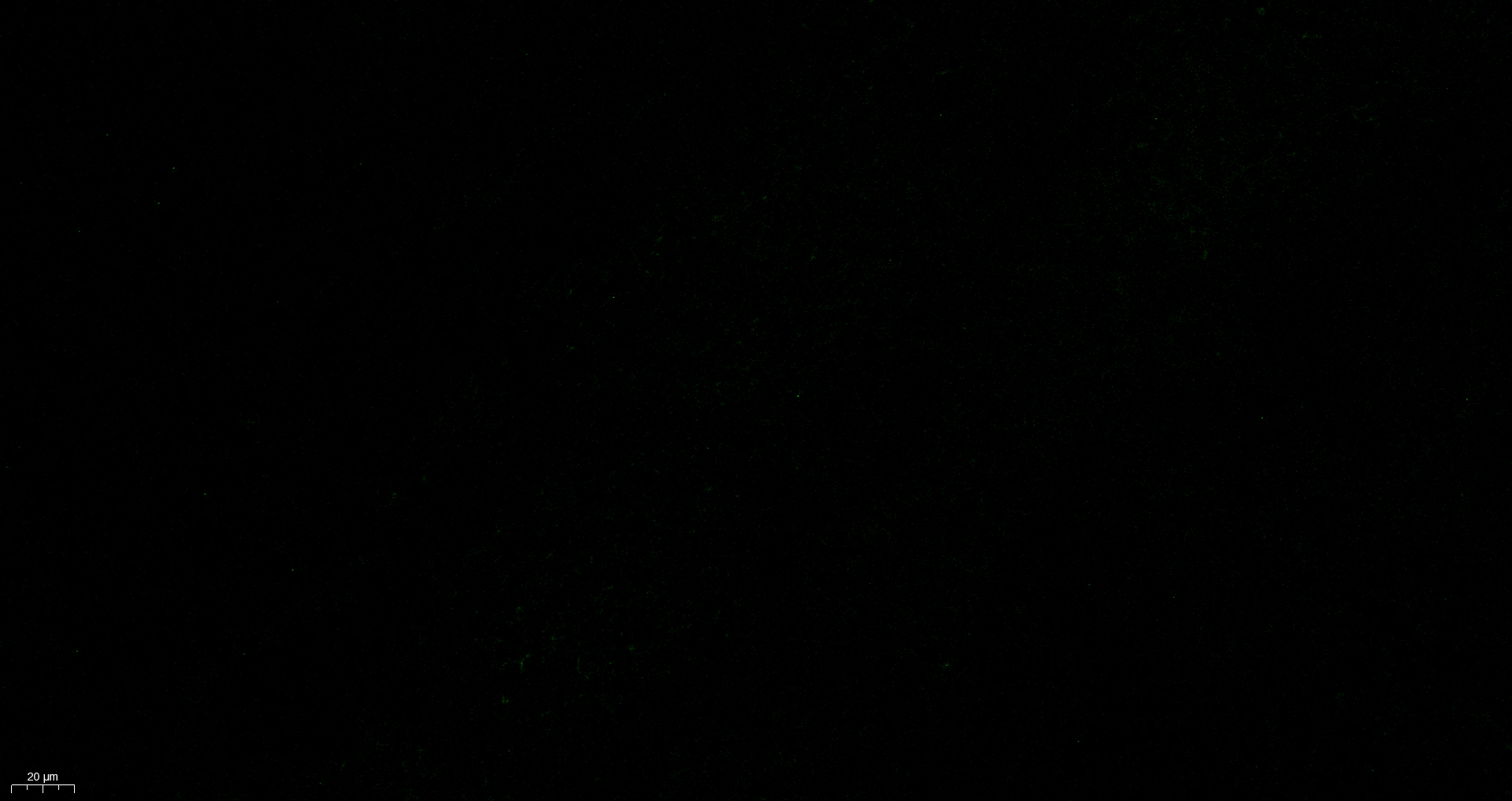

Supplement: Supplementary file 5 — Source data Fig. 3 [file 44321_2025_304_MOESM5_ESM.zip › Fig 3/Fig 3D/Mock ZIKVE.tif]

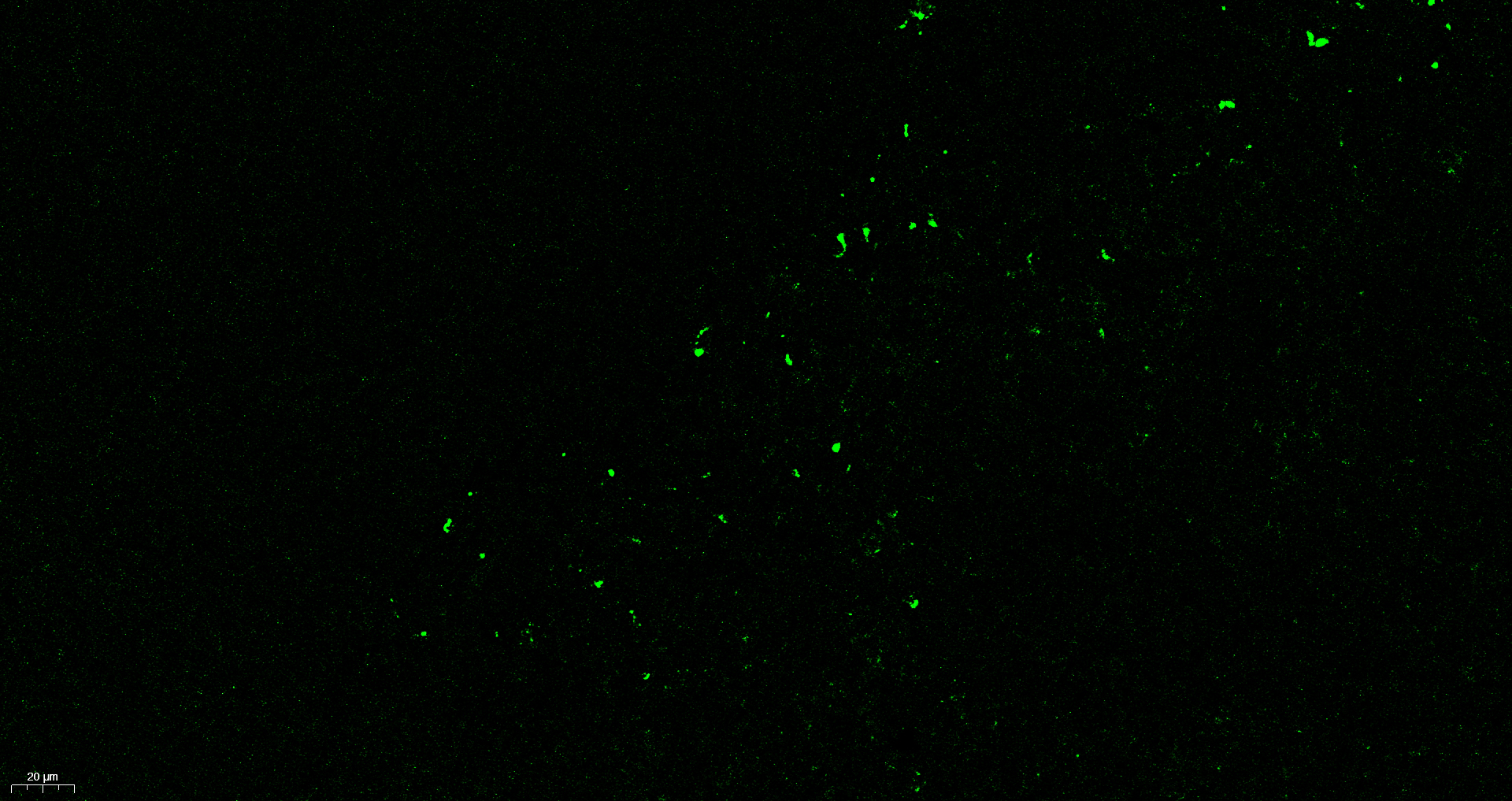

Supplement: Supplementary file 5 — Source data Fig. 3 [file 44321_2025_304_MOESM5_ESM.zip › Fig 3/Fig 3D/MBD2 ZIKVE.tif]

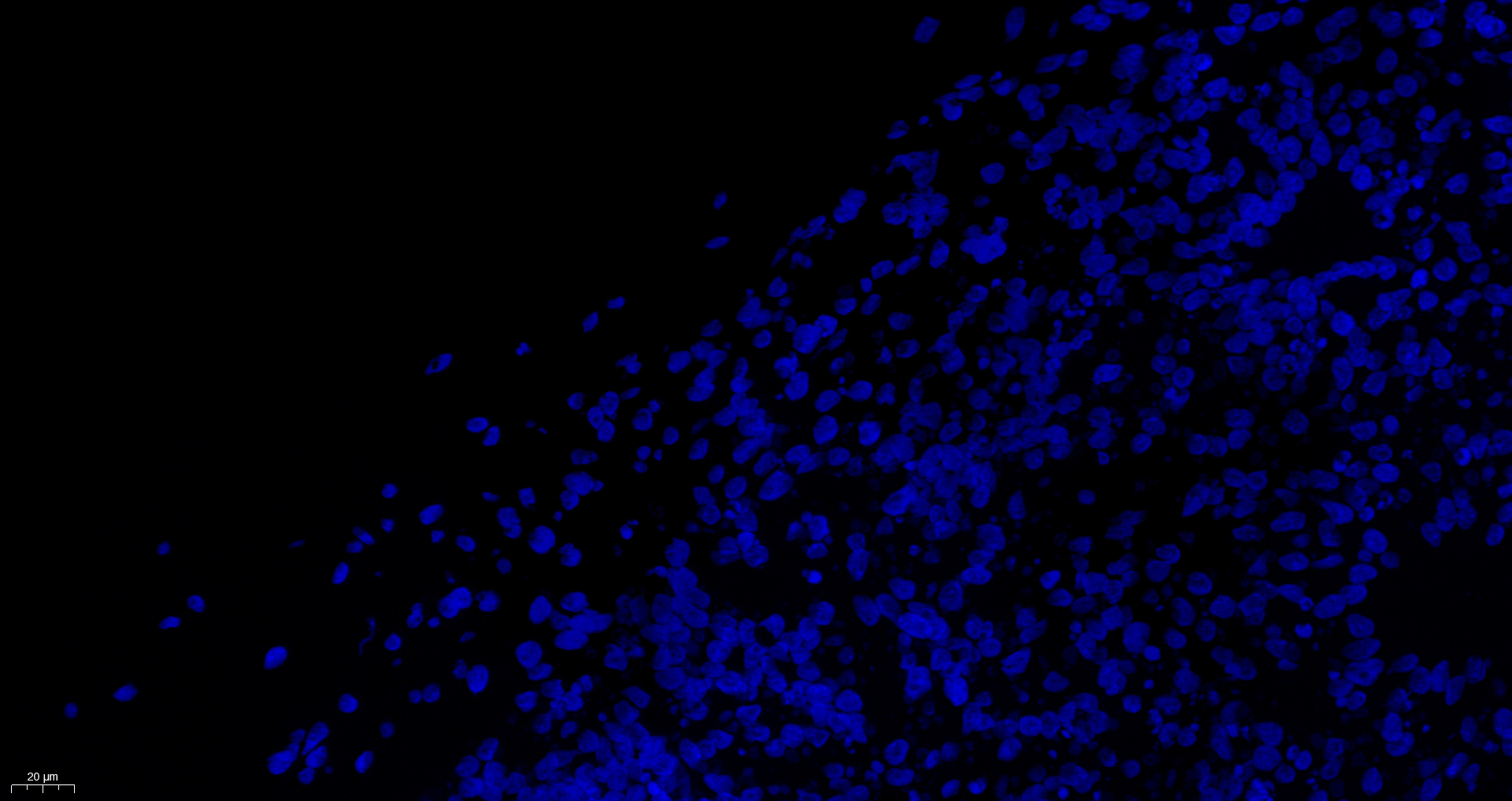

Supplement: Supplementary file 5 — Source data Fig. 3 [file 44321_2025_304_MOESM5_ESM.zip › Fig 3/Fig 3D/WT DAPI.tif]

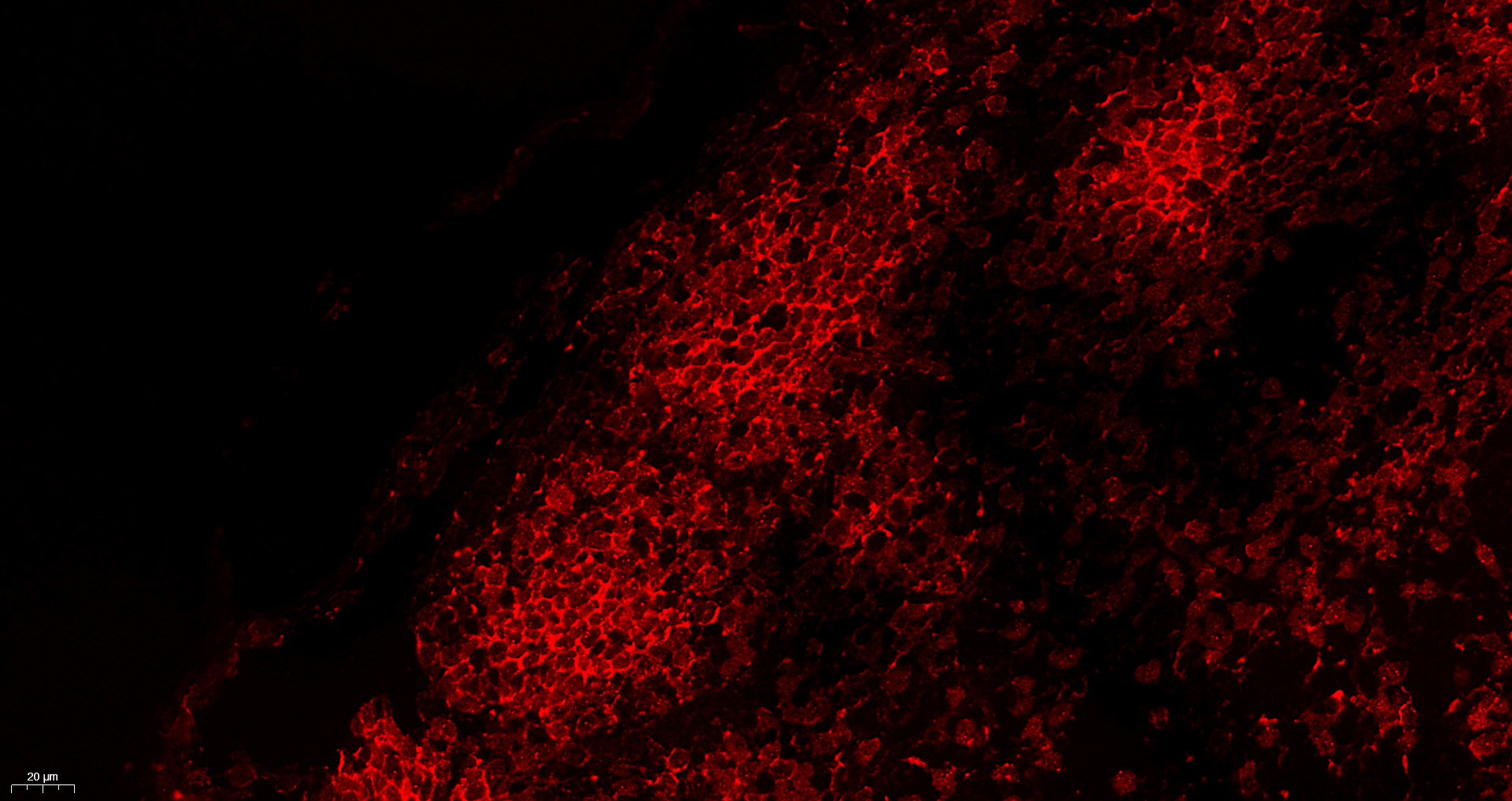

Supplement: Supplementary file 5 — Source data Fig. 3 [file 44321_2025_304_MOESM5_ESM.zip › Fig 3/Fig 3D/Mock MSI1.tif]

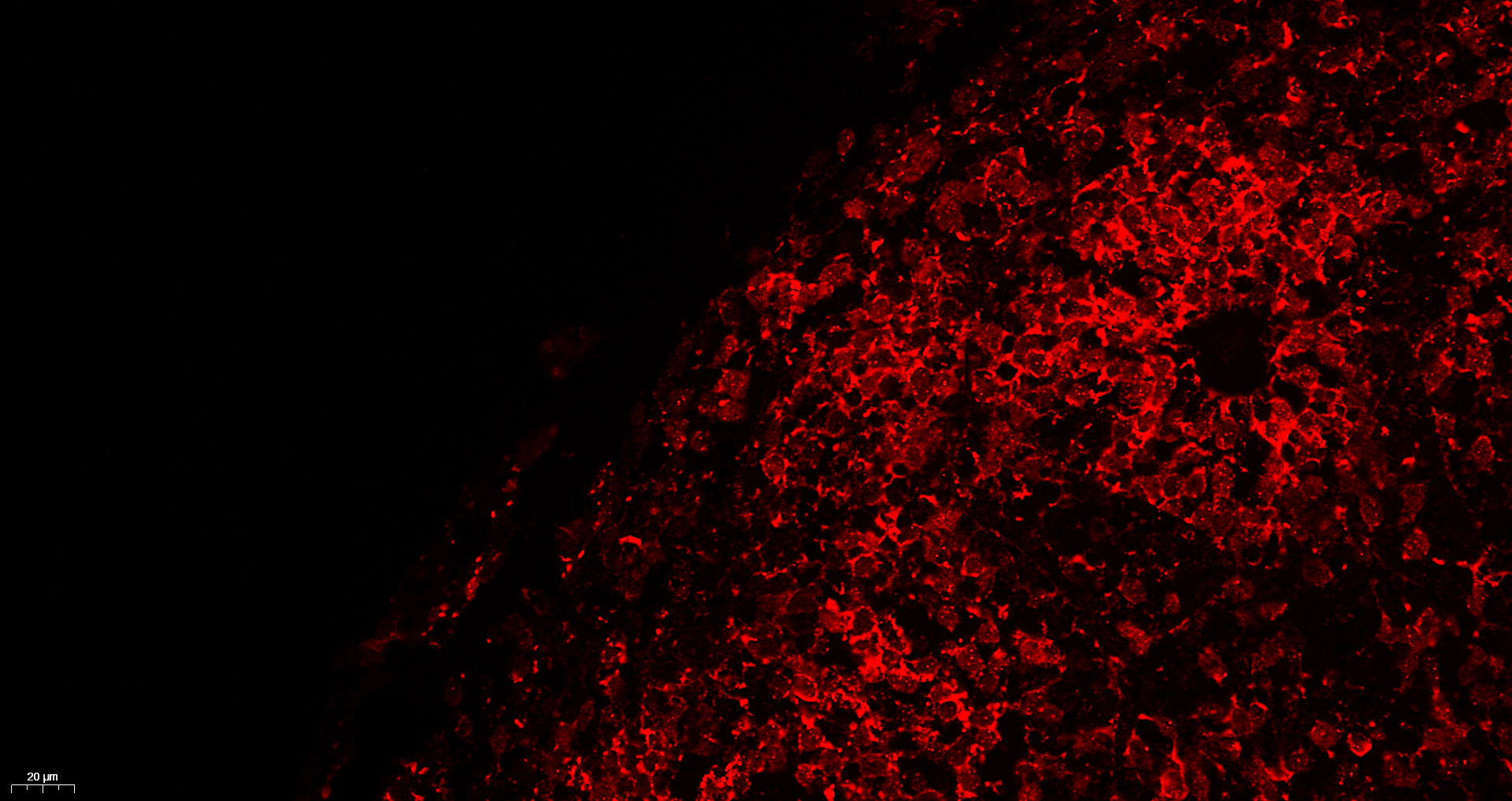

Supplement: Supplementary file 5 — Source data Fig. 3 [file 44321_2025_304_MOESM5_ESM.zip › Fig 3/Fig 3D/MBD2 MSI1.tif]

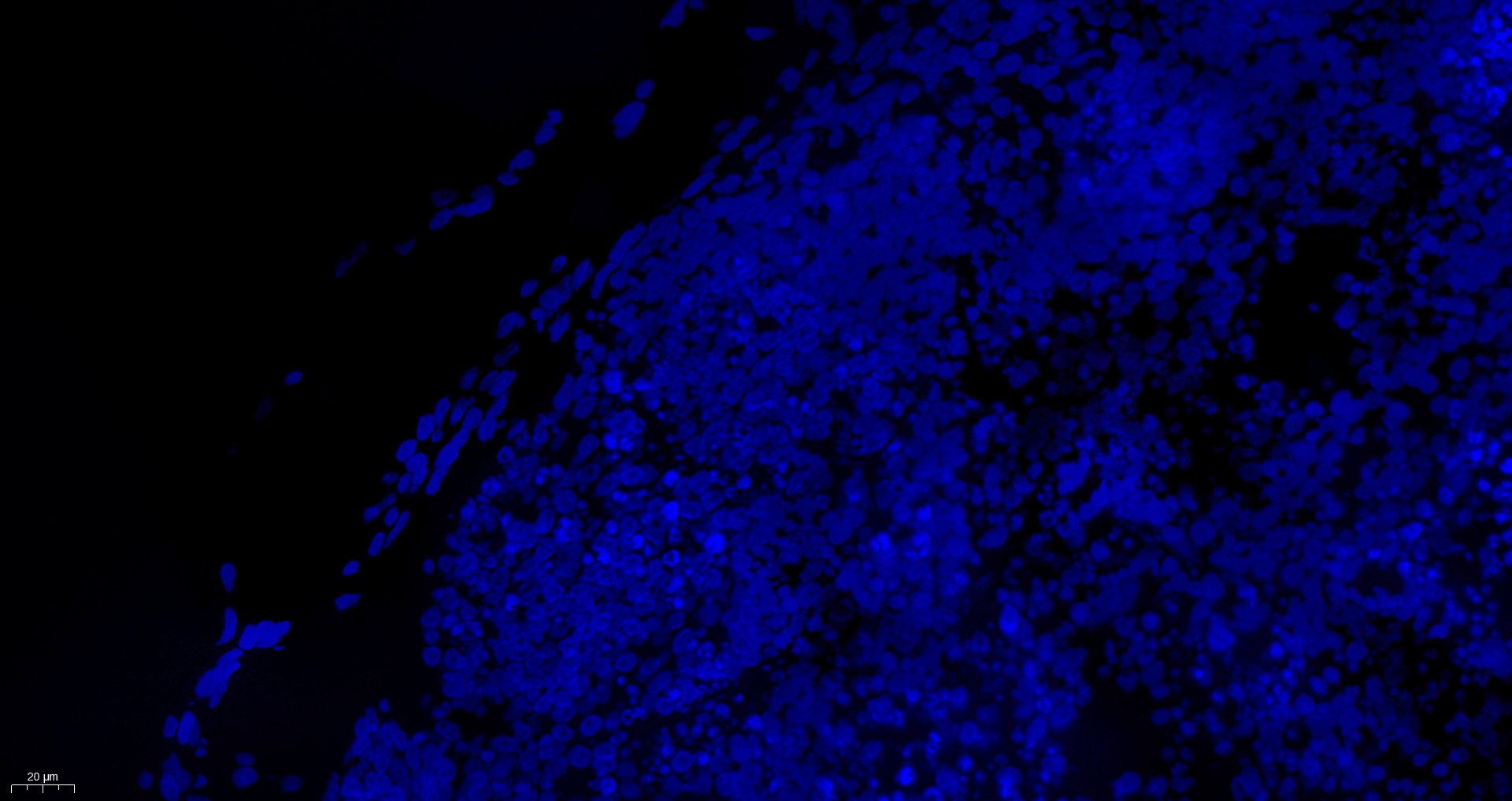

Supplement: Supplementary file 5 — Source data Fig. 3 [file 44321_2025_304_MOESM5_ESM.zip › Fig 3/Fig 3D/Mock DAPI.tif]

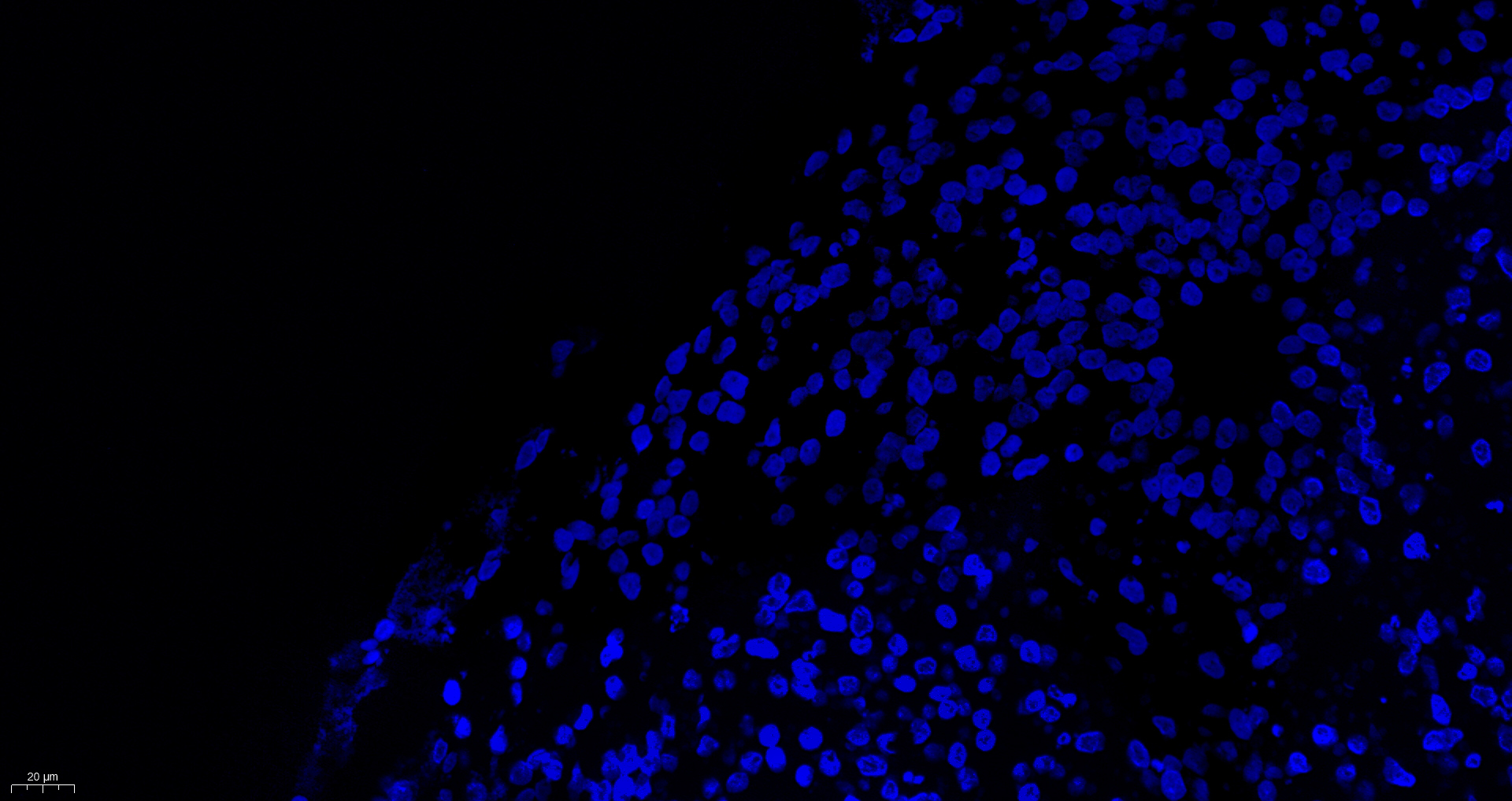

Supplement: Supplementary file 5 — Source data Fig. 3 [file 44321_2025_304_MOESM5_ESM.zip › Fig 3/Fig 3D/MBD2 DAPI.tif]

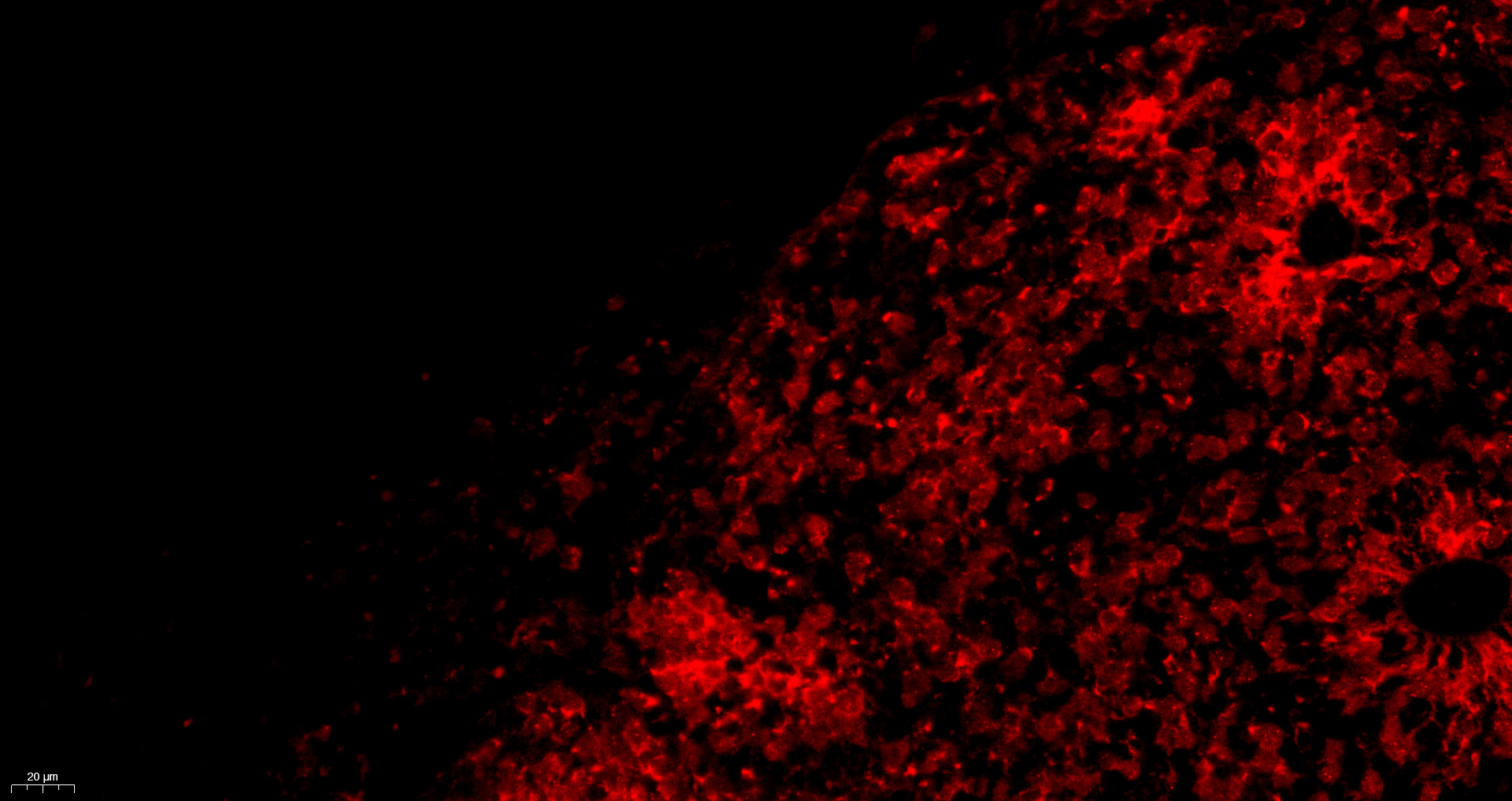

Supplement: Supplementary file 5 — Source data Fig. 3 [file 44321_2025_304_MOESM5_ESM.zip › Fig 3/Fig 3D/WT MSI1.tif]

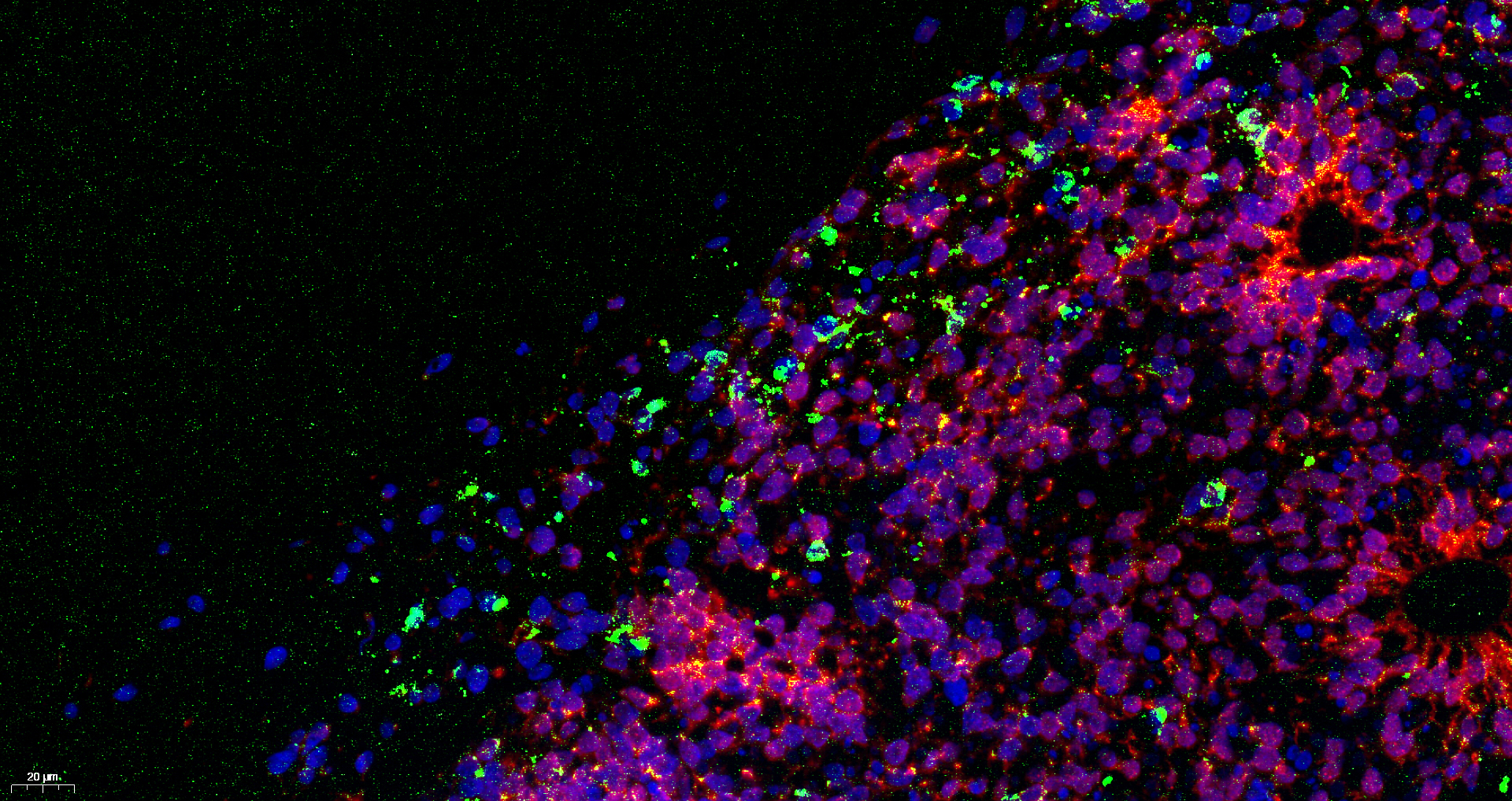

Supplement: Supplementary file 5 — Source data Fig. 3 [file 44321_2025_304_MOESM5_ESM.zip › Fig 3/Fig 3D/WT Merge.tif]

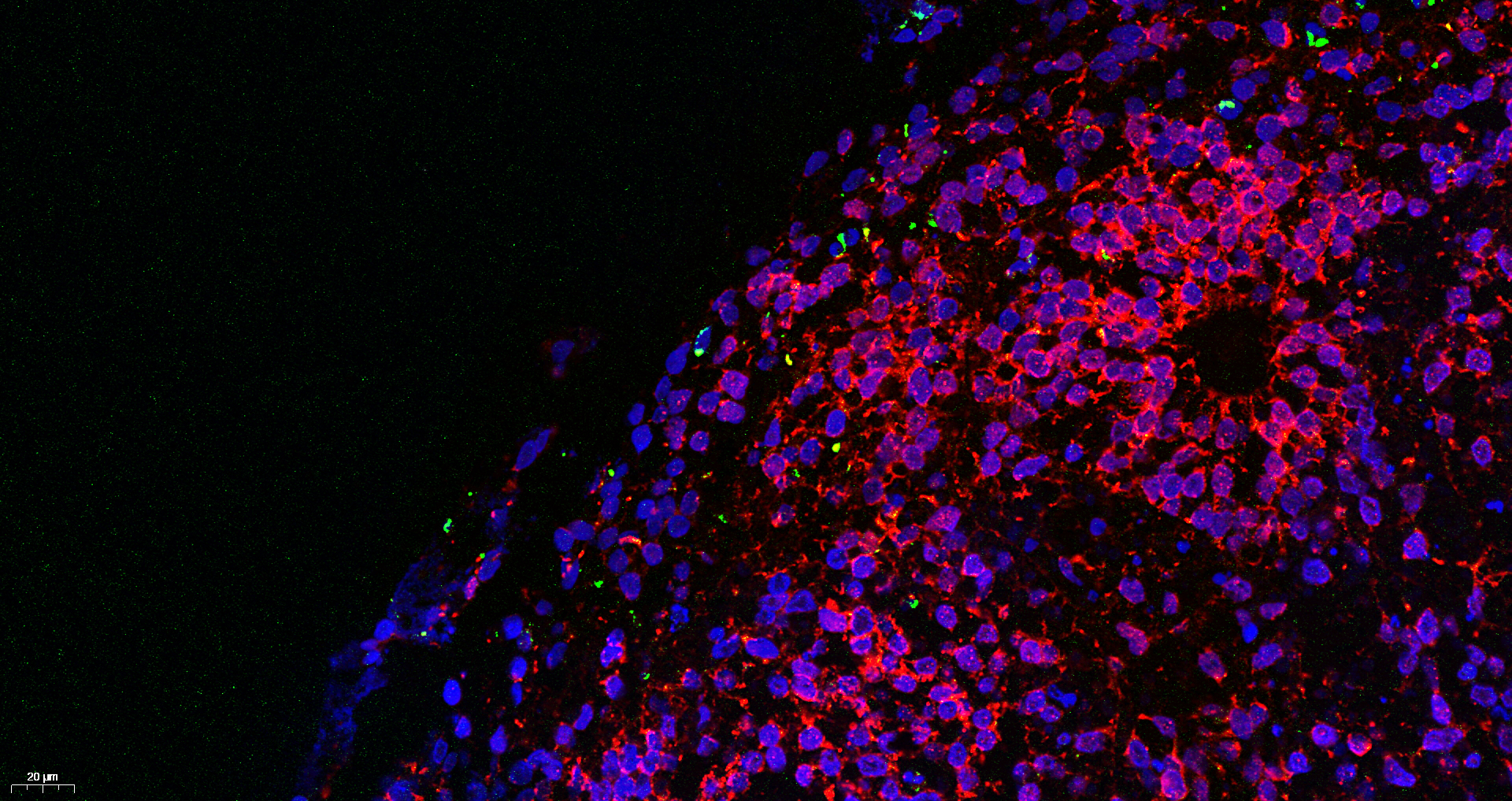

Supplement: Supplementary file 5 — Source data Fig. 3 [file 44321_2025_304_MOESM5_ESM.zip › Fig 3/Fig 3D/MBD2 Merge.tif]

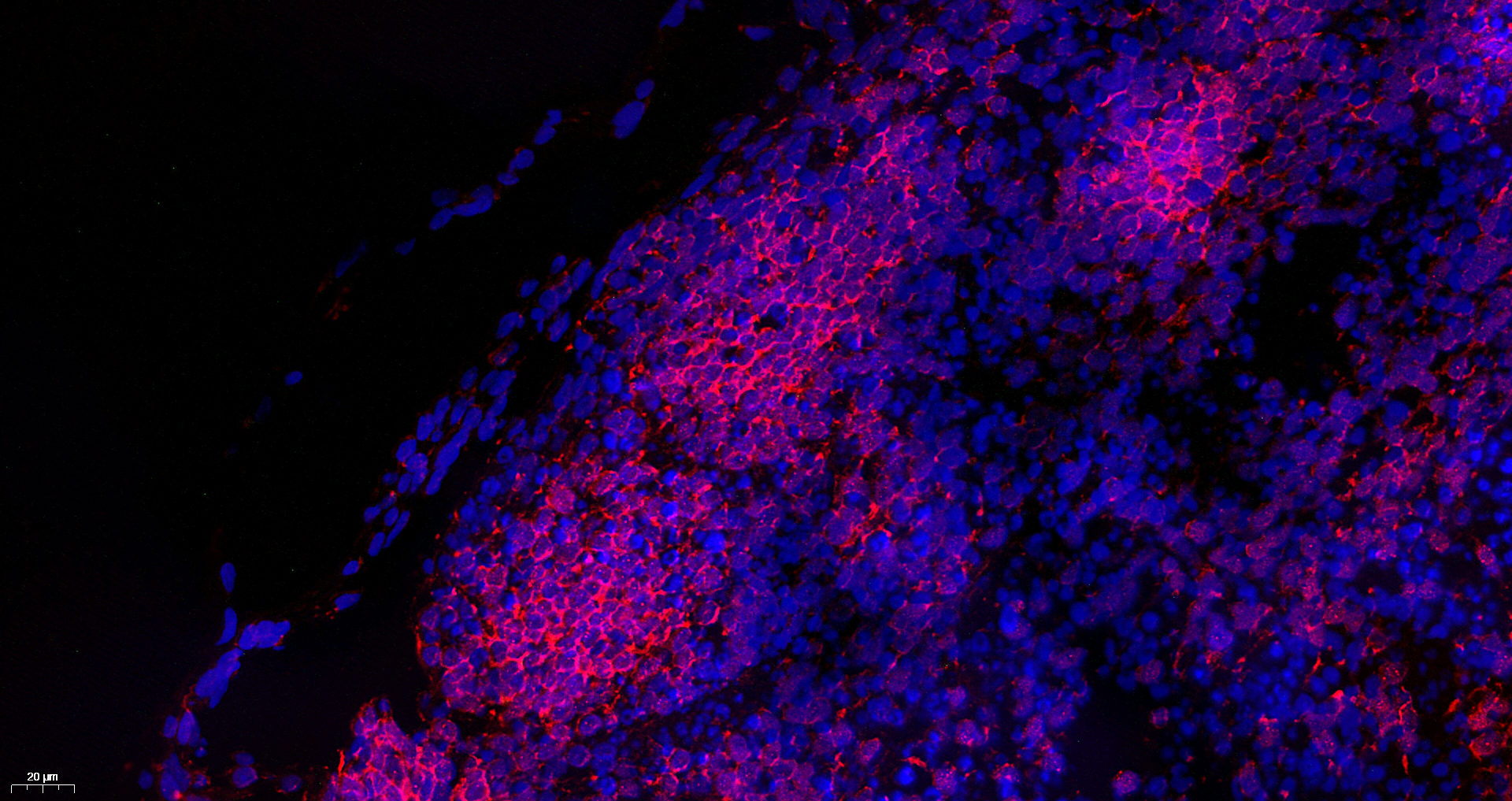

Supplement: Supplementary file 5 — Source data Fig. 3 [file 44321_2025_304_MOESM5_ESM.zip › Fig 3/Fig 3D/Mock Merge.tif]

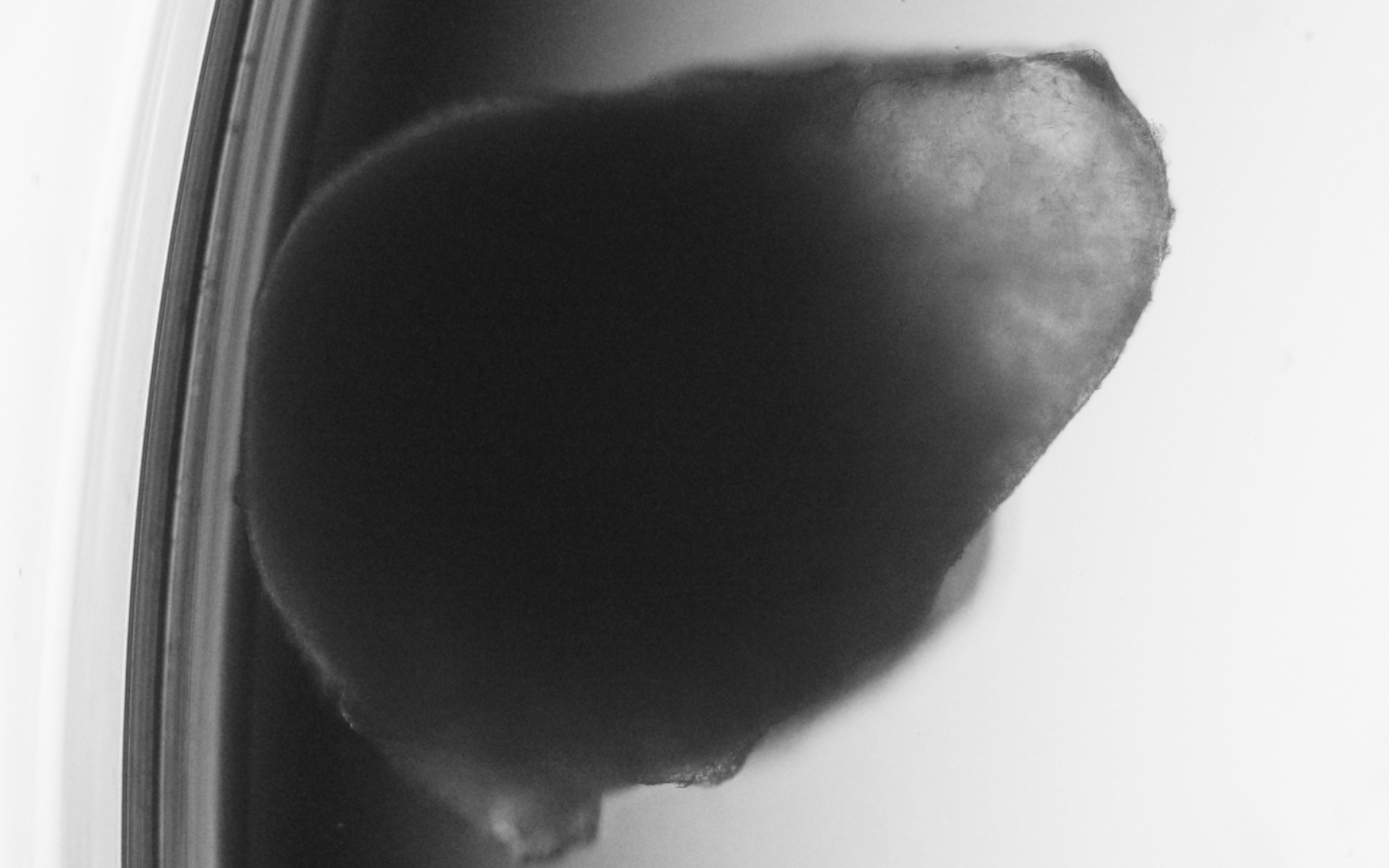

Supplement: Supplementary file 5 — Source data Fig. 3 [file 44321_2025_304_MOESM5_ESM.zip › Fig 3/Fig 3A/MBD2 d18.tif]

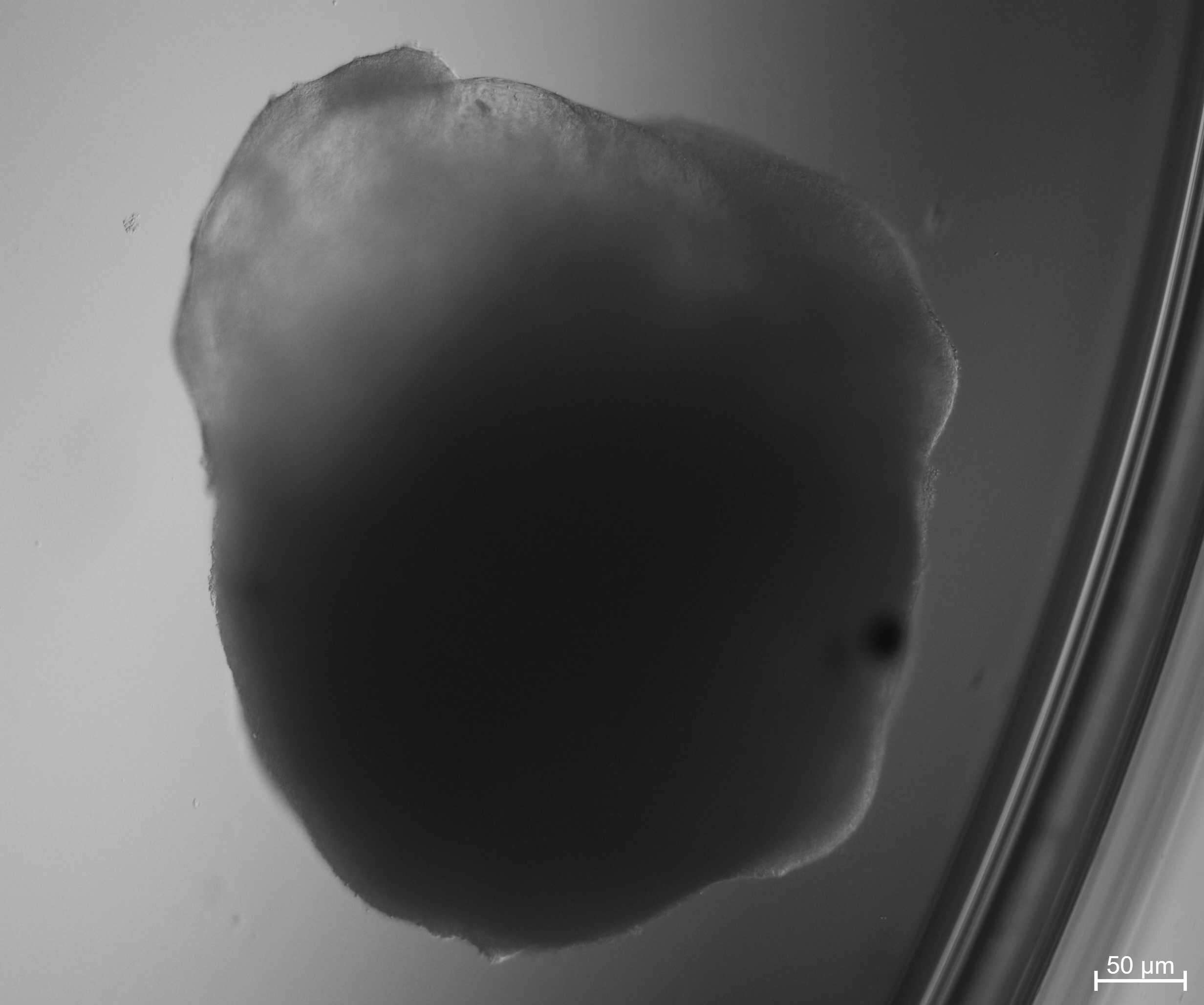

Supplement: Supplementary file 5 — Source data Fig. 3 [file 44321_2025_304_MOESM5_ESM.zip › Fig 3/Fig 3A/MBD2 d30.tif]

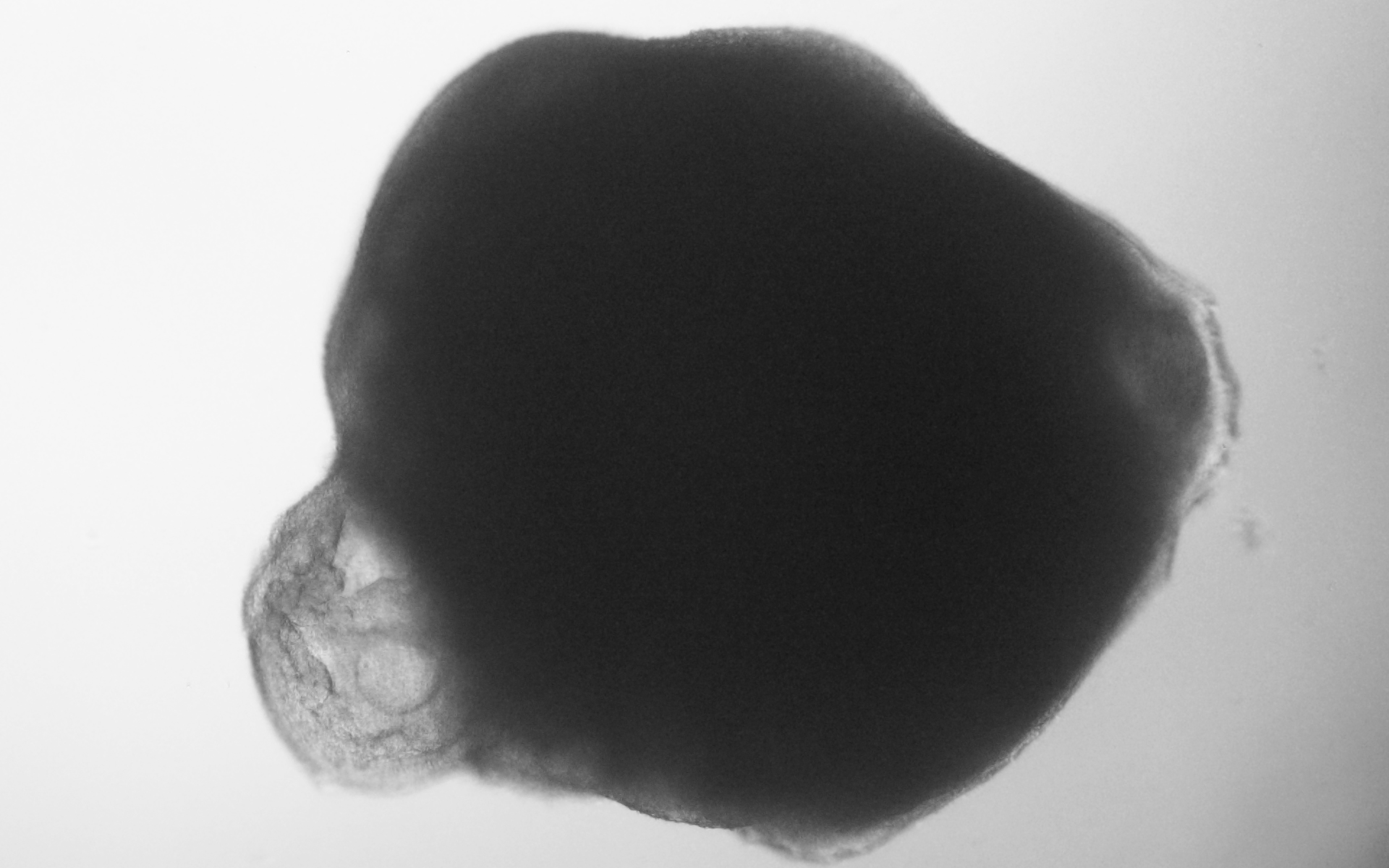

Supplement: Supplementary file 5 — Source data Fig. 3 [file 44321_2025_304_MOESM5_ESM.zip › Fig 3/Fig 3A/Mock d18.tif]

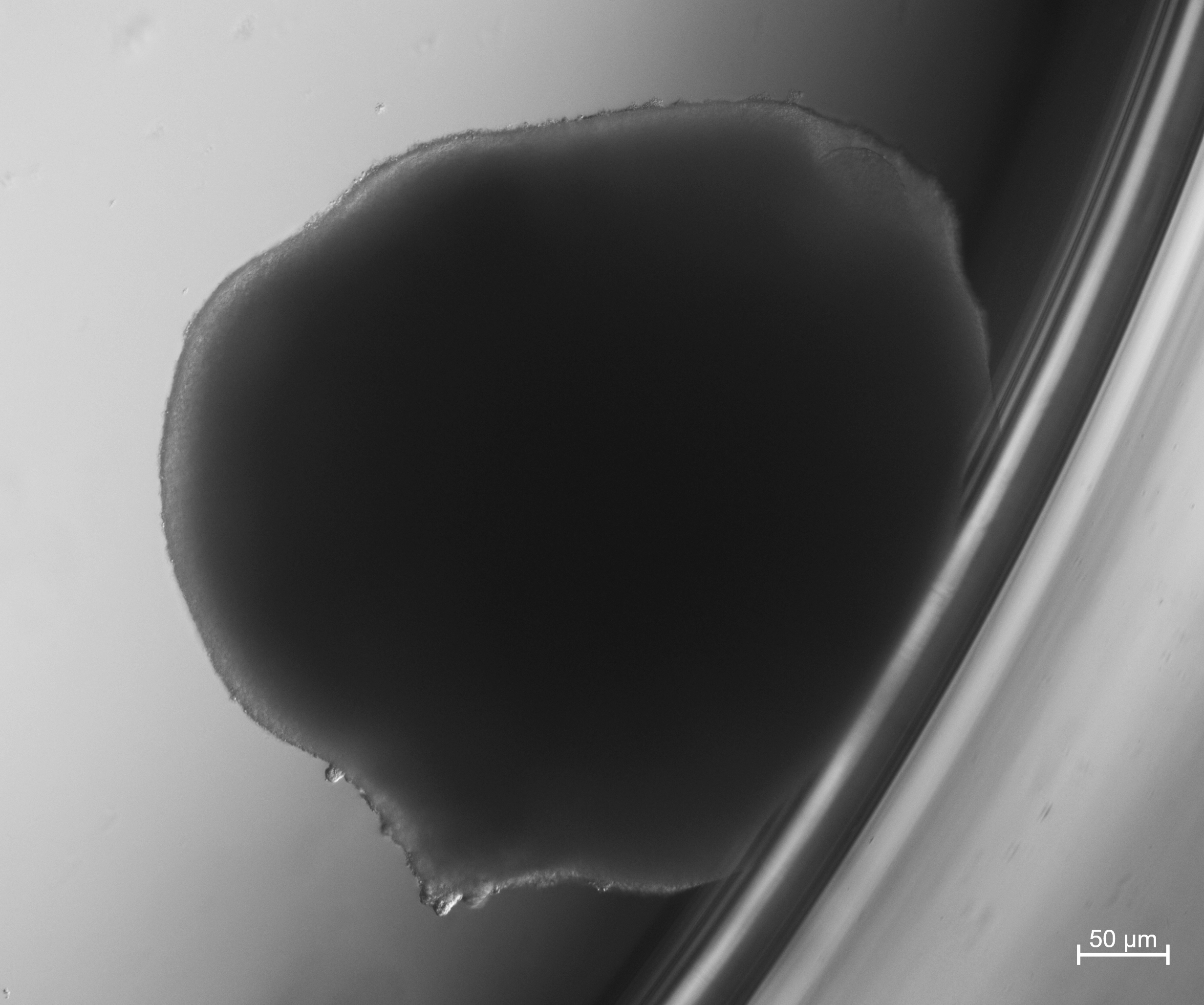

Supplement: Supplementary file 5 — Source data Fig. 3 [file 44321_2025_304_MOESM5_ESM.zip › Fig 3/Fig 3A/Mock d30.tif]

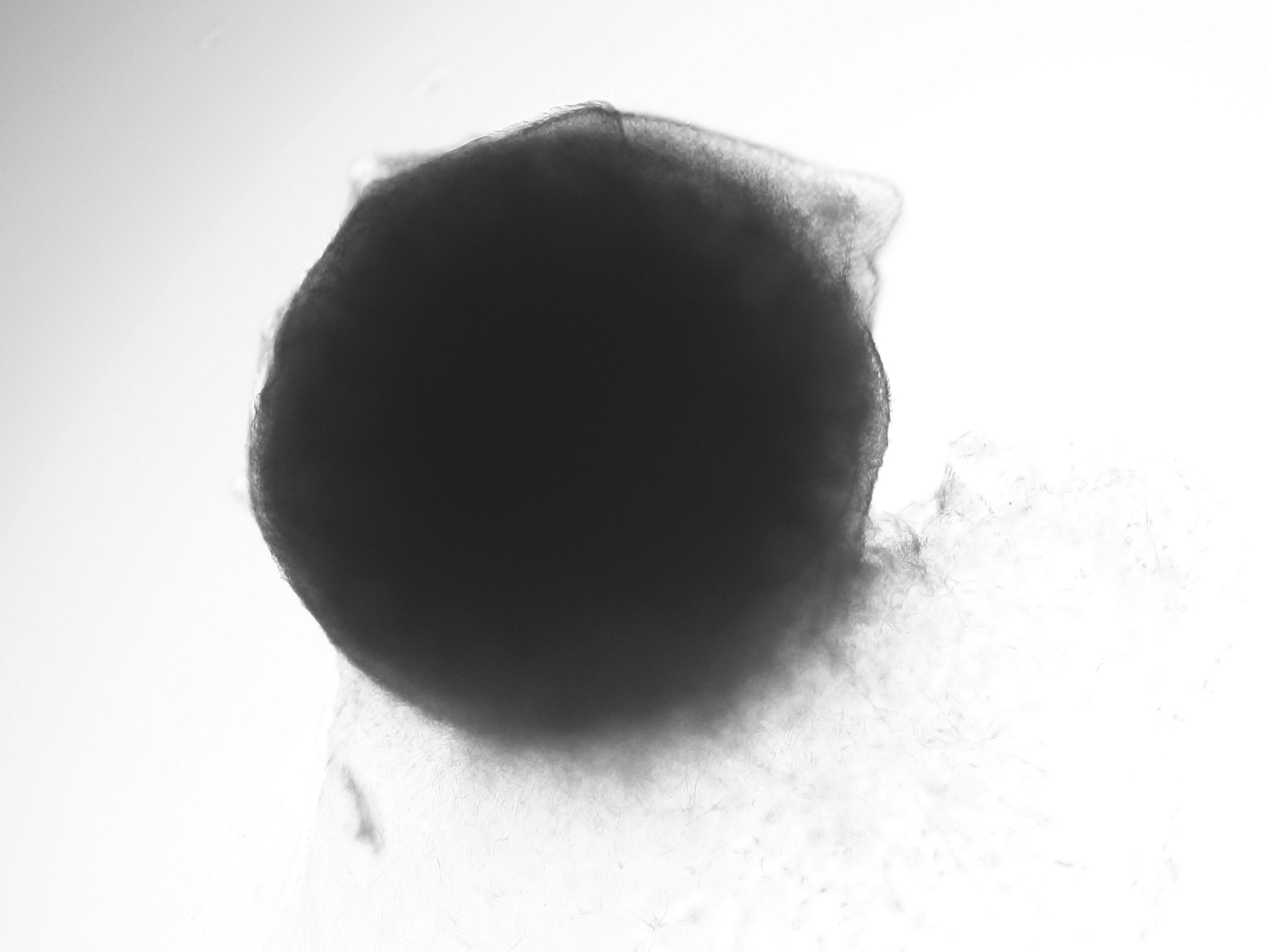

Supplement: Supplementary file 5 — Source data Fig. 3 [file 44321_2025_304_MOESM5_ESM.zip › Fig 3/Fig 3A/WT d5.tif]

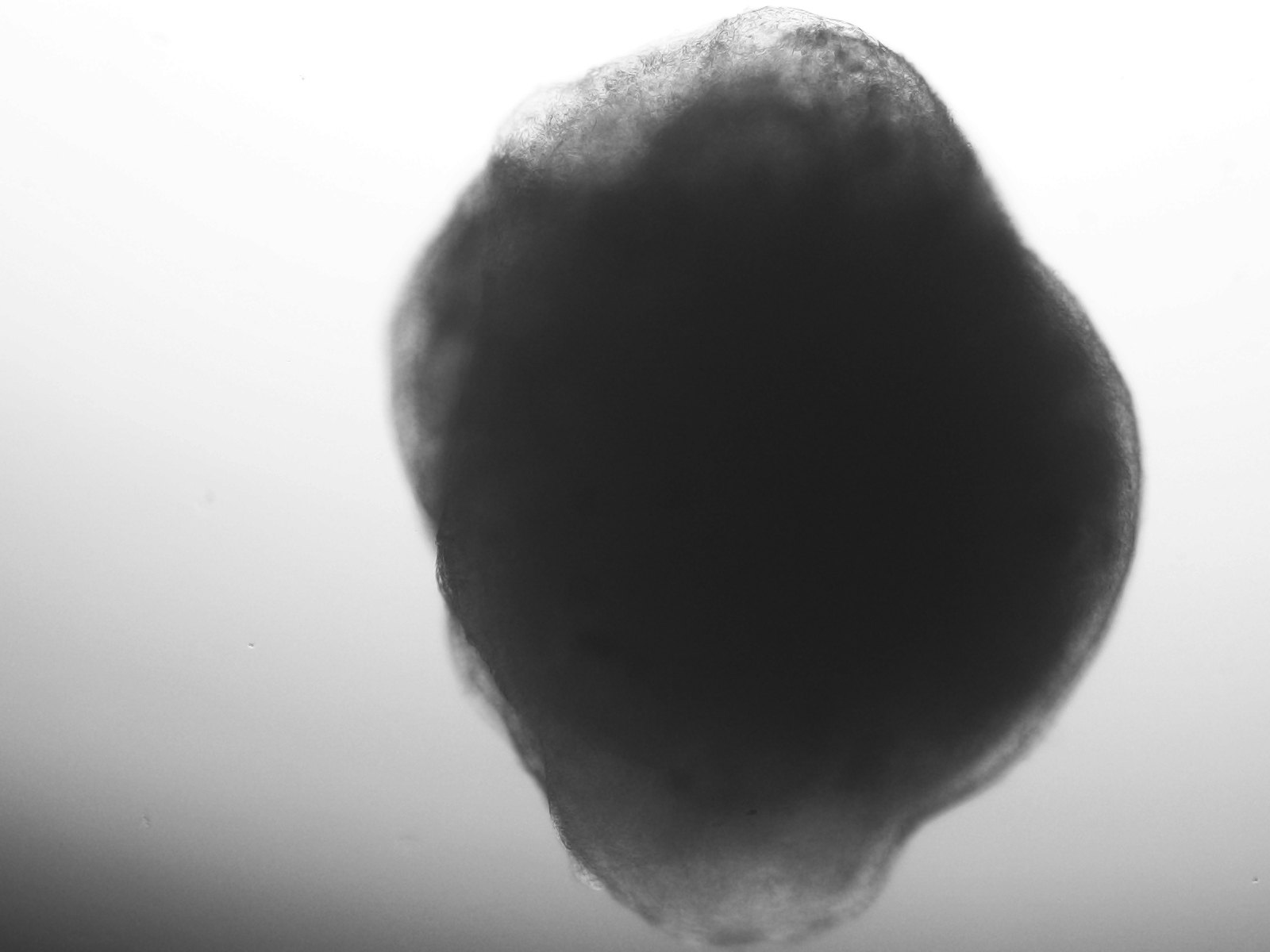

Supplement: Supplementary file 5 — Source data Fig. 3 [file 44321_2025_304_MOESM5_ESM.zip › Fig 3/Fig 3A/Mock d5.tif]

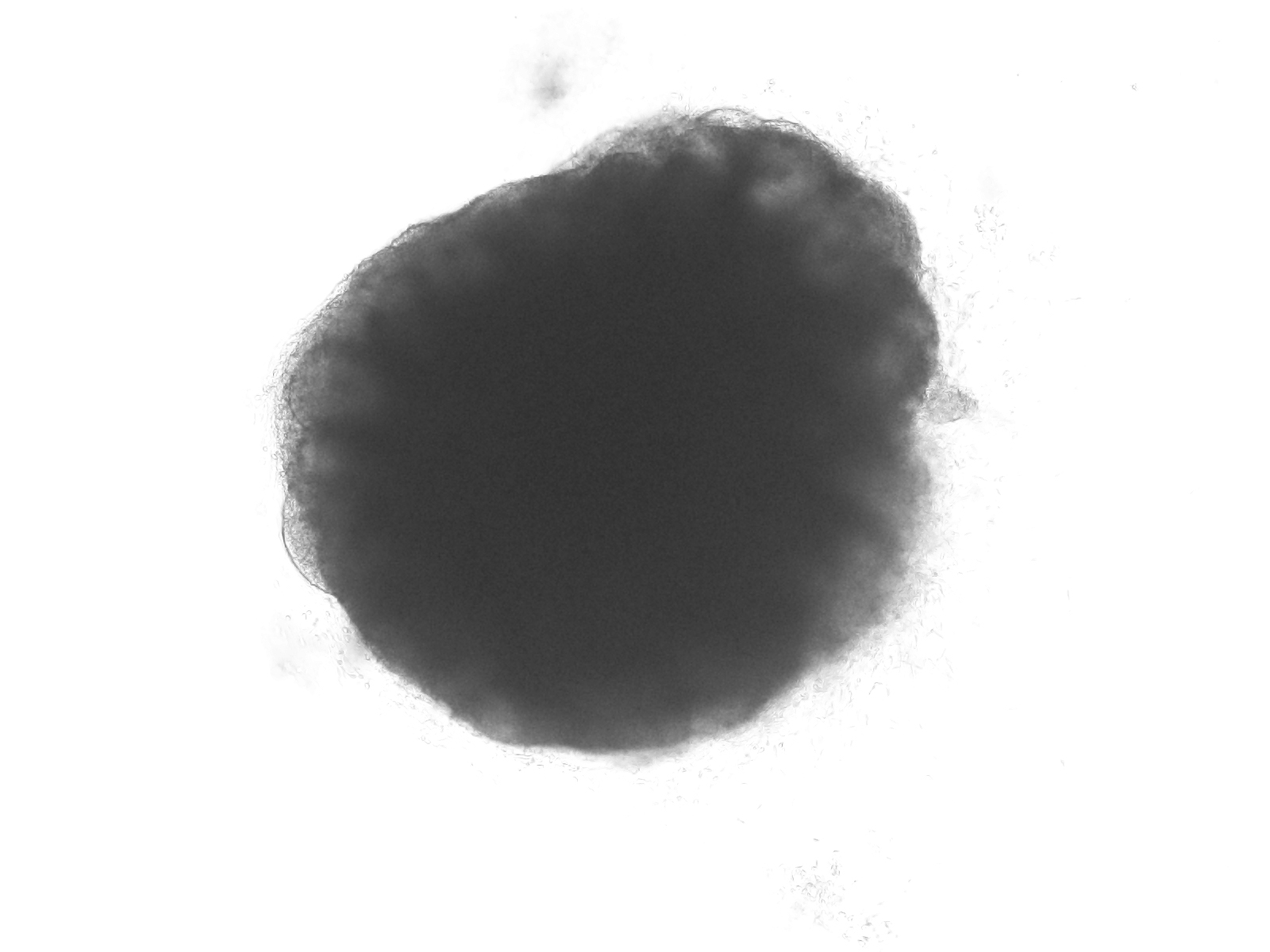

Supplement: Supplementary file 5 — Source data Fig. 3 [file 44321_2025_304_MOESM5_ESM.zip › Fig 3/Fig 3A/MBD2 d0.tif]

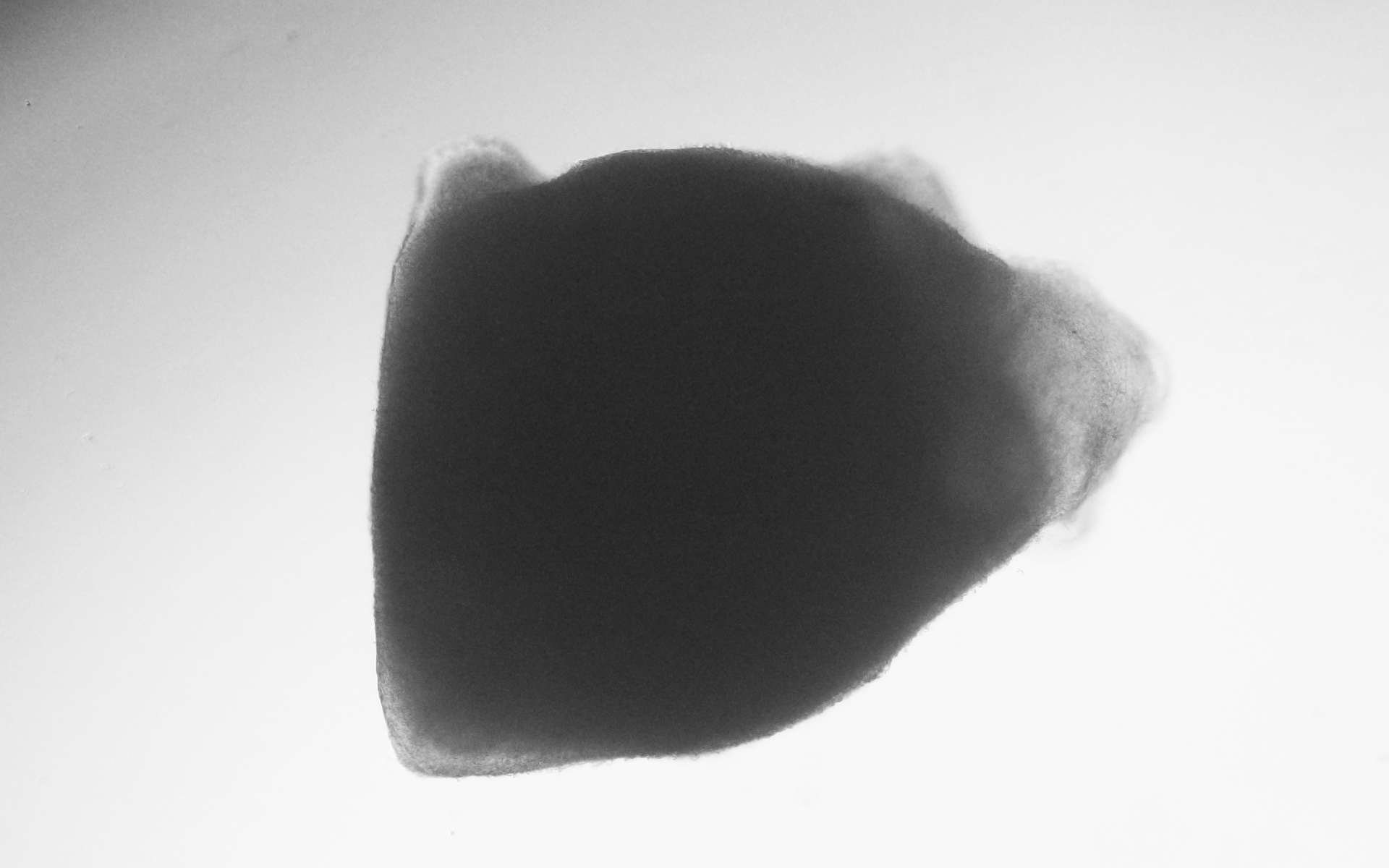

Supplement: Supplementary file 5 — Source data Fig. 3 [file 44321_2025_304_MOESM5_ESM.zip › Fig 3/Fig 3A/WT d18.tif]

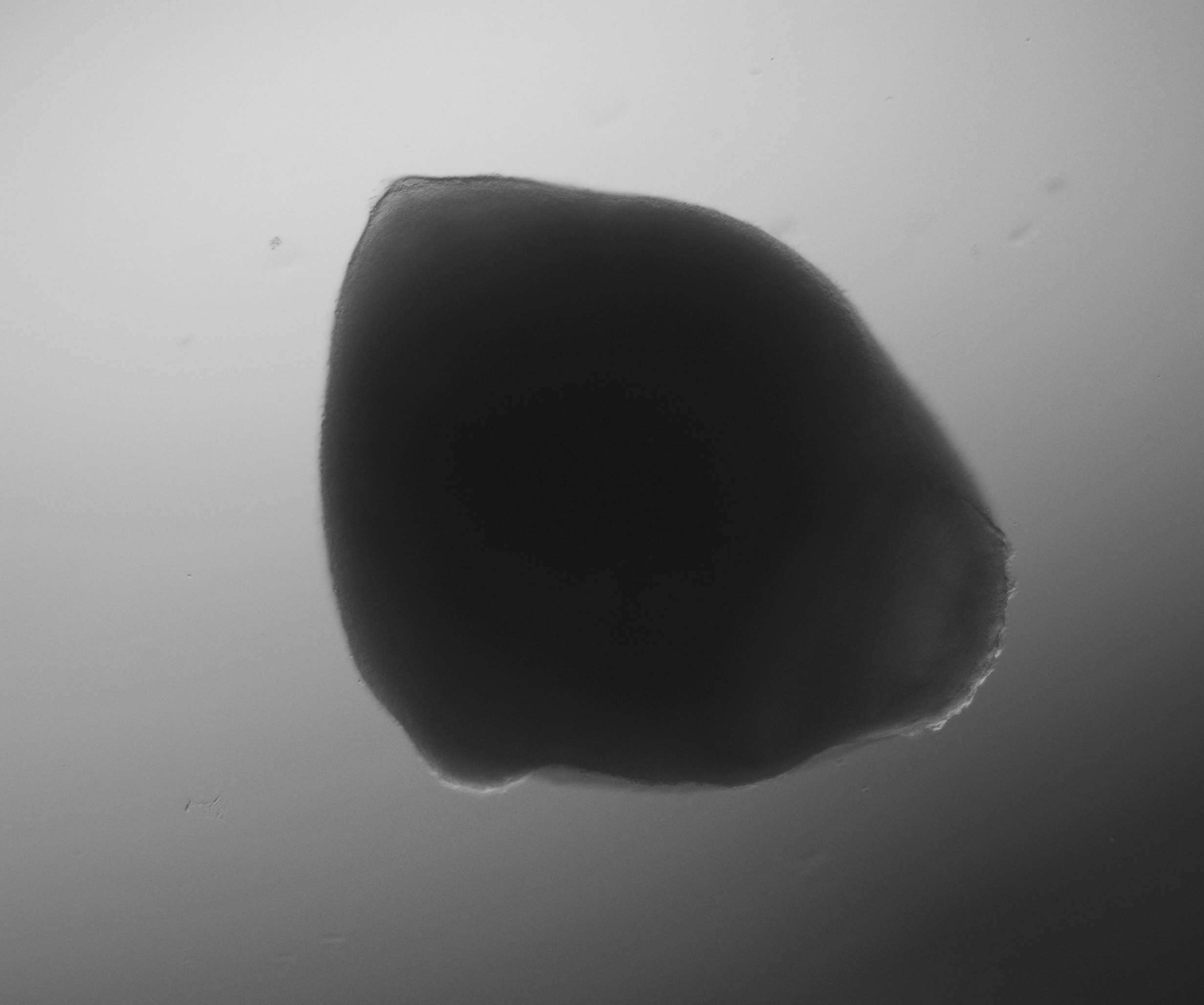

Supplement: Supplementary file 5 — Source data Fig. 3 [file 44321_2025_304_MOESM5_ESM.zip › Fig 3/Fig 3A/WT d30.tif]

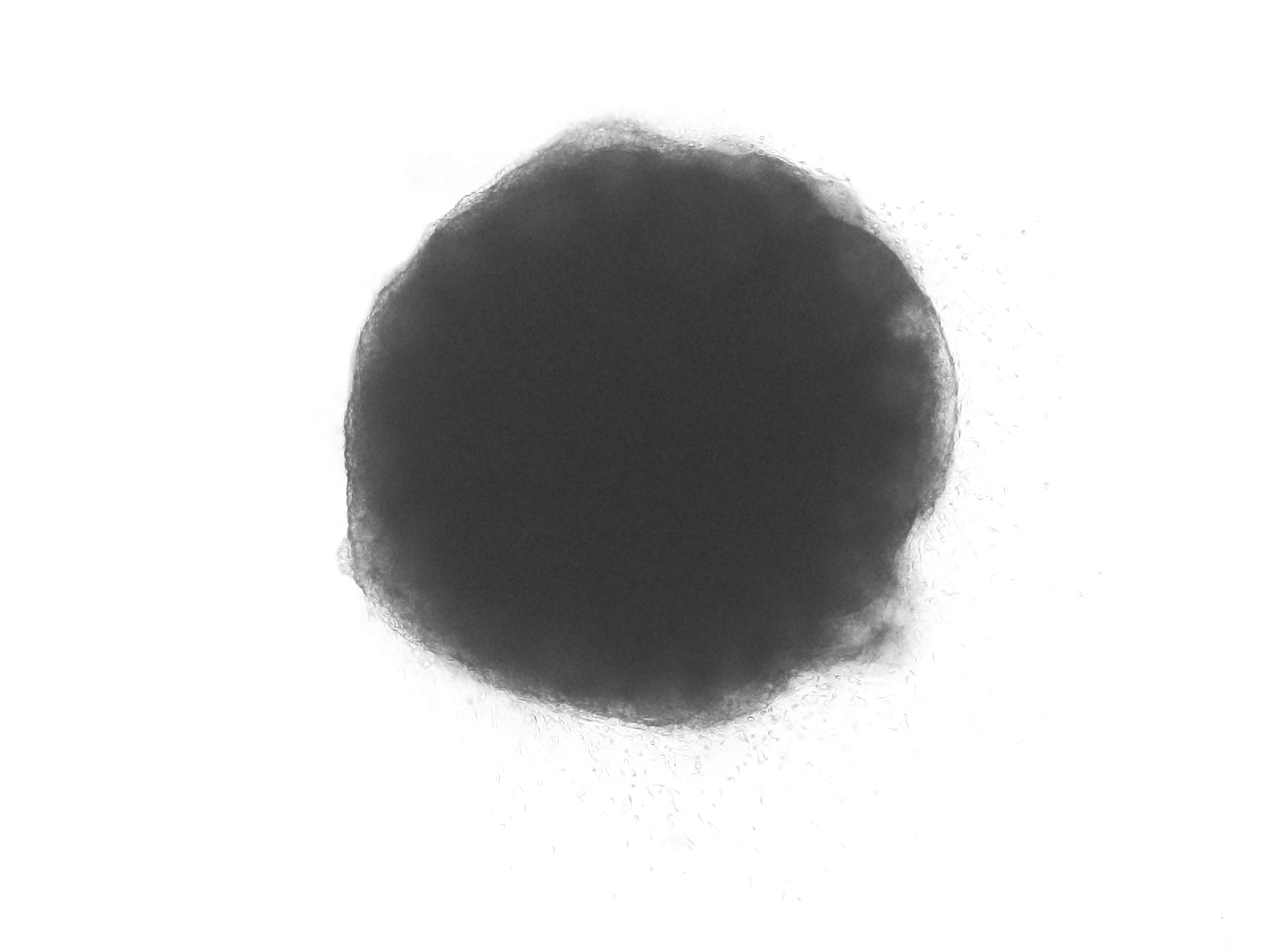

Supplement: Supplementary file 5 — Source data Fig. 3 [file 44321_2025_304_MOESM5_ESM.zip › Fig 3/Fig 3A/Mock d0.tif]

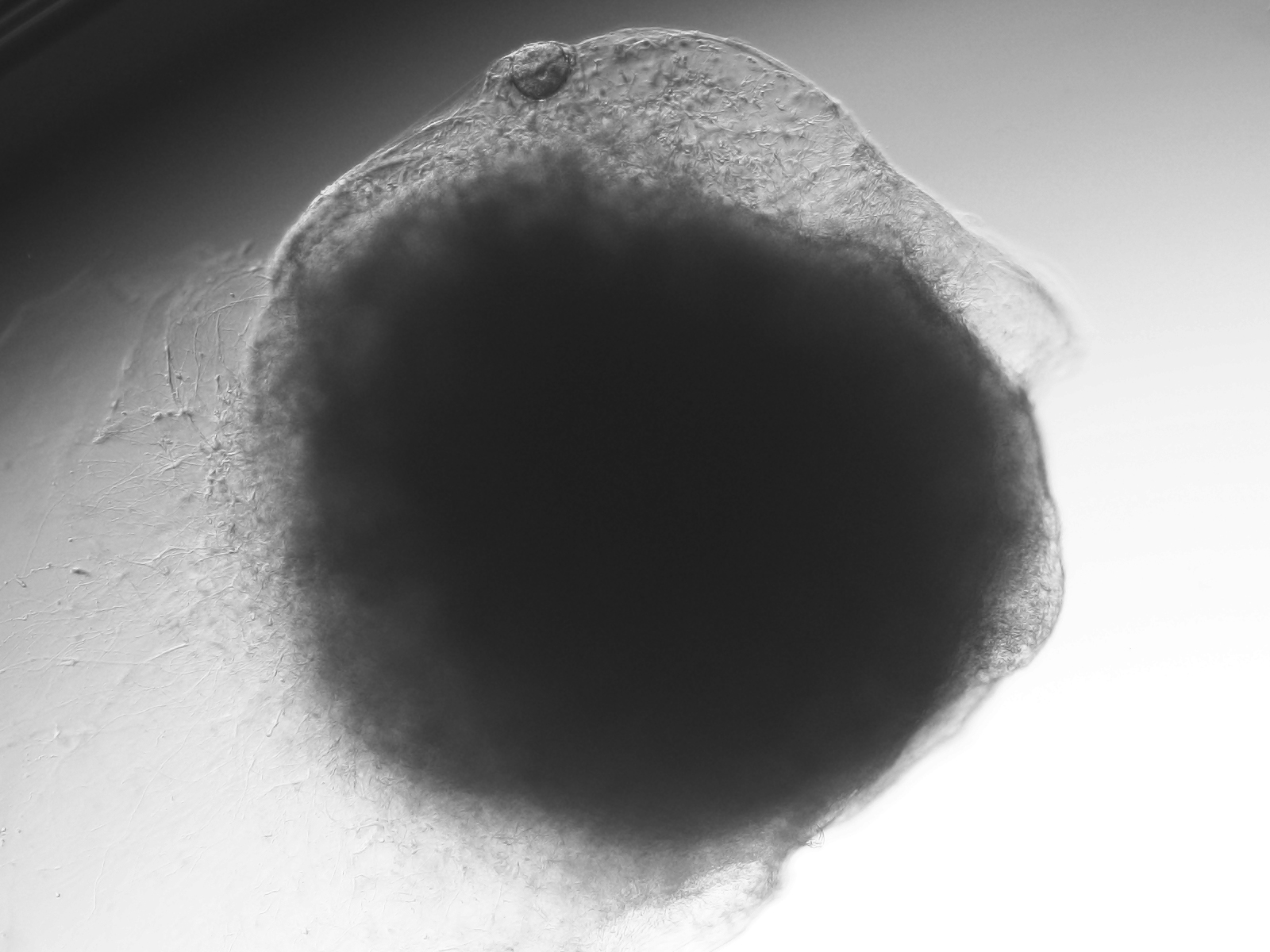

Supplement: Supplementary file 5 — Source data Fig. 3 [file 44321_2025_304_MOESM5_ESM.zip › Fig 3/Fig 3A/MBD2 d5.tif]

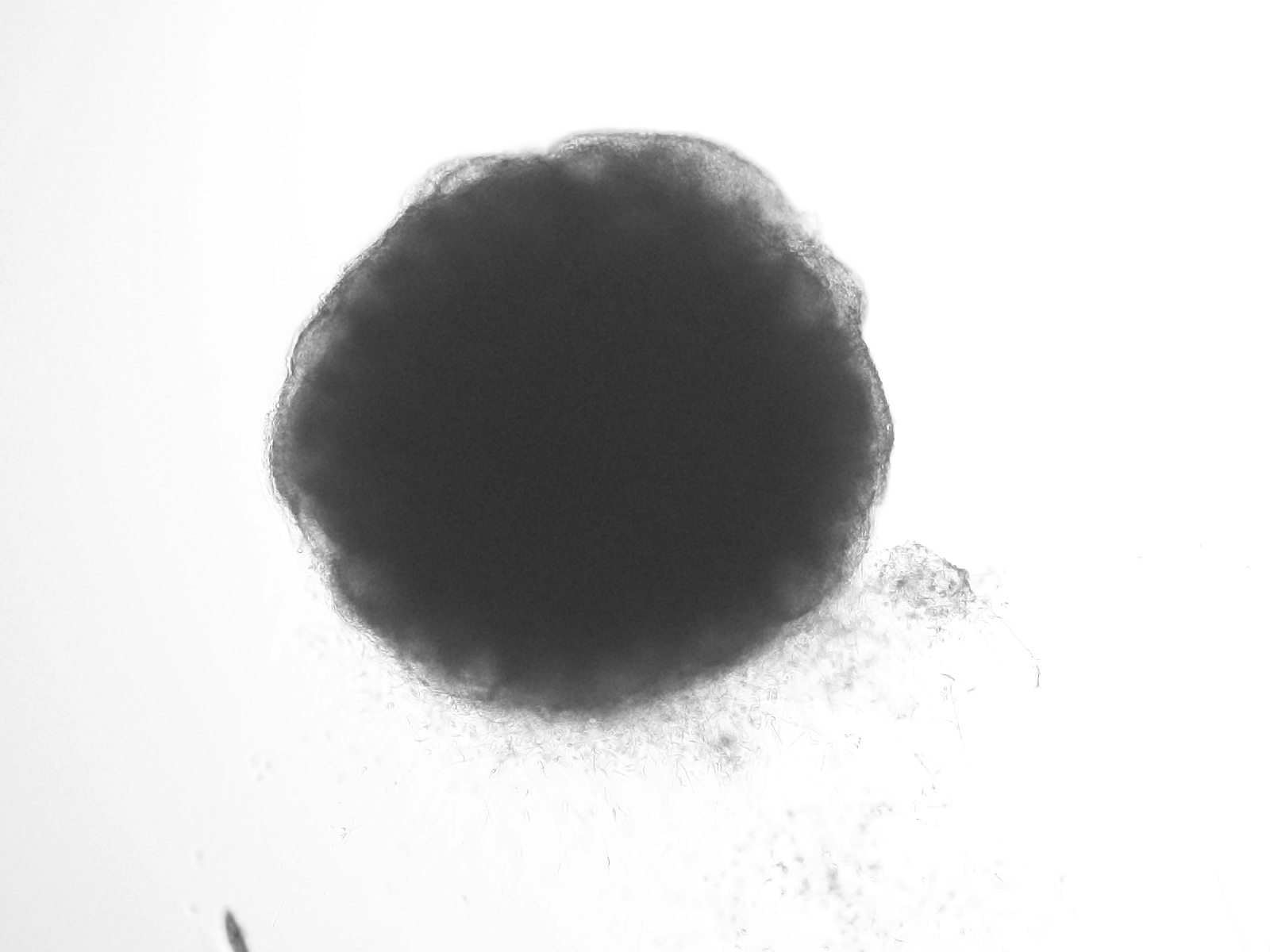

Supplement: Supplementary file 5 — Source data Fig. 3 [file 44321_2025_304_MOESM5_ESM.zip › Fig 3/Fig 3A/WT d0.tif]

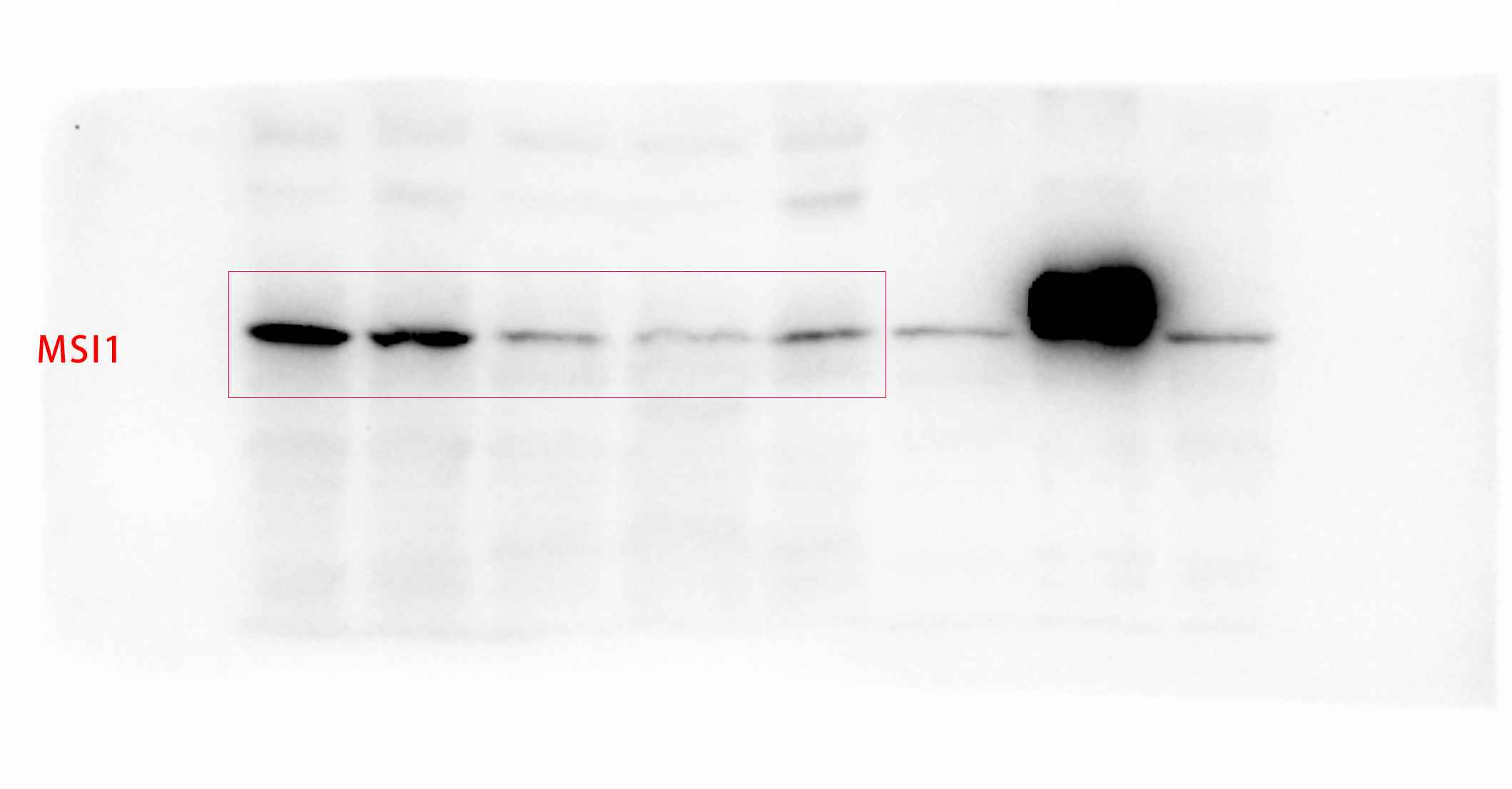

Supplement: Supplementary file 6 — Source data Fig. 4 [file 44321_2025_304_MOESM6_ESM.zip › Fig 4/Fig 4B/Western MSI1.tif]

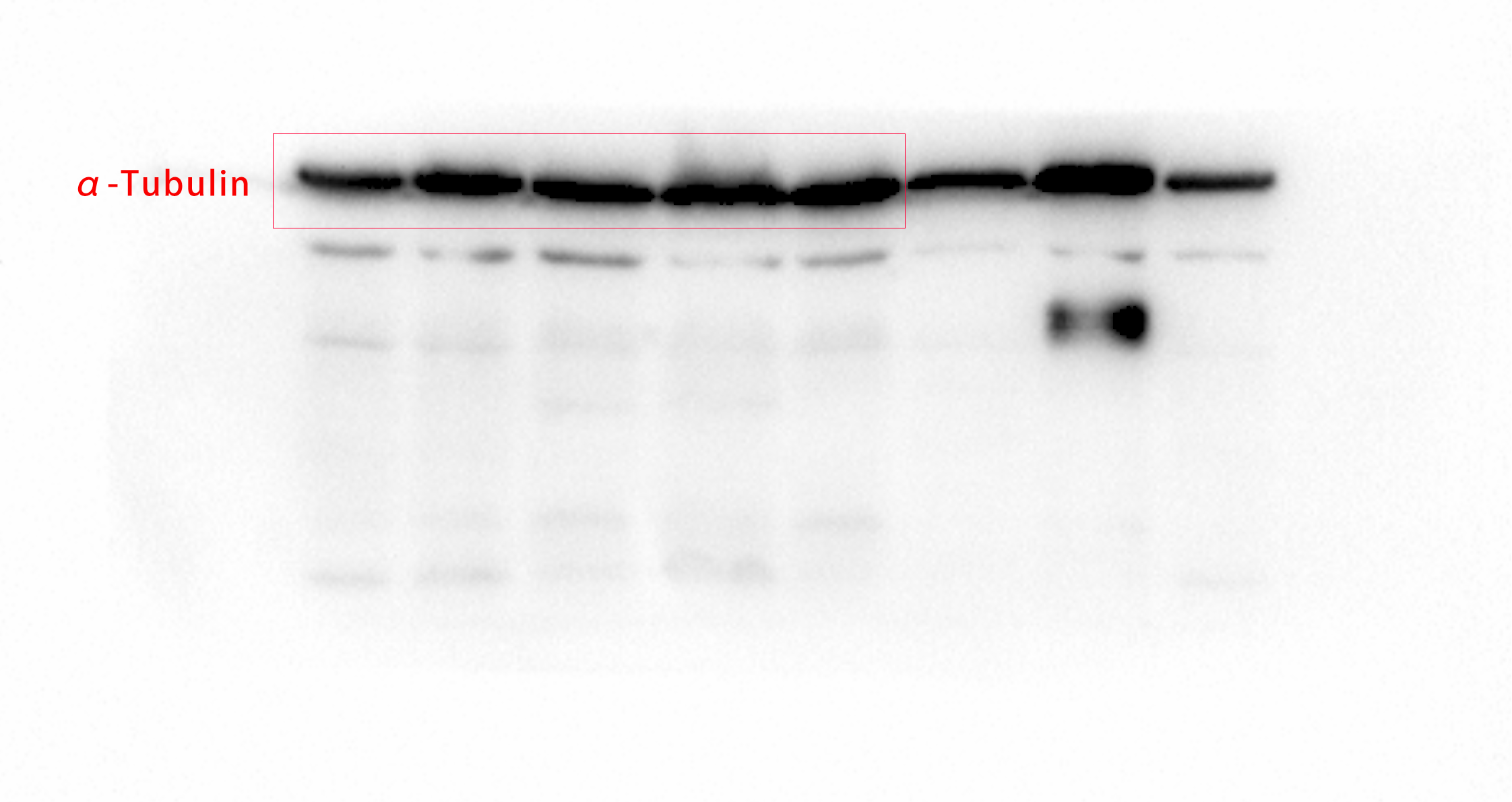

Supplement: Supplementary file 6 — Source data Fig. 4 [file 44321_2025_304_MOESM6_ESM.zip › Fig 4/Fig 4B/Western ╬▒-Tubulin.tif]

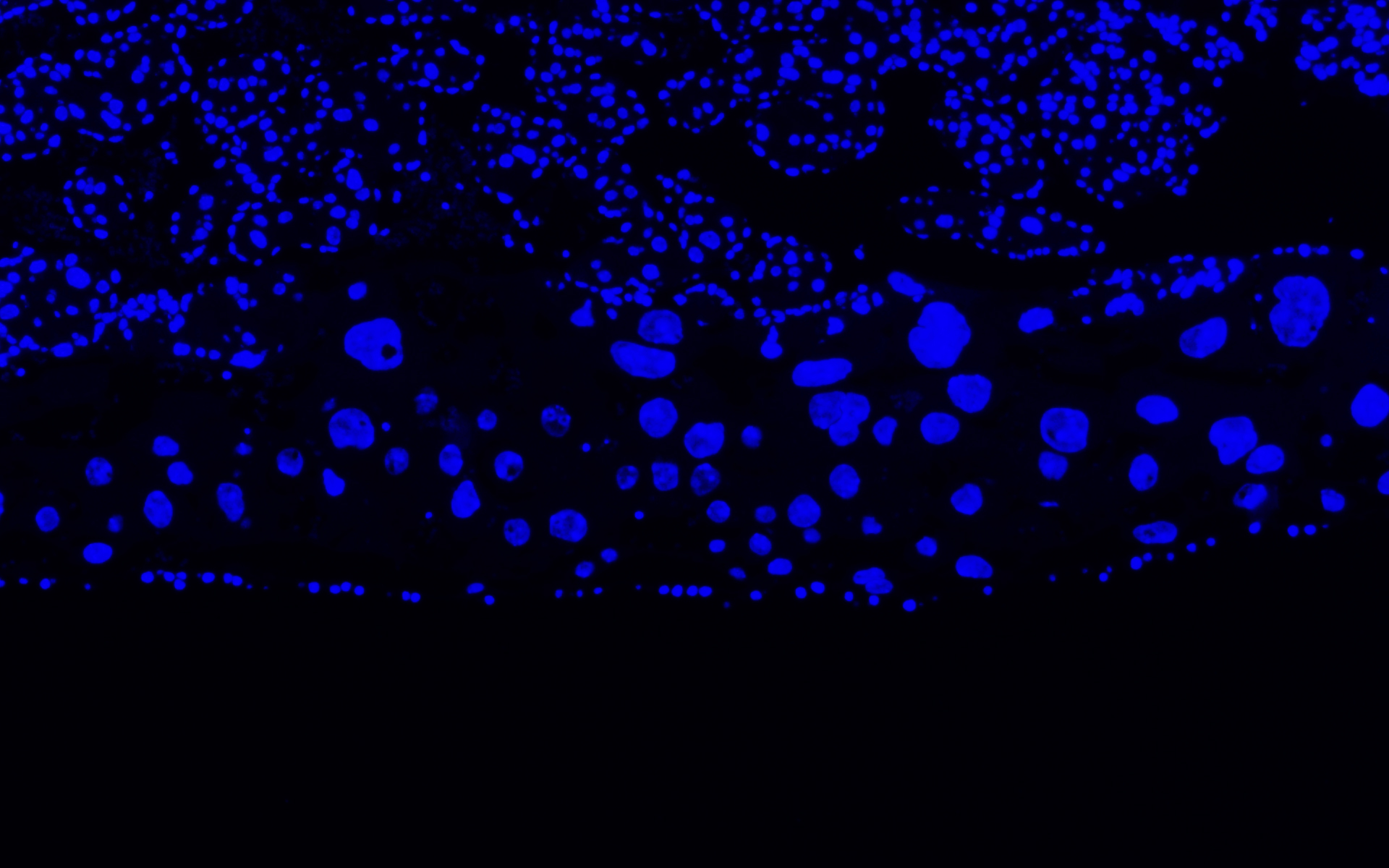

Supplement: Supplementary file 6 — Source data Fig. 4 [file 44321_2025_304_MOESM6_ESM.zip › Fig 4/Fig 4A/E10.5 DAPI.tif]

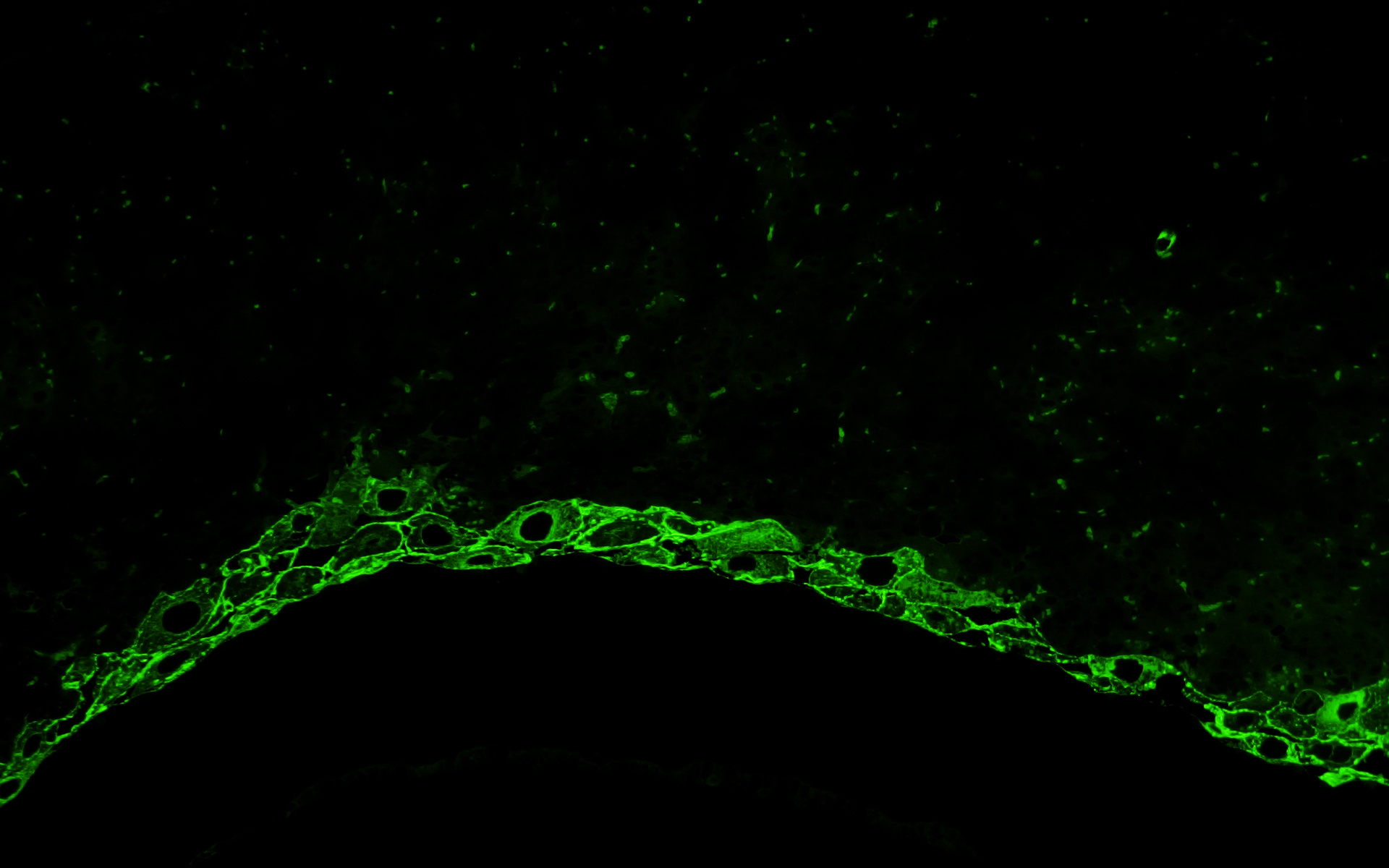

Supplement: Supplementary file 6 — Source data Fig. 4 [file 44321_2025_304_MOESM6_ESM.zip › Fig 4/Fig 4A/E8.5 CK7.tif]

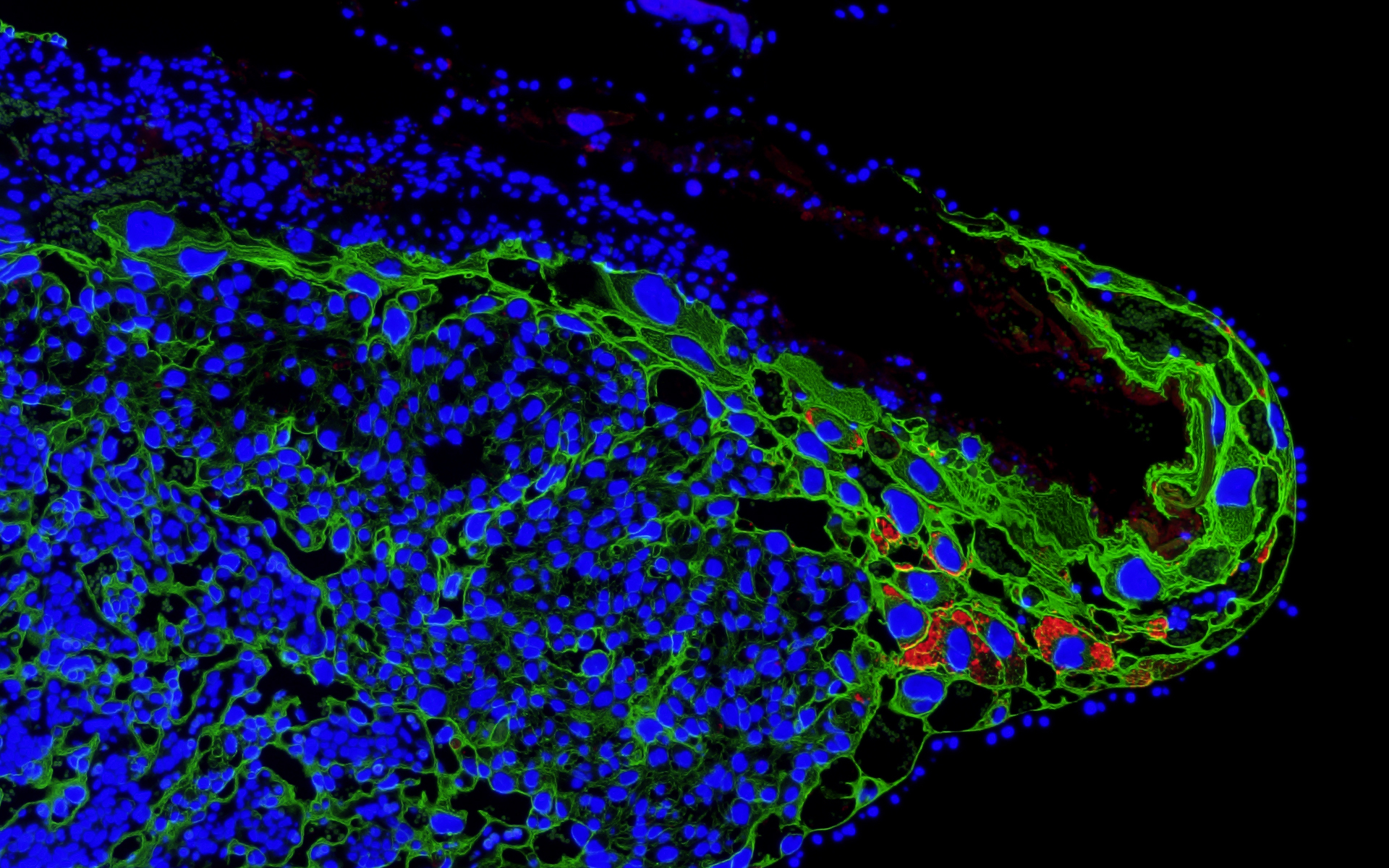

Supplement: Supplementary file 6 — Source data Fig. 4 [file 44321_2025_304_MOESM6_ESM.zip › Fig 4/Fig 4A/E12.5 merge.tif]

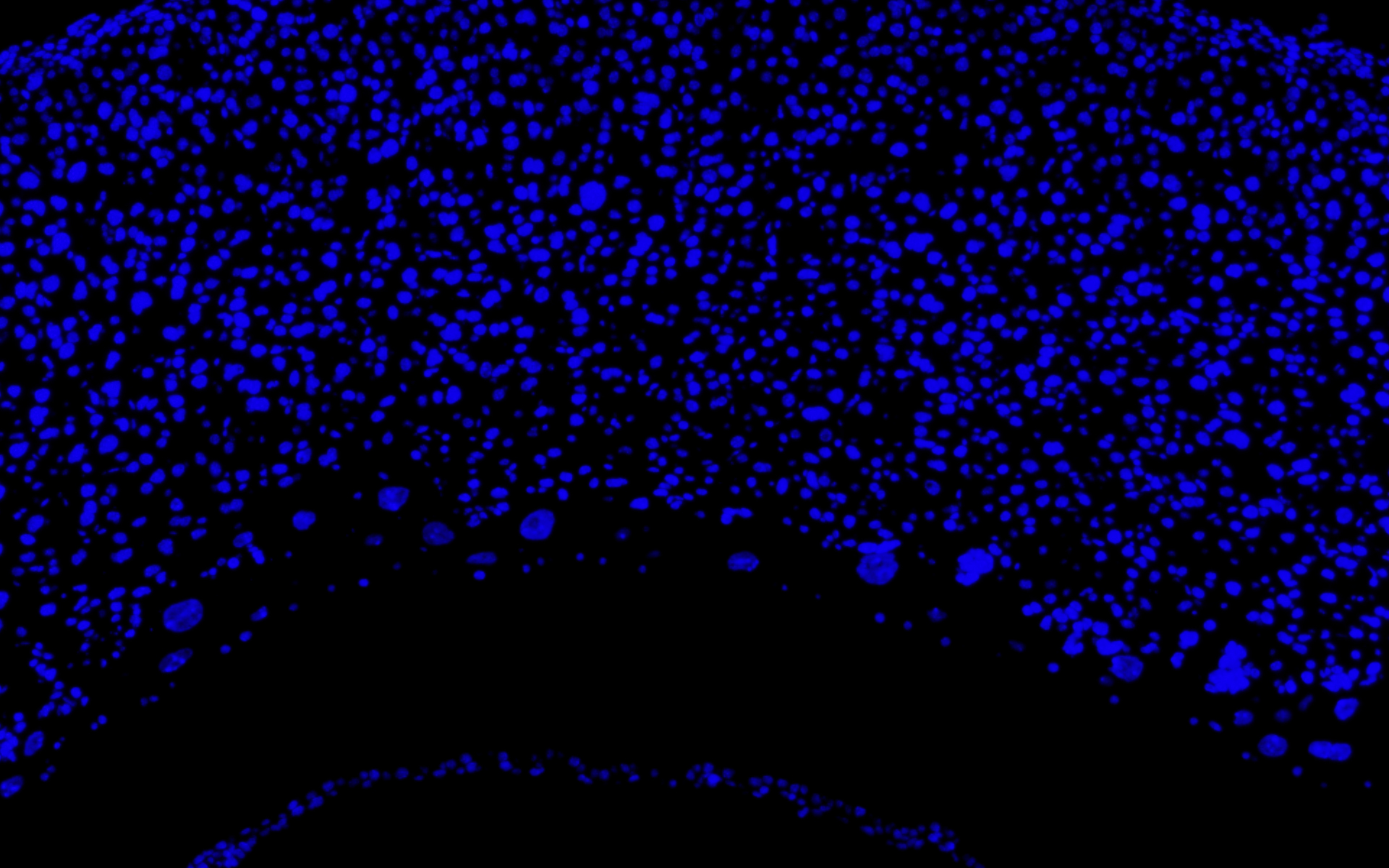

Supplement: Supplementary file 6 — Source data Fig. 4 [file 44321_2025_304_MOESM6_ESM.zip › Fig 4/Fig 4A/E8.5 DAPI.tif]

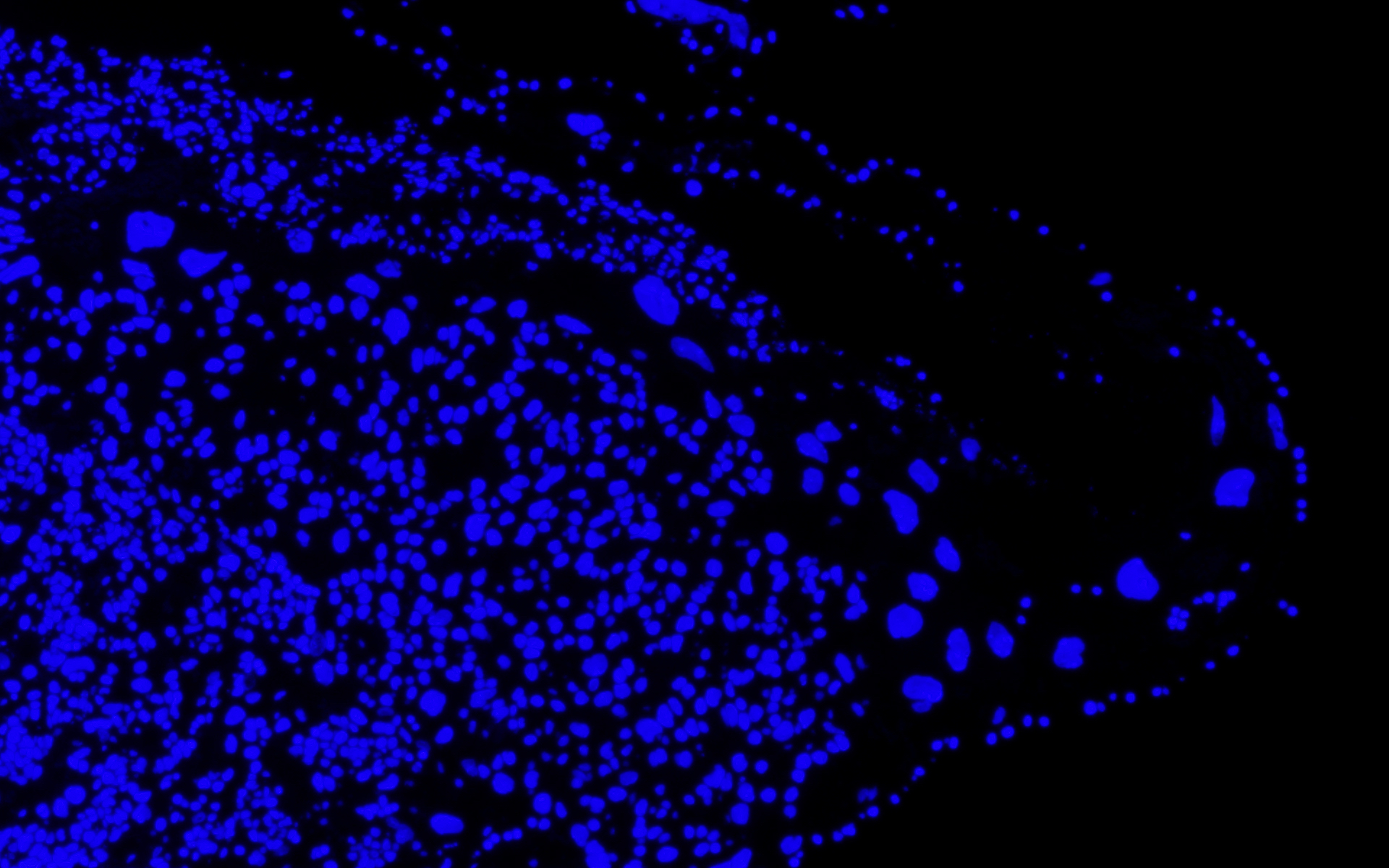

Supplement: Supplementary file 6 — Source data Fig. 4 [file 44321_2025_304_MOESM6_ESM.zip › Fig 4/Fig 4A/E12.5 DAPI.tif]

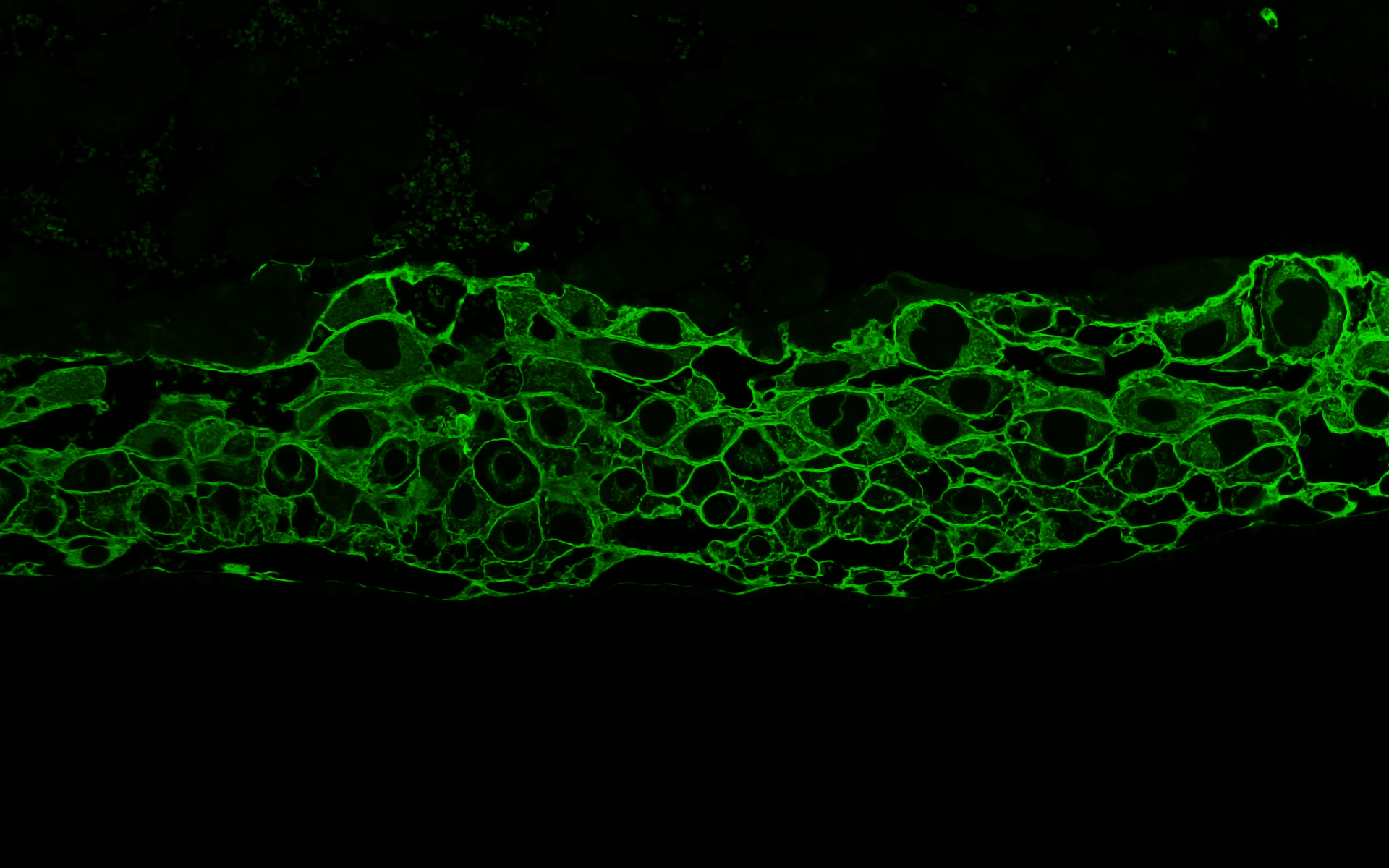

Supplement: Supplementary file 6 — Source data Fig. 4 [file 44321_2025_304_MOESM6_ESM.zip › Fig 4/Fig 4A/E10.5 CK7.tif]

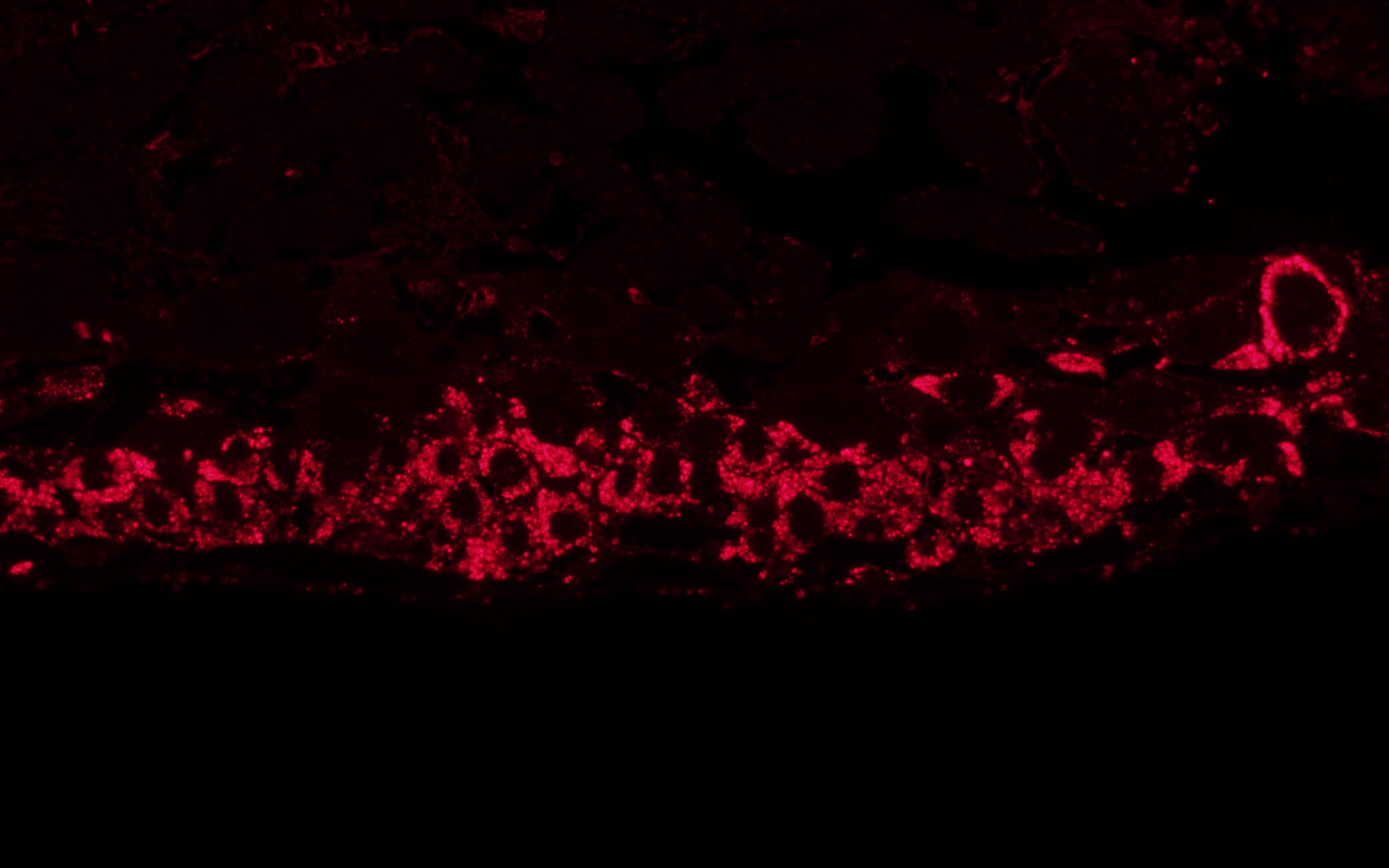

Supplement: Supplementary file 6 — Source data Fig. 4 [file 44321_2025_304_MOESM6_ESM.zip › Fig 4/Fig 4A/E10.5 MSI1.tif]

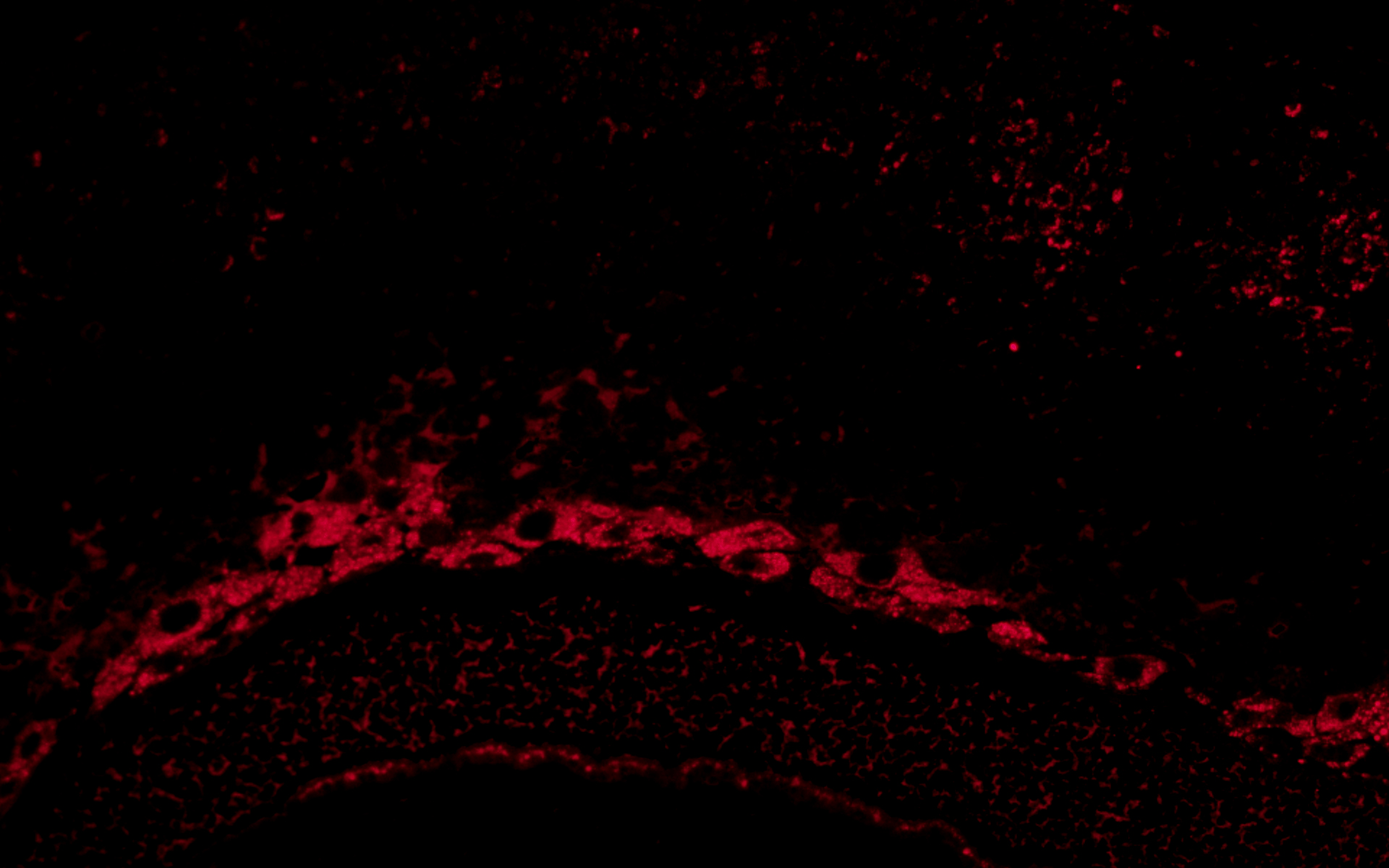

Supplement: Supplementary file 6 — Source data Fig. 4 [file 44321_2025_304_MOESM6_ESM.zip › Fig 4/Fig 4A/E8.5 MSI1.tif]

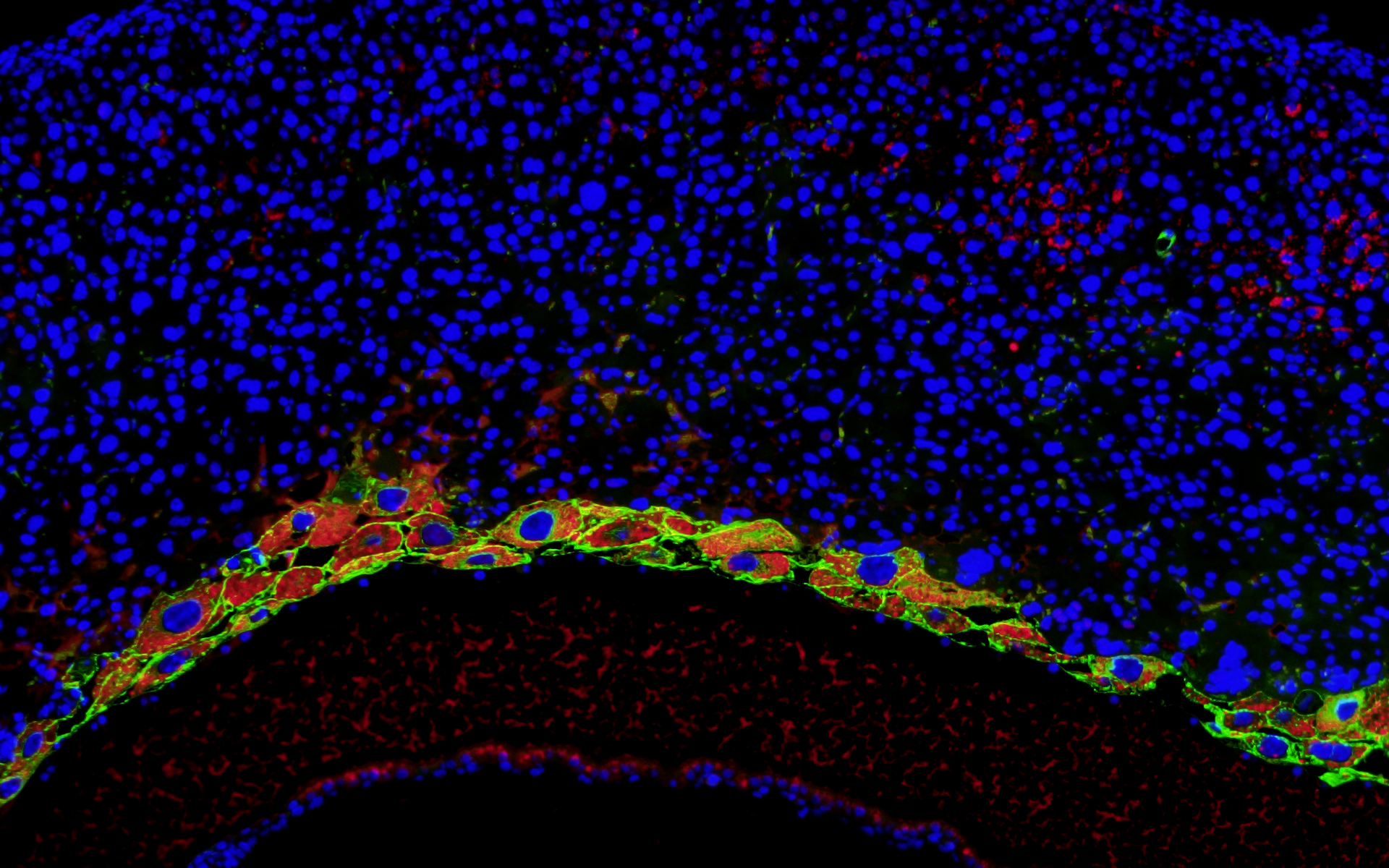

Supplement: Supplementary file 6 — Source data Fig. 4 [file 44321_2025_304_MOESM6_ESM.zip › Fig 4/Fig 4A/E8.5 merge.tif]

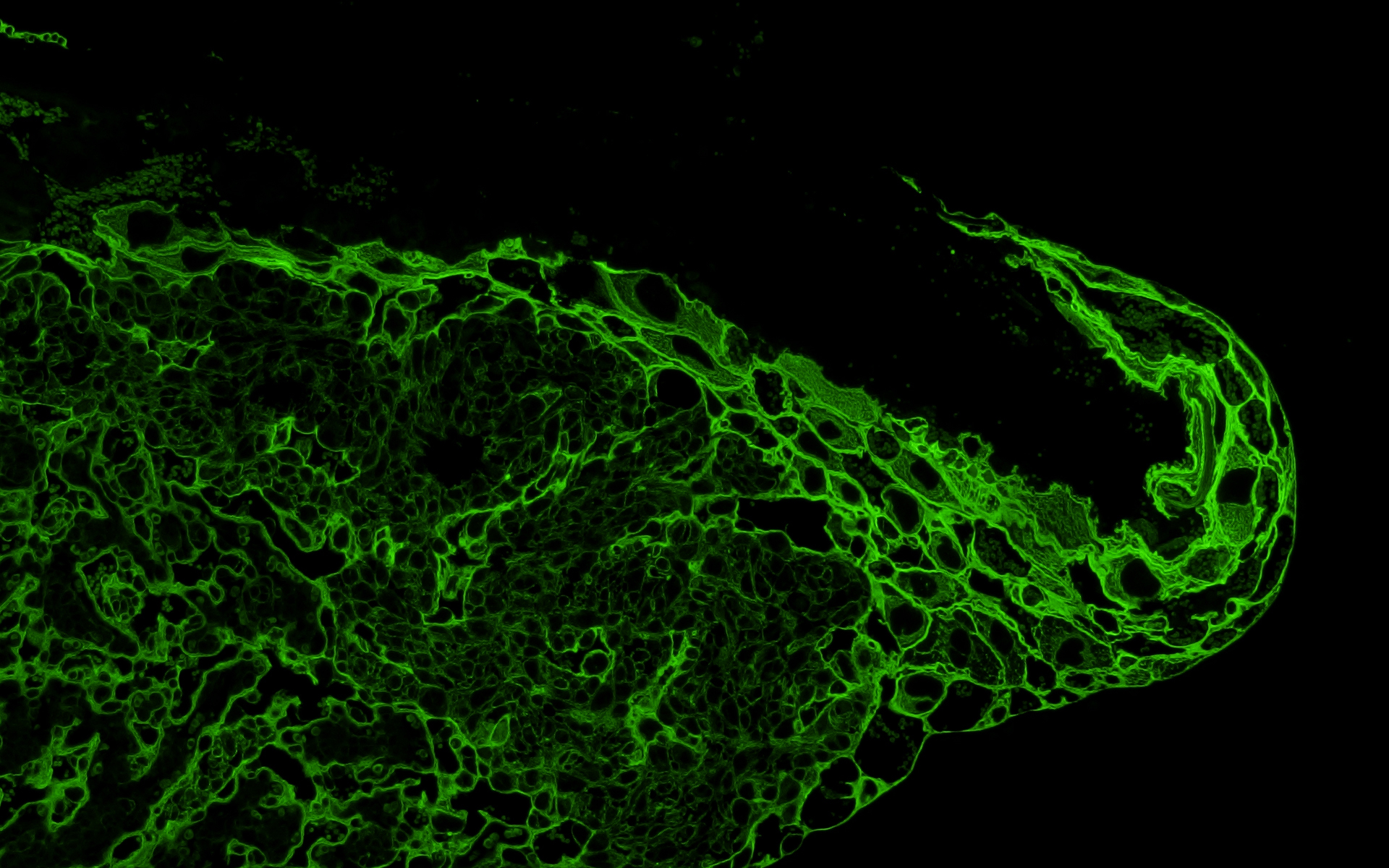

Supplement: Supplementary file 6 — Source data Fig. 4 [file 44321_2025_304_MOESM6_ESM.zip › Fig 4/Fig 4A/E12.5 CK7.tif]

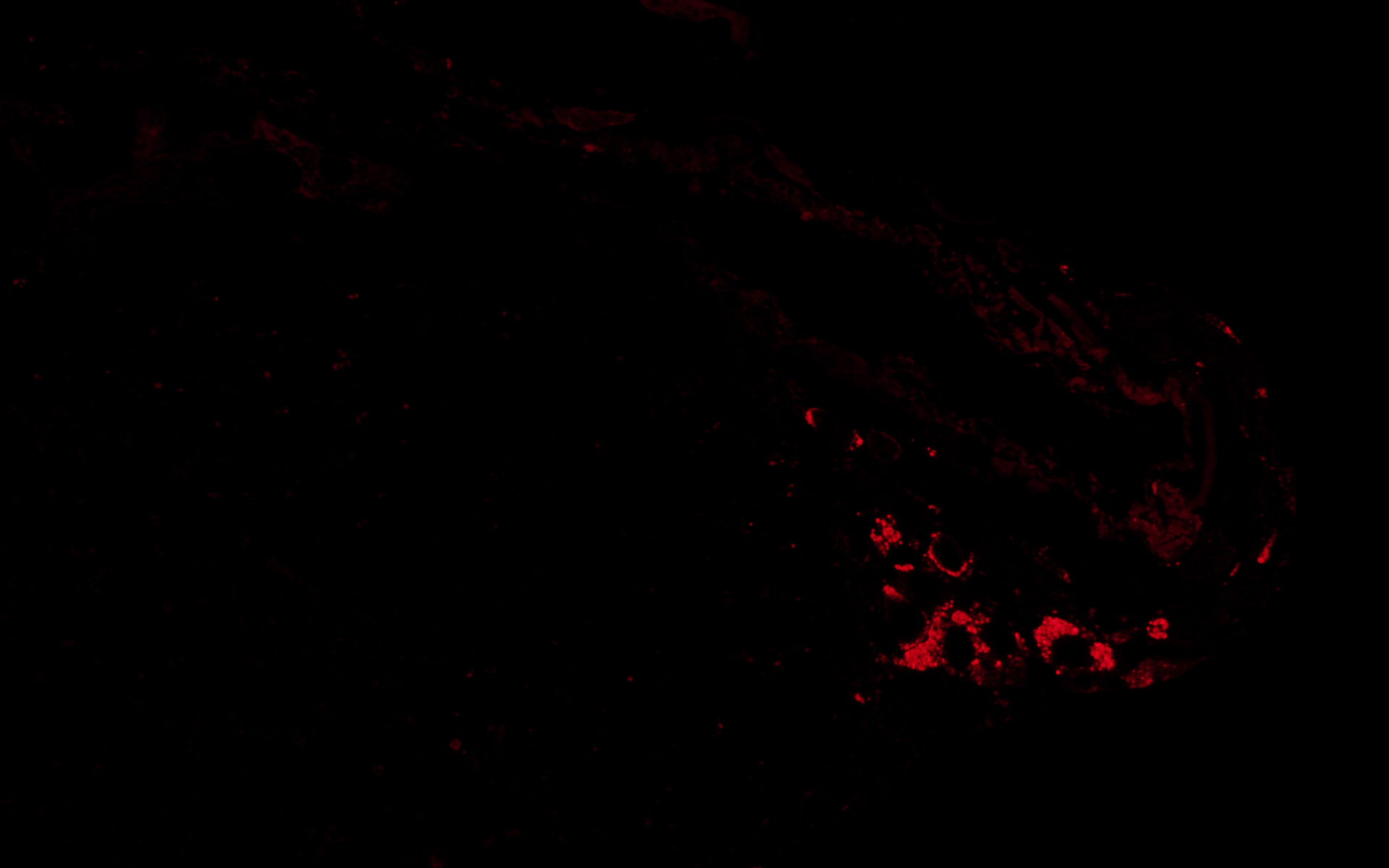

Supplement: Supplementary file 6 — Source data Fig. 4 [file 44321_2025_304_MOESM6_ESM.zip › Fig 4/Fig 4A/E12.5 MSI1.tif]

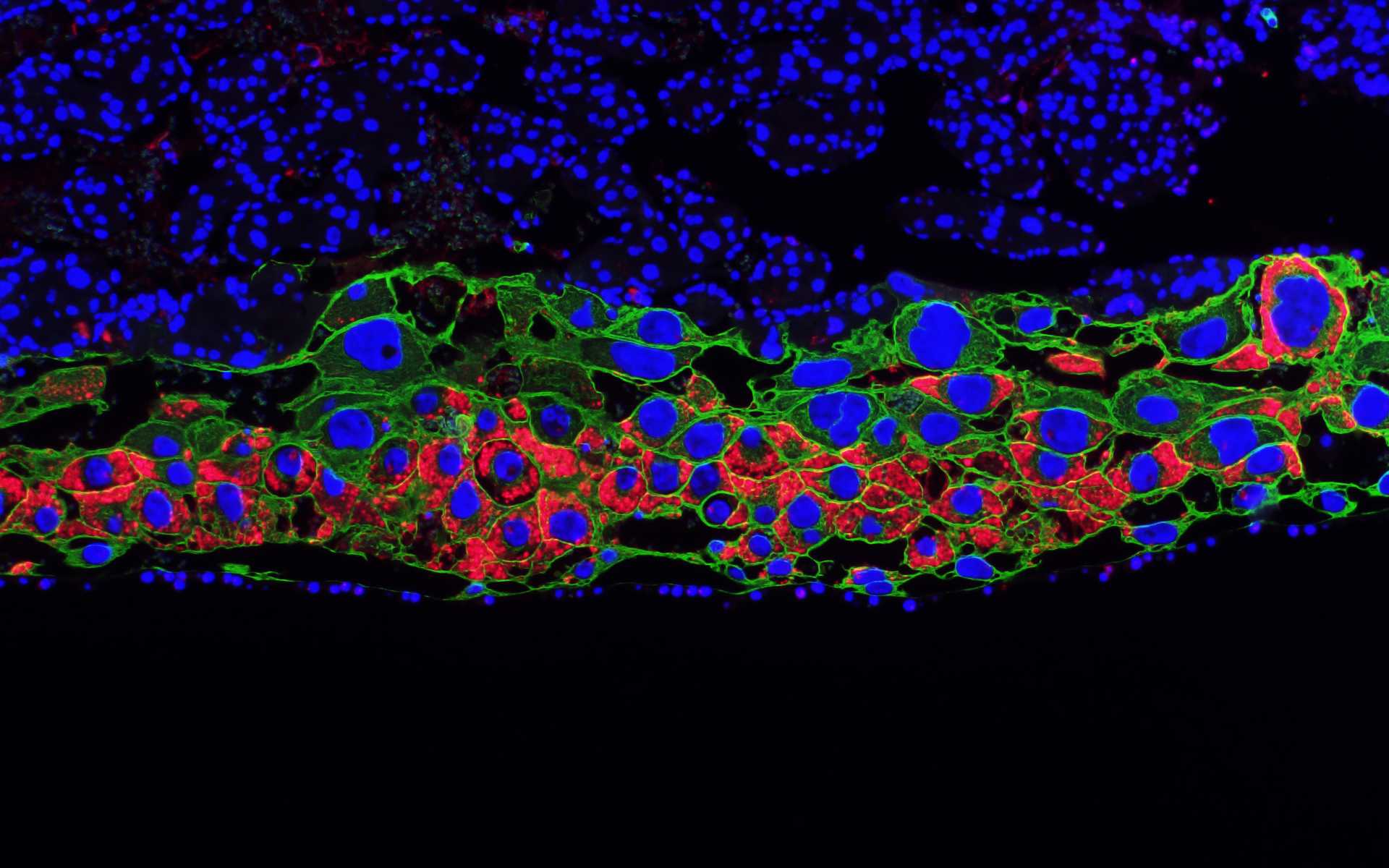

Supplement: Supplementary file 6 — Source data Fig. 4 [file 44321_2025_304_MOESM6_ESM.zip › Fig 4/Fig 4A/E10.5 merge.tif]

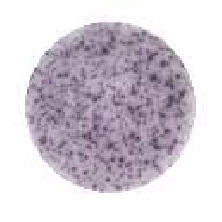

Supplement: Supplementary file 7 — Source data Fig. 5 [file 44321_2025_304_MOESM7_ESM.zip › Fig 5/Fig 5H/MBD2-ZIKVE.jpg]

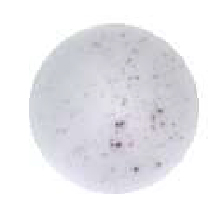

Supplement: Supplementary file 7 — Source data Fig. 5 [file 44321_2025_304_MOESM7_ESM.zip › Fig 5/Fig 5H/PBS-ZIKVE.jpg]

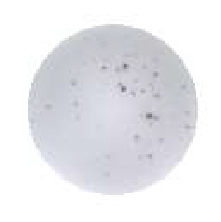

Supplement: Supplementary file 7 — Source data Fig. 5 [file 44321_2025_304_MOESM7_ESM.zip › Fig 5/Fig 5H/MBD2-no treat.jpg]

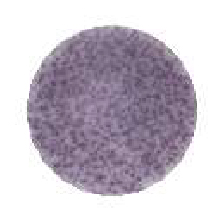

Supplement: Supplementary file 7 — Source data Fig. 5 [file 44321_2025_304_MOESM7_ESM.zip › Fig 5/Fig 5H/MBD1-ConA.jpg]

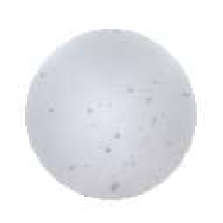

Supplement: Supplementary file 7 — Source data Fig. 5 [file 44321_2025_304_MOESM7_ESM.zip › Fig 5/Fig 5H/PBS-no treat.jpg]

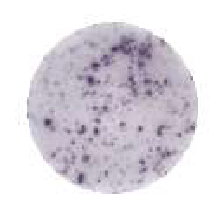

Supplement: Supplementary file 7 — Source data Fig. 5 [file 44321_2025_304_MOESM7_ESM.zip › Fig 5/Fig 5H/PBS-ConA.jpg]

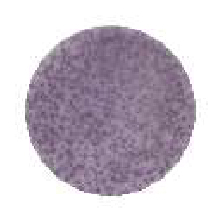

Supplement: Supplementary file 7 — Source data Fig. 5 [file 44321_2025_304_MOESM7_ESM.zip › Fig 5/Fig 5H/MBD2-ConA.jpg]

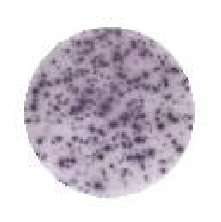

Supplement: Supplementary file 7 — Source data Fig. 5 [file 44321_2025_304_MOESM7_ESM.zip › Fig 5/Fig 5H/MBD1-ZIKVE.jpg]

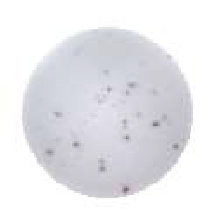

Supplement: Supplementary file 7 — Source data Fig. 5 [file 44321_2025_304_MOESM7_ESM.zip › Fig 5/Fig 5H/MBD1-no treat.jpg]
