# Supplementary material for: Manipulating Zika virus RNA tertiary structure for developing tissue-specific attenuated vaccines
Source: EMBO Mol Med. 2025 Sep 8;17(10):2787–808. doi: 10.1038/s44321-025-00304-5 (PMC12514043; doi:10.1038/s44321-025-00304-5)
Supplement: Supplementary file 9 — Expanded View Figures [file 44321_2025_304_MOESM9_ESM.pdf]

# Expanded View Figures

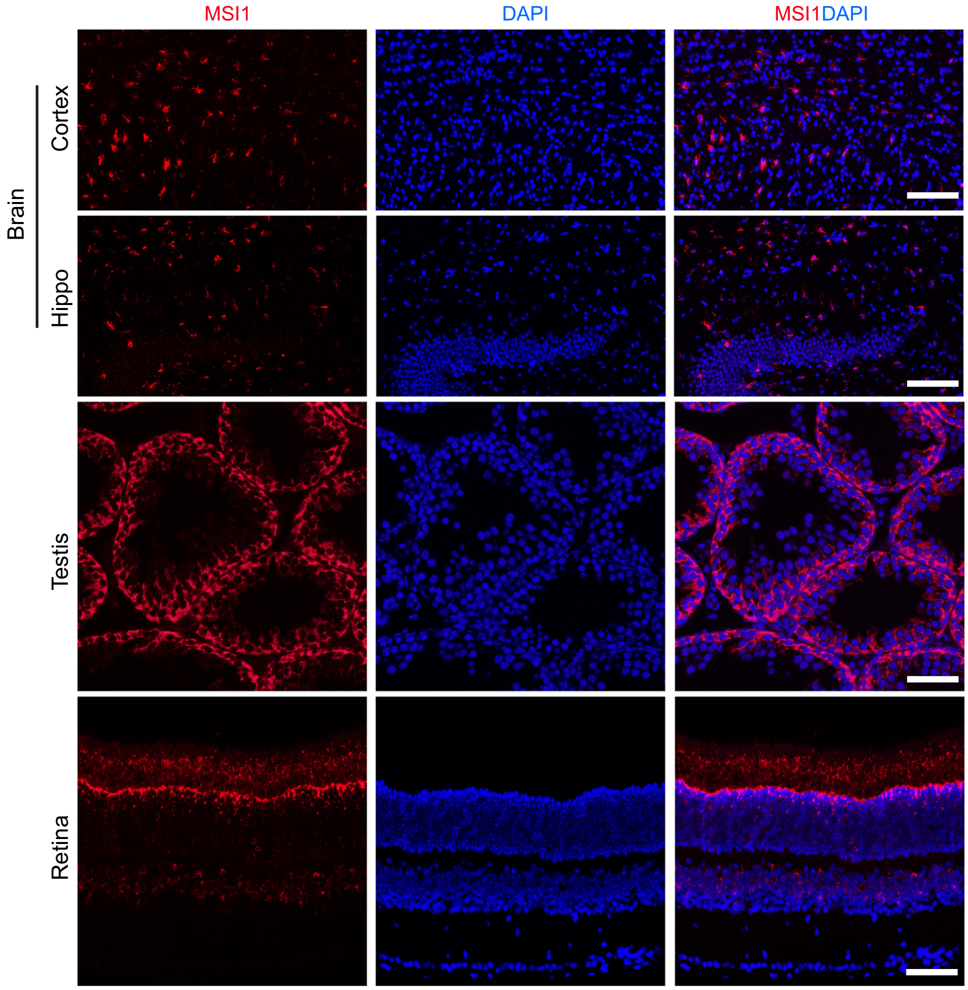


**Figure EV1. Characterization of MSI1 expression in tissues vulnerable to ZIKV.**

Brain, testis and retina sections of 4-week-old A129 mice were stained with an anti-MSI1 antibody. Scale bar, 100 μm.

 © The Author(s)

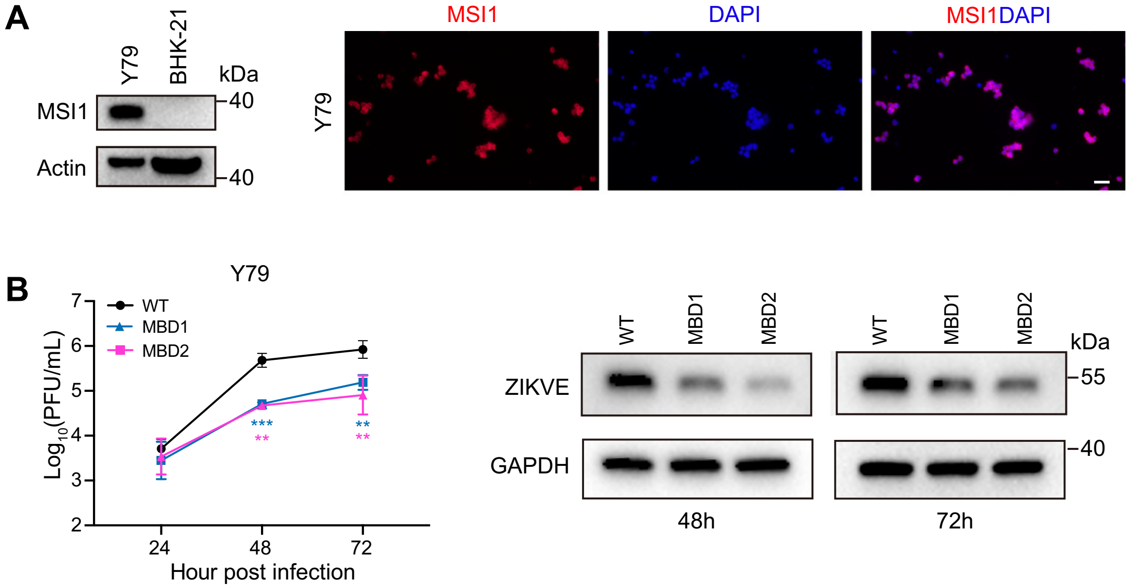


**Figure EV2. MBD ZIKV exhibits attenuated replication kinetics in a human retina cell line expressing MSI1.**

(**A**) MSI1 expression in human retinoblastoma Y79 cells was detected by Western blotting (left panel) and immunostaining (right panel). Scale bar, 50 μm. (**B**) Y79 cells were infected with WT ZIKV, MBD1 or MBD2 (MOI = 1), and the culture supernatants were harvested at the indicated time points for detection of viral loads by plaque forming assay (left panel). Data are mean ± SD. $n = 3$. $n$ represents biological replicates. Two-way ANOVA, $^{**}P < 0.01$, $^{***}P < 0.001$ (48: MBD1 $P = 0.0007$, MBD2 $P = 0.0023$; 72: MBD1 $P = 0.0058$, MBD2 $P = 0.0021$). The expression of ZIKV-E protein at 48 h and 72 h after infection was detected by Western blotting (right panel).

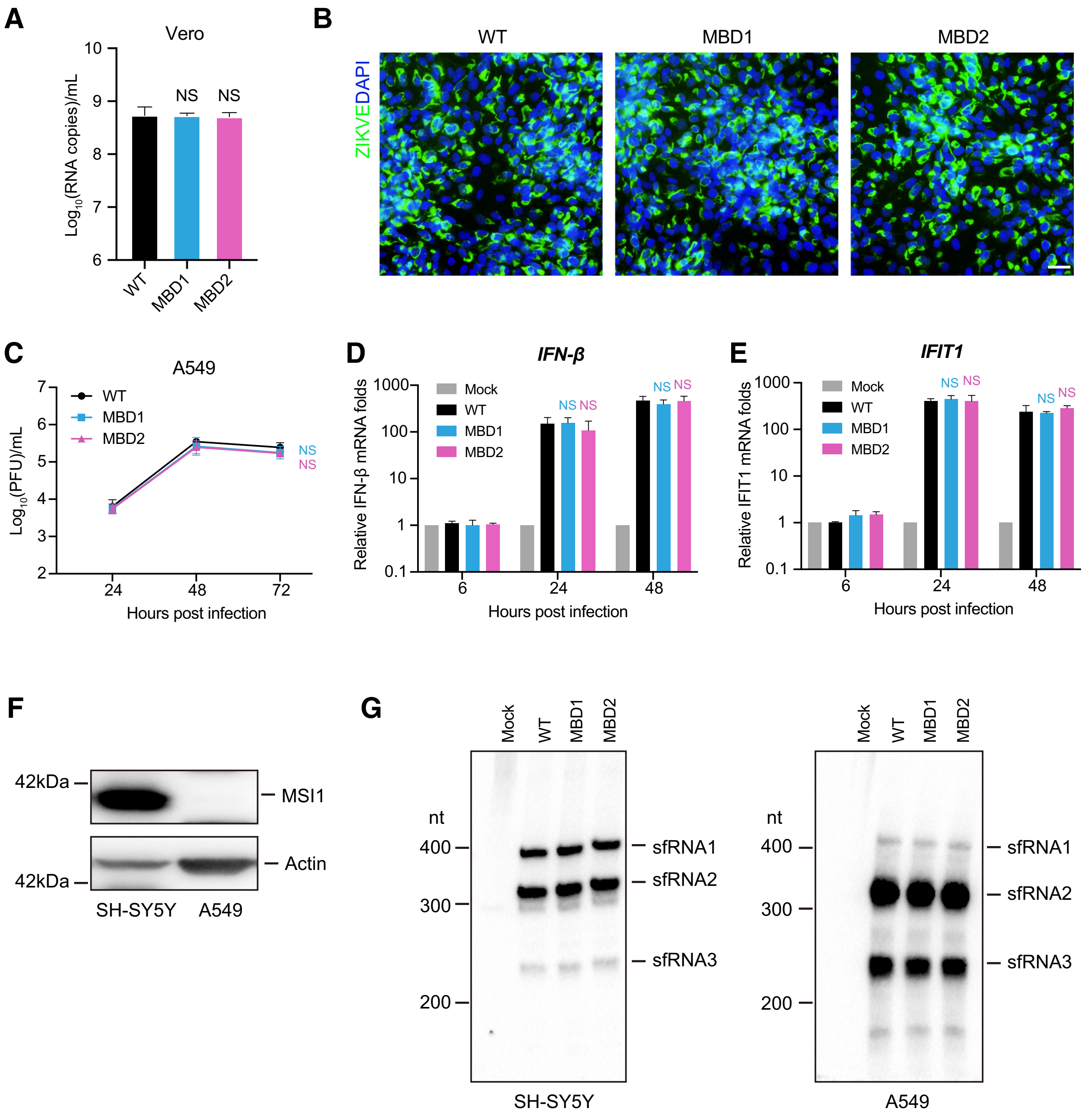


**Figure EV3. The attenuation of MBD ZIKV is independent of innate immune or sfRNA.**

(**A**) Vero cells were infected with WT ZIKV, MBD1 or MBD2 (MOI = 0.1), and the viral RNA loads in culture supernatants at 72 h after infection were detected by RT-qPCR. Data are mean ± SD. $n = 3$. $n$ represents biological replicates. Two-sided Student's $t$ test. NS, not significant. (**B**) The expression of ZIKV-E protein at 72 h after infection was detected by immunostaining. Scale bar, 50 μm. (**C**) A549 cells were infected with WT ZIKV, MBD1 or MBD2 (MOI = 0.1), and the culture supernatants were harvested at the indicated time points for detection of viral loads by plaque forming assay (left panel). Data are mean ± SD. $n = 3$. $n$ represents biological replicates. Two-way ANOVA, NS not significant. (**D**, **E**) Relative levels of *IFN-β* (**D**) and *IFIT1* (**E**) mRNA of infected A549 cells at indicated times after infection determined by RT-qPCR. The value of mock infected at 6 h post infection was set as 1. Data are the mean ± SD. $n = 3$. $n$ represents biological replicates. Two-sided Student's $t$ test. NS not significant. (**F**) The expression of MSI1 protein of SH-SY5Y and A549 cells was detected by Western blotting. (**G**) The sfRNA expression in SH-SY5Y and A549 cells after infection was detected by Northern blotting.

 © The Author(s)

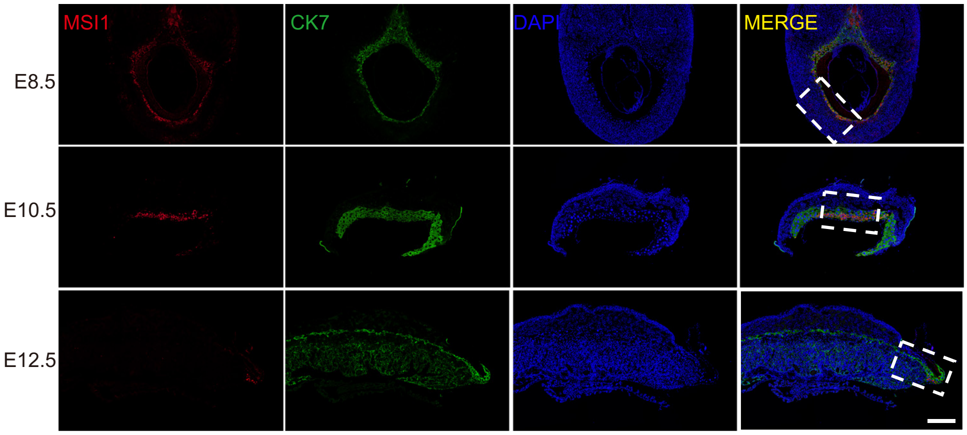


**Figure EV4. Characterization of MSI1 expression in the placenta during mouse pregnancy.**

Overview of immunostaining of mouse placenta sections at embryonic stage 8.5 (E8.5), E10.5, and E12.5. Tissue sections were stained with anti-MSI1(red) antibody and anti-CK7 antibody (green), a trophoblast cell marker. Nuclei were stained with DAPI (blue). The boxed areas are shown at higher magnification in Fig. 4A. Scale bar, 500 µm.

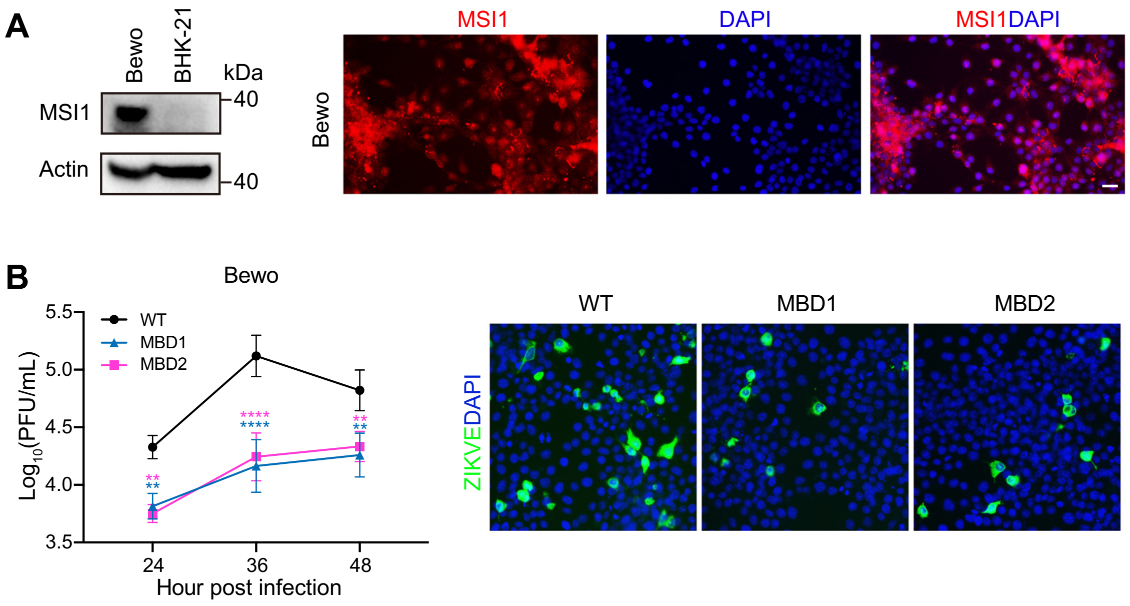


**Figure EV5. MBD ZIKV exhibits attenuated replication kinetics in a human placental cell line expressing MSI1.**

(**A**) MSI1 expression in human placenta choriocarcinoma Bewo cells was detected by Western blotting (left panel) and immunostaining (right panel). Scale bar, 50 μm. (**B**) Bewo cells were infected with WT ZIKV, MBD1 or MBD2 (MOI = 1), and the culture supernatants were harvested at the indicated time points for detection of viral loads by plaque forming assay (left panel). Data are mean ± SD. $n = 3$. $n$ represents biological replicates. Two-way ANOVA, $^{**}P < 0.01$, $^{****}P < 0.0001$ (24: MBD1 $P = 0.0017$, MBD2 $P = 0.0093$; 36: MBD1 $P = 0.000041$, MBD2 $P = 0.000052$; 48: MBD1 $P = 0.0062$, MBD2 $P = 0.0049$). The expression of ZIKV-E protein at 36 h after infection was detected by immunostaining (right panel). Scale bar, 50 μm.

 © The Author(s)
